# Mental health stigma and its consequences: a systematic scoping review of pathways to discrimination and adverse outcomes

**DOI:** 10.1016/j.eclinm.2025.103588

**Published:** 2025-10-23

**Authors:** Anna Kågström, Zoe Guerrero, Akmal Alikhan Aliev, Hana Tomášková, Nicolas Rüsch, Uta Ouali, Graham Thornicroft, Norman Sartorius, Petr Winkler

**Affiliations:** a Department of Public Mental Health, National Institute of Mental Health, Topolová 748, 250 67, Klecany, Czech Republic; b WHO Collaborating Center for Public Mental Health Research and Service Development, National Institute of Mental Health, Topolová 748, 250 67, Klecany, Czech Republic; c Department of Global Public Health, Karolinska Institutet, Stockholm, Sweden; d Department of Psychiatry II, Ulm University and BKH Günzburg, Lindenallee 2, 89312, Günzburg, Germany; e Department Psychiatry A, Razi Hospital La Manouba, Rue Des Oranges, 2010, Manouba, Tunisia; f Faculty of Medicine of Tunis, University of Tunis El Manar, R534+F9H, Rue de la Faculte de Medecine, Tunis, Tunisia; g Institute of Psychiatry, Psychology, and Neuroscience, King's College London, United Kingdom; h Association for the Improvement of Mental Health Programmes, 20, Chemin Colladon, CH-1209, Geneva, Switzerland; i Department of Psychology, Faculty of Arts, Charles University, Celetná 20, 116 42, Prague, Czech Republic; j European Alliance Against Depression, Heinrich-Hoffman Strasse 10, Frankfurt, Germany; k Department of Psychiatry, Psychosomatic Medicine, and Psychotherapy, University Hospital, Goethe University, Heinrich-Hoffmann-Straße 10, 60528, Frankfurt am Main, Germany

**Keywords:** Stigma, Mental illness, Mental health, Attitudes, Discrimination, Global mental health

## Abstract

Current research evaluating the consequences of stigma towards people with mental illness is not nuanced in emphasizing the critical distinction between stigma as negative attitudes and discrimination as harmful behaviours that limit access to services, employment, and social inclusion. Understanding these distinctions is essential for designing targeted, evidence-based universal, targeted and indicated interventions to improve the quality of life and well-being. This review evaluates the evidence on the consequences of stigma towards people with mental illness. Using PRISMA guidelines, we analysed 448 studies (294 quantitative, 154 qualitative) investigating stigma's negative outcomes. Findings were categorized into health, service use, psychosocial, economic, and structural impacts. Although stigma is consistently associated with adverse outcomes across life domains, evidence of a causal link between negative attitudes and poorer outcomes for individuals with mental disorders remains limited. Furthermore, there is a striking scarcity of research from low- and middle-income countries, with significant regional gaps, and studies addressing structural stigma embedded in societal institutions are particularly rare. Efforts to combat stigma must distinguish between attitudes and behaviours, focusing on reducing discrimination while enhancing public mental health literacy and access to effective interventions. Tackling these challenges requires a comprehensive, evidence-informed approach to improving mental health outcomes for all.

## Introduction

Stigma related to mental illness is widely recognized as one of the most significant challenges in global mental health.^1^ Current evidence highlights pervasive negative attitudes toward individuals with mental health conditions,^2^ contributing to a cascade of negative consequences affecting individuals, families, communities, and even societal structures.^3^, ^4^, ^5^, ^6^ These consequences include under-detection, delayed help-seeking, treatment gaps,^7^, ^8^, ^9^, ^10^ perpetuation of healthcare disparities, poor access to treatment, and higher mortality rates among people with mental illness (PWMI).^7^^,^^11^, ^12^, ^13^ Despite efforts to assess the consequences of mental illness stigma,^14^ the causal pathways between negative attitudes and these outcomes remain poorly understood, partly due to challenges in definitions and conceptual clarity.

Stigma toward individuals with mental illness can be categorized into public, or self-stigma. Public stigma refers to the negative attitudes that society directs toward individuals with mental health conditions whereas self-stigma occurs when individuals internalize these societal stereotypes, leading to diminished self-esteem and reduced willingness to seek help.^15^^,^^16^

The modern concept of stigma emerged in 1963 when Goffman operationalized it as a distinctive mark given by society to an individual, signifying an ‘attribute, trait or disease that leads to any form of community sanction’.^17^^,^^18^ In this framework, stigma is distinct from discrimination: stigma represents a societal label laden with negative connotations, whereas discrimination involves actions that negatively impact those who bear this label. At the turn of the millennium, Link and Phelan (2001) argued that the exercise of power and the enforcement of discrimination are necessary conditions for stigmatization to occur.^6^ This definition was later criticized, and it has been suggested that labelling, negative stereotyping, linguistic separation (“us” versus “them”), and power asymmetry must all be present for stigma to arise.^19^

Nowadays, although evolutionary causes of stigma are well understood,^20^ mental health stigma is widely considered as a problem of knowledge (lack of it or ignorance) that is associated with negative attitudes (negative stereotypes, prejudices) that in turn is associated with negative behaviour (negative discrimination).^21^ This prevailing definition implies that discrimination is a subset of stigma rather than a standalone issue, potentially leading to the hypothesis that changing public attitudes may effectively reduce discrimination and improve the lives of people with and without mental health conditions.

First, power asymmetries exist in all societies, making it overly simplistic to claim that only dominant groups can stigmatize. Stigmatization operates through complex mechanisms that are not solely dependent on structural power.^20^ For example, if only dominant groups could stigmatize, then racist views held by white individuals in apartheid-era South Africa would cease to be stigmatizing once white dominance ended—an empirically and ethically untenable conclusion. Second, defining discrimination both as a component of stigma and as one of its consequences results in a logical fallacy, as no phenomenon can simultaneously be its own cause and its own outcome.

Third, much research and interventions on mental health stigma have so far merged stigma and discrimination and focused on shifting public attitudes,^21^ with limited investigation into whether such changes empirically cause reduced discrimination or tangible benefits for PWMI, such as improved access to services, employment opportunities, or social inclusion. This disconnect is particularly problematic given that the most harmful consequences of stigma often stem from discriminatory behaviours, which systematically limit access to essential resources and opportunities.^1^

Globally, combating mental health stigma has been declared a priority, with substantial resources dedicated to anti-stigma programs aimed at improving public attitudes. However, these programs often measure success based on changes in expressed attitudes rather than reductions in stigma's negative correlates, such as discrimination or systemic inequalities. If the underlying assumption—that reducing stigma will mitigate negative outcomes—is flawed or overly simplistic, such interventions may fail to address the most pressing challenges faced by PWMI.

In this review, we aim to map and analyse evidence on the negative consequences of mental health stigma, focusing on the causal pathways linking the label of mental illness to adverse outcomes across all domains of life, including health, service use, psychosocial, economic, and structural domains. By distinguishing between attitudes and discrimination as a problem of behaviours, we seek to provide a foundation for interventions that can improve mental health outcomes for all.

## Methods

### Search strategy and selection criteria

We followed established guidelines on conducting scoping reviews.^22^ To identify relevant studies, we applied a broad search strategy to the following scientific databases: Web of Knowledge (WoK), Ovid (including Global Health, HMIC, Medline, PsycINFO) and Cochrane Library, the first search was done on January 15th, 2020, an update of the search was done on April 23rd, 2024, and another on March 19th 2025. In our initial search strategy, we did not apply any time limit, and we used a combination of keywords related to ‘stigma’ with other mental health and consequences related keywords. For the update only papers published between 2020 and 2024 were included. To identify negative consequences, we used terms such as impacts, discrimination, problems, difficulties, ramifications, influences, effects, inequities, injustices, issues, etc. We provide the full search strategy in the Appendix.

We screened data according to the inclusion and exclusion criteria provided in Table 1. We included studies on negative consequences or correlates of stigma (henceforth ‘stigma related outcomes’) on PWMI, and we broadly operationalized a negative stigma related outcome as any negative outcome across any dimension of human life. We did not systematically analyse studies looking at associations between attitudes and intended behaviour, since we understand intended behaviour as an expression of social distance rather than as actual behaviour.

**Table 1:** Selection criteria.

Inclusion	Exclusion
Empirical data Qualitative Quantitative All designs Participants have mental disorder diagnosis (ICD-10)^23^ assessed by any criteria: Schizophrenia spectrum disorder (ICD)-10 F20–F29) Mood disorders (ICD F30–F39) Anxiety and other nonpsychotic mental disorders (ICD F40–F48) Personality disorders (ICD-10 F60–F69) Behavioural and emotional disorders occurring in childhood (ICD-10 F90–F98) Stigma assessed & related to mental illness by any tool (e.g., self-stigma, perceived stigma, public stigma towards people with mental illness) Negative stigma related outcome assessed for people with mental illness English, French, Spanish, German, Russian	Opinion papers Case studies Editorials Commentaries Conference abstracts Stigma attached to completed suicides Substance use disorders (ICD-10 F10–F19), organic mental disorders (e.g., dementia) (F00–F09), Eating disorders (ICD-10:F5) Intellectual disabilities (ICD-10 F70–F79), pervasive and specific developmental disorders (ICD-10 F80–F89) General populations Stigma related to treatment of mental disorders Studies on intended behaviour Consequences for family or caregivers

### Data extraction

Due to the large number of records, we split the data set in half and two pairs of researchers independently screened titles and abstracts of each half (AK, AA, and PW, ZG). Disagreements at this stage were resolved via discussion until consensus was reached amongst pairs. Next, two researchers (AA, ZG) independently screened full texts, each 50% (959 full texts split roughly in half) of the records. All disagreements were discussed and reviewed for a second time; further disagreements were resolved with a third investigator (PW or AK).

Two researchers (AA, ZG) extracted relevant study characteristics in Airtable, an online spreadsheet database service. Data extraction included details on author(s’) name(s), title, year of publication, geographical location (according to the United Nations geoscheme), sample size and diagnosis, specific findings related to consequences of stigma and scales used to measure them, as well as domain categorization of studies (see Table 1 in Appendix). For the update of the search in both 2024 and in 2025 the same method was used with two researchers updating the screening (ZG, HT).

### Data analysis

First, quantitative data was analysed to provide a global overview of the existing knowledge via descriptive summaries of the results. Data provided in the results sections of qualitative studies were open-coded.^24^ After examining the results of included studies, we assigned a label to each of the studies based on the type of identified stigma related outcome.

Next, we used a bottom-up approach to categorise labels into larger domains of stigma related outcome. The domains were as follows: health, service use, psychosocial, economic and structural outcomes. Health outcomes refer to outcomes related to the mental or somatic health of an individual (e.g., symptom severity, somatic illness or hospitalisation frequencies). Service use outcomes include behaviours such as treatment adherence or help seeking. Psychosocial outcomes include outcomes related to psychological and social aspects such as self-esteem, quality of life, social capital, etc. Economic outcomes refer to topics such as financial strain and housing. Finally structural outcomes refer to all non-individual level outcomes such as systematic and structural stigma.

In accordance with systematic scoping review guidelines,^22^ quality assessment was not conducted, since our aim was to provide an overview of evidence and to identify gaps. Furthermore, given the wide heterogeneity of eligible study designs, and the descriptive aims of the review, a formal assessment of publication bias and statistical heterogeneity was not feasible or appropriate. Instead, we categorised studies based on design and methodology to understand the strength of evidence. We categorically assigned quantitative studies into three groups: studies with a simple cross-sectional design identifying correlational associations (A) studies with cross-sectional design employing more complex statistical methods such as path analysis or structural equation modelling (B)^25^ and studies with longitudinal design (C). Finally, we sorted the data by labels and domains and by the strength of evidence with respect to a region of origin. Qualitative studies were treated as primary data and analysed inductively using open coding.^24^ Following the principles of thematic synthesis,^26^ codes were grouped into descriptive and analytical themes. Although based on second-order constructs, the data were approached as if they were primary qualitative data to enable cross-study interpretation and theme development.

## Results

**Fig. 1:**
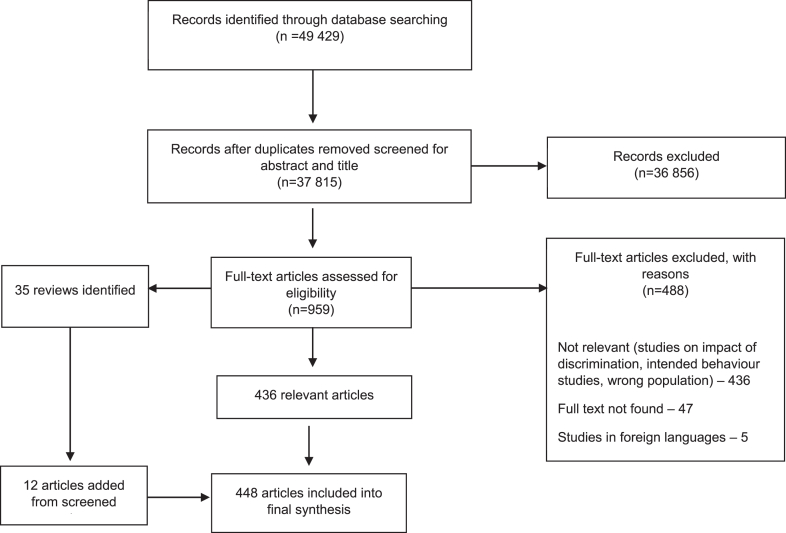
*PRISMA 2009 flow diagram*.^27^

Following deduplication, title and abstract screening resulted in 959 eligible articles for the full-text screening (Fig. 1). Most studies (n = 488) excluded at the full text stage did not meet our inclusion criteria for methodological requirements, such as vignette and intended behaviour studies. In total, 448 studies were included in this review (see Table 1 in Appendix for all included studies).

**Fig. 2:**
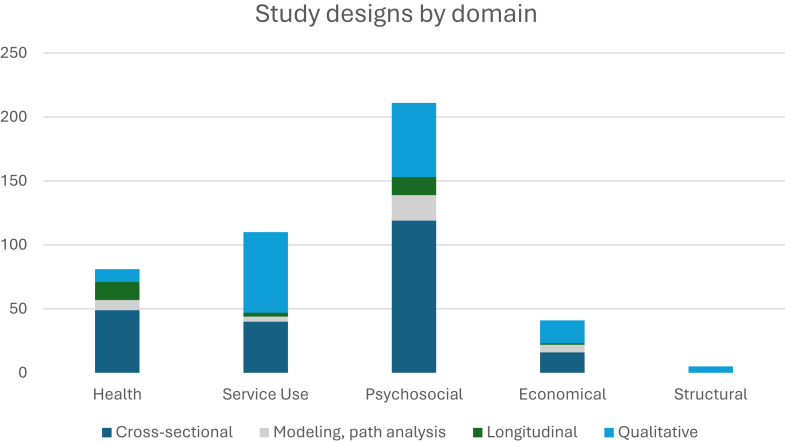
*Study designs by domain*.

Most identified quantitative studies used simple cross-sectional design identifying correlation or association of stigma with outcomes of interest (n = 224) followed by cross-sectional studies that employed complex analysis of data like structural equation modelling (n = 38) and longitudinal studies (n = 32). The remaining (n = 32) were descriptive studies on stigma consequences related to help-seeking or adherence to treatment (not included in Table 2). There were altogether 154 qualitative studies. Research from North America, Northern and Western Europe and Eastern Asia constitute most of the strongest evidence on stigma related outcomes, whereas some regions such as the Caribbean, African regions, Central Asia, Central America and Oceania were underrepresented. Fig. 2 shows the study distribution by domain. In light of the wide heterogeneity of study designs (467 studies, including both quantitative and qualitative work) and the descriptive aims of the review, a formal assessment of publication bias and statistical heterogeneity was not conducted. As shown in Table 2, most studies were conducted in high-income countries (HICs), particularly Northern America (142 studies), Northern Europe (48), and Western Europe (48). These regions also contributed most of the longitudinal and complex study designs. In contrast, low- and middle-income countries (LMICs) are vastly underrepresented. Several regions, including Central America and Asia, had no studies at all.

**Table 2:** Distribution of studies by region and level of evidence.

Region	Cross-sectional design	Cross-sectional design with complex analysis^a^	Longitudinal design	Qualitative design	Total
Eastern Africa	4	–	–	5	9
Western Africa	7	–	–	3	10
South America	6	–	1	2	9
Northern Africa	1	–	–	–	1
Northern America	59	11	13	59	142
Eastern Asia	27	11	2	6	46
South-eastern Asia	2	–	–	5	7
Southern Asia	13	1	–	9	23
Western Asia	12	2	–	10	24
Australia and New Zealand	5	1	–	16	22
Eastern Europe	20		–	1	21
Southern Europe	14	5	–	2	21
Northern Europe	23	2	4	19	48
Western Europe	18	3	12	9	42
International	13	1	–	3	17
Southern Africa	1	–	–	4	5
Caribbean	–	–	–	–	
Central America	–	–	–	–	
Central Asia	–	–	–	–	
Oceania	–	–	–	–	
Eastern Africa and Western Africa	–	–	–	1	1

Included one experimental study.^28^

a e.g., Path Analysis or Structural Equation Modelling.

### Health domain outcomes

Stigma is widely shown as a correlate of to be associated with the deterioration of mental health status. In a cohort study of 290 PWMI, Ilic and colleagues provided strong evidence that stigma experiences can lead to increased severity of psychiatric symptoms, lower self-esteem and quality of life.^29^ A survival analysis by Rüsch et al. showed that young people at risk for psychosis reporting stigma as a stressor were at higher risk of transitioning to schizophrenia.^30^ This is also reflected in a study where stigma related stress was associated with poorer recovery for PWMI at 2 year follow-up.^31^

Other longitudinal data suggest that stigma can be associated with higher depressive,^32^, ^33^, ^34^ and psychotic symptoms.^32^ Similarly, higher levels of self-stigma were significantly associated with increased positive symptoms and lower functioning after 12 months,^35^ and psychiatric hospitalisation after 6 months.^36^ Additionally, reduction in stigma scores during the follow-up period was associated with reduced depressive and anxiety symptoms and improvements towards clinical recovery.^37^ It is possible, however, that symptom severity can be both a predictor and an outcome in relation to self-stigma.^38^ Conversely, some studies found no evidence supporting stigma as a predictor of depressive symptoms at 3 months^39^ and 1 year follow-up^40^ or depression relapses at 2-year follow-up.^41^

Results related to impact of stigma on suicidality for PWMI are mixed. In participants at risk of psychosis, suicidality was predicted by increased stigma stress at 1-year follow-up.^42^ The same study did not find an association of suicidality with perceived stigma. Higher self-stigma predicted suicidal ideation after 2 years in people with psychiatric disabilities^43^ but there was no association found after 1 year in people with depression.^44^ Some evidence suggests that the link between stigma and suicidality is mediated by lower self-esteem,^45^ and hopelessness.^46^^,^^47^

There is a great amount of cross-sectional evidence suggesting that stigma is associated with worse depressive, anxiety, PTSD, substance abuse, ADHD or manic symptoms.^32^, ^33^, ^34^, ^35^, ^36^ However, the evidence linking stigma to the number of psychiatric admissions, duration of mental illness, and number of episodes is not globally generalisable as some studies report no significant association.^34^^,^^48^^,^^49^ A few studies examined the impact of stigma on physical health of PWMI. Some report that stigma is associated with self-reported poor physical health,^37^^,^^50^^,^^51^ and that stigma can mediate the effect of disability on perceived health.^52^

Multiple qualitative studies echoed outcomes found in the cross-sectional studies, such as increased experiences of psychotic and anxiety symptoms, lower mood and increased anxiety.^39^^,^^53^, ^54^, ^55^ Some qualitative studies report on treatment related consequences in healthcare settings, expressed as a lack of interest from health professionals, diagnostic overshadowing and perceived exclusion from PWMI in hospital and primary care settings, and ultimately further health complications.^56^^,^^57^

Longitudinal studies or cross-sectional studies with complex analysis on health consequences of stigma were scarce in the following regions: South America, Western and Eastern Africa, with South and West Asia having no such studies. See Table 2 in Appendix for complete distribution. No qualitative studies on health outcomes were found in East Asia and Western Africa.

Longitudinal studies show that stigma is associated with increased psychiatric symptoms, lower self-esteem and quality of life, and poorer recovery among PWMI. Cross-sectional studies link stigma to depressive, anxiety, PTSD, substance use, ADHD, and manic symptoms, as well as self-reported poor physical health. Qualitative studies report increased symptoms, treatment-related consequences, and healthcare exclusion. Evidence is limited in South America, Africa, and parts of Asia.

### Service use domain outcomes

Adherence, attitudes to treatment and even recovery orientation (such as hope for recovery) can be affected by stigma.^58^ Oexle and colleagues showed that higher self-stigma predicted less recovery orientation at baseline and at 1 year after controlling for symptom severity, age and gender, however these results did not hold after two years.^59^ The longitudinal evidence does provide some insight towards the evidence of causality or lack thereof in the observations of associated outcomes of the cross-sectional studies. Although some studies indicate a cross–sectional association of stigma and poor treatment adherence, both pharmaceutical and psychosocial,^58^^,^^60^, ^61^, ^62^ this was not found in a prospective study after 1 year follow-up in people who were prescribed antidepressants.^41^

In terms of treatment attitude, increase in perceived stigma after one year of follow-up predicted more negative attitudes toward psychotherapy,^63^ and stigma endorsement in people with depression predicted less help seeking at 6-months follow-up.^64^ It is possible, however, that intention to seek help is mediated by symptom severity,^65^ as well as by the level of self-efficacy.^66^ Another study showed that self-stigma mediates the link between perceived public stigma and help-seeking.^67^

Qualitative studies also provided insights into the consequences of stigma related to negative internal emotions experienced by PWMI such as shame, embarrassment, lowered self-esteem,^68^^,^^69^ and hopelessness,^70^ and often these consequences were linked to stigmatising friends and family.^68^ Qualitative data also suggests that stigma may have an impact on the process of recovery by inhibiting or slowing it down.^68^^,^^71^ Multiple qualitative studies presented reports from PWMI attributing poorer adherence to pharmaceutical treatment to stigma and internalised shame in relation to medication.^72^ This issue might be especially important in adolescents who refuse treatment due to stigma.^73^ Finally, several qualitative studies described reduced help-seeking as a consequence of stigma. This encompassed willingness and steps taken towards help-seeking,^74^ and shame attached to help-seeking.^75^

Longitudinal studies or cross-sectional studies with complex analysis on service use consequences of stigma were scarce in the following regions: South America, Western and Eastern Africa, with South East Asia having no such studies. See Table 3 in Appendix for complete distribution. No qualitative studies on service use consequences were found in: Southern and Western Africa, Central Asia.

Longitudinal, cross-sectional, and qualitative studies show that stigma in people with mental illness is associated with poorer psychiatric symptoms, lower self-esteem, reduced quality of life, slower recovery, and negative healthcare experiences, though findings for depression and suicidality are mixed. Evidence remains limited in many regions including South America, Africa, and Asia.

### Psychosocial domain outcomes

The relationship between stigma and multiple psychosocial variables such as self-esteem, quality of life, self-efficacy and others is complex.^76^, ^77^, ^78^, ^79^, ^80^, ^81^, ^82^, ^83^, ^84^, ^85^ Link et al. demonstrated that perceived stigma predicted lower self-esteem at 6 months follow-up after controlling for baseline levels of self-esteem, depressive symptoms and diagnosis in PWMI.^76^ Reduction in self-stigmatising beliefs over a follow-up period was associated with improvement in functioning and quality of life,^37^ and less emotional discomfort at six months,^77^ however other longitudinal studies failed to demonstrate significant association of self-stigma with quality of life.^78^^,^^79^ Factors like intensity of depressive symptoms, presence of social support, level of self-efficacy and empowerment can mediate perceived stigma's effect on quality of life.^72^^,^^81^ Similarly, variables such as hope, self-esteem or self-efficacy appear to be mediating the effect of self-stigma on quality of life,^82^ and well-being.^83^ Even poor sleep was linked to self-stigma, which could also affect quality of life.^84^ A decrease of stigma stress predicted improved well-being at 1 year follow-up,^85^ and an increase of self-stigma predicted higher levels of demoralisation at 1 year follow-up.^86^ Adverse effects of experienced stigma were shown also to have an effect on changes in symptoms and quality of life at 18 months follow up, but not self-esteem,^87^ and although cross-sectionally stereotype endorsement was related to emotional distress, there was no association in the longitudinal design.^88^

Longitudinal data indicate that self-stigma can impact social withdrawal of psychiatric patients.^89^ In a study where negative symptoms were controlled for, increased self-stigma predicted lower social functioning at 4 and 7 months follow-up in 39 people with schizophrenia, bipolar disorder and depression.^90^ Additionally, reduction in self-stigma positively impacts social adjustment, predicting changes in the community living skills of people with affective disorders over a 12-month period.^91^ In yet another study, it was shown that greater self-stigma can be associated with reduced social inclusion at 5-month follow-up which appeared to be mediated by hopelessness.^92^

There is no consistent quantitative evidence on the effect of stigma on education level, with mixed results showing association of stigma with lower education,^93^ higher education,^94^ or no association at all.^95^ Additionally, self-stigma was found to be associated with low social capital,^96^ lower relational satisfaction,^97^ and poor parenting experiences.^98^ Familial expressed emotions also were shown to be associated with self-stigma and experienced stigma.^99^

Qualitative studies mentioned stigma disrupting the education progress of PWMI, which was either linked to a lack of supportive accommodation from educational institutions or withdrawal from educational activities due to anticipated discrimination.^100^^,^^101^

Several qualitative studies reported PWMI experiencing avoidance from family members, friends and neighbours,^54^^,^^68^^,^^102^ feelings of shame by family members leading to exclusion,^102^, ^103^, ^104^ or being disowned by family.^105^^,^^106^ PWMI also report feeling limited in contributing to household and financial decisions.^71^^,^^102^ A loss of social contacts after disclosure of mental illness diagnosis was also reported as avoidance and, in some cases, shunning by friends. Relating to marriage, most studies described that family members of either spouse were reluctant towards a marriage due to fear of heritability of mental illness, or belief of increased violence of people with mental illness. This often resulted in diagnostic concealment when marrying.^54^^,^^70^^,^^107^ Conversely, disclosure of mental illness diagnosis may result in divorce.^108^ Stigma was also generally perceived to impact partner selection with studies reporting reduced prospects for dating for PWMI as well as reduced pursuit of dating by PWMI due to anticipated stigma and related fears.^54^^,^^69^^,^^71^ Quantitative evidence also report on stigma significantly increasing the odds of engaging in risky sexual behaviour.^109^

In qualitative studies, instances of abuse and harassment were most frequently defined as verbal and physical humiliation or ridicule, including sexual abuse and violence,^107^ and beating or throwing things at PWMI.^110^ Often the abusers of PWMI were people from their community such as neighbour's and family members.^54^ Physical restriction such as chaining and locking PWMI up is not uncommon.^54^^,^^99^ Forced abortion due to stigmatizing beliefs regarding heredity of mental illness was also reported as a consequence of stigma.^102^^,^^107^ Three studies described discrimination from law enforcement where participants felt discredited or disbelieved when reporting crime to law enforcement officers,^111^ or being suspected or targeted due to their mental illness.^112^

Finally, some qualitative studies described difficulties in gaining housing due to stigma. Loganathan et al. described a participant's experience where parents refused to give them a share of family property,^102^ similar experiences are described by Mathias et al.^113^ Other studies described landlords holding stigmatising attitudes which led to exclusion from property rentals or eviction.^56^^,^^113^

Studies with complex analysis or longitudinal design were scarce in South America, South and West Asia, Eastern Europe and Eastern Africa. See Table 4 in the Appendix for complete distribution. No qualitative studies on psychosocial consequences were found in South America and Western Africa; evidence was also scarce in Eastern Europe.

Longitudinal data indicate that stigma is associated with self-esteem, quality of life, social functioning, social withdrawal, and community living skills. Cross-sectional studies associate stigma with low social capital, poor relational satisfaction, and parenting difficulties. Qualitative studies describe disrupted education, social exclusion, family avoidance, relationship and marriage challenges, experiences of abuse, and barriers to housing. Evidence is limited in South America, Western Africa, Eastern Europe, and parts of Asia.

### Economic domain outcomes

In one longitudinal study, it was shown that greater self-stigma can be associated with reduced social inclusion and vocational activity at 5-month follow-up which appeared to be mediated by hopelessness.^92^ This echoes results by Chien et al., who showed that reduction in self-stigma positively predicted changes in the community living skills including work-life of people with affective disorders over a 12-month period.^91^

Most studies showed that stigma negatively impacted employment in multiple ways,^108^^,^^114^ one study found no relationship between stigma and employment status.^95^ In an experimental study fictitious candidates with reported mental illness history received significantly less callbacks from their job applications then candidates with physical illness history.^28^ However, in a different experiment, number of received callbacks did not differ between applications with year workplace inactivity due to depression and applications with unexplained reasons for such inactivity.^115^

In qualitative studies PWMI report less employment opportunities when disclosing mental health illness in job applications, and even not being able to secure a job as a result.^54^^,^^58^^,^^75^^,^^107^^,^^116^ Other studies also mentioned PWMI being fired once they disclosed mental illness.^117^^,^^118^ Within the workforce, self-stigma and experienced stigma were also shown to be associated with lower income and financial difficulties,^114^ as well as perceived public stigma linked to work and social limitations.^108^ PWMI report that stigma of employers has consequences on both, the importance of work they are assigned to and when seeking approval for medical leave or time off to visit mental healthcare professionals.^68^^,^^117^ The main impacts of co-worker stigma was bullying in the workplace and social distancing leading to sharing less exposure to collaboration and opportunities in their career.^105^ Finally, financial strain was often mentioned in qualitative studies as being directly related to a lack of employment opportunities.^54^^,^^89^^,^^102^

Stigma is associated with reduced vocational activity and social inclusion in some longitudinal studies. Cross-sectional studies indicate mixed findings with some negative effects on employment, though some report no association. Qualitative studies report reduced employment opportunities, job loss after disclosure, lower income, workplace bullying, and financial strain.

### Structural domain outcomes

Structural outcomes were found only in qualitative studies, with legal issues appearing first. Some studies attributed the existence of discriminatory laws a consequence of stigma in society. Such laws often either deemed PWMI as incapable of autonomous decision-making or used derogatory and/or inaccurate language.^116^^,^^117^^,^^119^ Furthermore, negative perceptions of PWMI (often related to perceived dangerousness) were used as justification for court-based custody decisions. In some studies, PWMI also perceived stigma as the reason for unfair treatment by child welfare services.^99^^,^^120^^,^^121^

Four studies explored stigma in the media, all of which described participants' belief that public stigma fuels negative imagery of PWMI.^53^^,^^56^^,^^104^^,^^116^ The impact of stigma on service user involvement was examined in two studies, both indicating that anticipated stigma and fear of not being heard or understood is a barrier to service user involvement in policy making and advocacy.^122^^,^^123^

Poor quality of mental healthcare services was also reported as an example of structural stigma, as well as low financing resulting in inadequate coverage of services and deficits in resources available, for example medication.^120^^,^^124^

Finally, one study from Northern Europe reported on the impacts of perceived and experienced stigma on accessing state benefits. Participants reported that the process in which state benefits for PWMI must be obtained was deeply stigmatizing, for example, the language used in the forms suggested that PWMI are not capable of deciding or living on their own.^125^

The studies within this domain came only from the following regions: Western, Eastern and Southern Europe, Western Africa and Southern Africa, Eastern Africa, Northern America and South-East Asia. For other regions, no studies were found.

Qualitative studies report stigma contributing to discriminatory laws, legal disadvantages, negative media representation, limited service-user involvement, poor-quality and underfunded mental healthcare, and barriers to accessing state benefits. Evidence is available only from Europe, Africa, North America, and South-East Asia.

## Discussion

This review analysed 448 studies on the negative outcomes of mental health stigma, categorized into health, service use, psychosocial, economic, and structural domains. Our findings highlight that while stigma is strongly associated with numerous adverse outcomes, the causal pathways linking stigma to outcomes remain poorly substantiated across all domains. With 56% (250 out of 448) of the reviewed studies being cross-sectional and only 7% (32 out of 448) and 8% (37 out of 448) longitudinal and path analysis with mixed findings, most evidence reflects correlations rather than causation, suggesting the need to critically consider strategies that primarily target negative attitudes toward people with mental health conditions to mitigate stigma's impact. The complex relationship between stigma and specific outcomes and broader domains require a more nuanced understanding to inform effective interventions.

Measuring stigma is inherently challenging, as it encompasses diverse constructs, including beliefs, attitudes, and cultural contexts.^126^ Much of the existing evidence base, particularly for structural outcomes relies on qualitative research (34%; i.e. 154 out of 448 of included studies were qualitative), which, while valuable for conceptualizing stigma, is limited in clarifying causal relationships.^127^ Similarly, most quantitative studies use cross-sectional designs, making it difficult to infer causality or directionality between stigma and domain outcomes. Mediating factors, such as symptom severity and self-efficacy, often complicate these relationships, as seen in associations between stigma and help-seeking or suicidality.^45^, ^46^, ^47^^,^^63^^,^^64^ Reverse causality further challenges interpretations, and while advanced statistical methods like path analysis exist, their use in stigma research remains limited.

Longitudinal and experimental studies, though scarce, provide stronger evidence for stigma's impact on health and psychosocial domain outcomes, including self-esteem, quality of life, and social withdrawal. However, research on the structural domain, operationalized as systemic discrimination embedded in institutions, is particularly underdeveloped, with only 13 qualitative studies identified and no quantitative research. This gap is critical, as structural stigma affects entire populations and requires greater investigation to understand its broad societal implications.

Little is also known about the interactions between internalized (micro) and systemic (macro) stigma and their compounded effects on people with mental illness (PWMI). Exploring these intersections could shed light on how stigma's consequences develop and how best to mitigate them.^128^ The lack of evidence from low- and middle-income countries (LMICs), where 85% of the global population resides, further limits the generalizability of current findings.^6^ Additionally, perspectives from ethnic minorities, different genders, sexualities, and diverse cultural contexts remain severely underrepresented, despite their importance in understanding stigma's varied impacts.^19^

Current evidence often attributes negative outcomes exclusively to stigma, although it is crucial to acknowledge that such outcomes may co-occur with, be mediated and/or be influenced by, intersecting factors such as unemployment or other forms of marginalization, rather than being solely caused by stigma.^129^^,^^130^ The impacts of stigma may compound over-time, such as self-labeling^81^ and public stigma perceptions,^131^ can exacerbate its effects, highlighting the need for longitudinal research to unravel these dynamics within and across all domains included in this study. Addressing these gaps is crucial for advancing evidence-based public health decision-making and optimizing resource allocation to improve the quality of life for PWMI globally.

This review's broad scope enabled an inclusive exploration of the consequences of mental illness stigma across diverse outcomes, without imposing pre-existing frameworks. This approach allowed for a comprehensive synthesis of findings across mental health conditions and study designs. However, several limitations must be acknowledged. As a scoping review, we did not systematically assess the methodological quality of included studies. We also excluded randomized controlled trials (RCTs), given the methodological difficulty of isolating stigma-specific effects from broader intervention outcomes. We also did not include studies on stigma related to completed suicides, substance use disorders, or eating disorders, which may involve distinct stigma processes; future research should explore the consequences of stigma in these important areas of mental health. Additionally, we did not differentiate between self-stigma, perceived stigma, and public stigma, which represents an important avenue for future research.

Additionally, categorizing stigma measures (e.g., self-stigma, public stigma) was challenging due to overlapping constructs, and grouping all mental illnesses under a single label may have underestimated stigma's impact on more severe conditions. Although most included studies focused on depression and affective disorders, few examined psychosis or other diagnoses, limiting both the interpretability and generalizability of the findings. While we excluded studies examining intended behaviours toward people with mental illness, this area represents a large portion of stigma related research. Therefore, a limitation of our results is a focus on measurable consequences of stigma therefore excluding intended behaviour.

Finally, while some longitudinal studies suggest associations between stigma and adverse outcomes, heterogeneity in study populations, control at baseline, follow-up durations, and analytic rigor limited our ability to draw strong causal inferences. Future research should prioritize greater diagnostic specificity, disaggregated analysis of subgroups by age, geographic diversity, particularly from low- and middle-income countries, and methodological consistency to clarify when and how stigma causally affects mental health outcomes.

Our findings reveal that, except for the negative effects of self-stigma, the evidence supporting stigma as a direct cause of social disadvantages remains limited. While stigma is often broadly linked to negative outcomes, the empirical foundation for claims of causality is still weak. Efforts to address stigma must distinguish between attitudes and behaviours, focusing on reducing discrimination while also enhancing public mental health literacy and access to effective interventions. Advancing stigma research requires a comprehensive, evidence-informed approach to identify explicit pathways through which stigma contributes to adverse outcomes and to disentangle its effects on health, service use, psychosocial, economical, and structural domains from other intersecting factors. This nuanced understanding is essential for developing targeted strategies to mitigate stigma's harmful effects and improve mental health outcomes for all.

## Contributors

Anna Kågström, Zoe Guerrero, Akmal Alikhan Aliev, and Petr Winkler designed the review, including the search strategy, inclusion and exclusion criteria, and data analysis. Anna Kågström, Zoe Guerrero, Akmal Alikhan Aliev, Hana Tomášková, and Petr Winkler reviewed the studies identified in the literature search and analyzed the data. Graham Thornicroft and Norman Sartorius played a key role in shaping the scope and design of the study and provided guidance on all aspects of the work as needed. Nicolas Rüsch and Uta Ouali contributed to discussing the findings, offering critical feedback, and assisting with their interpretation. Petr Winkler, Anna Kågström, and Zoe Guerrero led the manuscript preparation. Petr Winkler coordinated the study. All authors reviewed the manuscript drafts and provided critical intellectual input to enhance the paper; all authors read and approved the final version of the manuscript.

## Data sharing statement

This article is a review and does not involve the generation or analysis of new datasets. All data used were obtained from previously published studies, which are cited in the references.

## Declaration of generative AI and AI-assisted technologies in the writing process

During the preparation of this article, PW and AK used ChatGPT4 to improve the readability and language of the manuscript. Prompts such as “Is this clear, correct and comprehensible?” and alike were used. After using this tool, the author reviewed and edited the content as needed and take full responsibility for the content of the published article.

## Declaration of interests

The authors declare they have no competing interests that are relevant to the content of this article. Authors would like to further state the following: PW was a leader of the nation-wide anti-stigma project in Czechia between 2017 and 2025, and PW, AK and ZG were supported by this project at some point within the last 3 years. AA received payments in association with his role of a visiting lecturer at London School of Hygiene and Tropical Medicne, lecture on Public Mental Health in Eatern Europe and Central Asia. GT is supported by the National Institute for Health and Care Research (NIHR) Applied Research Collaboration South London (NIHR ARC South London) at King's College Hospital NHS Foundation Trust. The views expressed are those of the author(s) and not necessarily those of the NIHR or the Department of Health and Social Care. GT is also supported by the UK Medical Research Council (UKRI) for the Indigo Partnership (MR/R023697/1) awards. For the purpose of open access, the author has applied a Creative Commons Attribution (CC BY) licence (where permitted by UKRI, ‘Open Government Licence’ or ‘Creative Commons Attribution No-derivatives (CC BY-ND) licence’ may be stated instead) to any Author Accepted Author Manuscript version arising from this submission.’
