# Supplementary material for: Mental health stigma and its consequences: a systematic scoping review of pathways to discrimination and adverse outcomes
Source: eClinicalMedicine. 2025 Oct 23;89:103588. doi: 10.1016/j.eclinm.2025.103588 (PMC12593596; doi:10.1016/j.eclinm.2025.103588)
Supplement: Appendix [file mmc1.docx]

Appendix

Table of Contents

[Search strategy 1](#_Toc132964351)

[Data extraction table 2](#_Toc132964352)

[Types of stigma definitions 2](#_Toc132964353)

[Quantitative studies (full synthesis) 3](#_Toc132964354)

[Health consequences 4](#_Toc132964355)

[Cross-sectional data 8](#_Toc132964356)

[Longitudinal data 9](#_Toc132964357)

[Personal consequences 9](#_Toc132964358)

[Cross-sectional evidence 13](#_Toc132964359)

[Longitudinal evidence 14](#_Toc132964360)

[Economic consequences 15](#_Toc132964361)

[Psychosocial consequences 15](#_Toc132964362)

[Cross-sectional evidence 16](#_Toc132964363)

[Longitudinal evidence 17](#_Toc132964364)

[Qualitative studies (full synthesis) 20](#_Toc132964365)

[Organizational consequences 20](#_Toc132964366)

[Health consequences 21](#_Toc132964367)

[Personal consequences 21](#_Toc132964368)

[Psychosocial consequences 23](#_Toc132964369)

[Economic consequences 25](#_Toc132964370)

[Studies on intended behaviour 25](#_Toc132964371)

[Appendix references 26](#_Toc132964372)

# Search strategy

The following search strategy is an example used for the WoK database:

1.    TS=((mental* or psych*) NEAR/3 ("knowledge" or "literac*" or "educ*"))

2.    TS=(stigma* or stereotyp* or prejudice* or attitud*)

3.    1 or 2

4.    TS=(mental health OR mental illnes* or mental disorde* or mental diseas* or psychiatr* or common mental disorders or psychotic or psychosis or schizo* or bipolar disorder or anxiety or depression or manic depression or ocd or obsessive compulsive disorder or anorex* or bulim* or autism or post traumatic stress disorder or PTSD or post-traumatic stress disorder)

5.    TS=Discriminat*

6.    TS=(((stigma* or stereotype* or prejudice* or attitude*) NEAR/5 ("discrimination" or "consequenc*" or "impact*" or "problem*" or "implication*" or "effect*" or "outcome*" or "burden*" or "harm*" or "influence*" or "barrier*" or "role*" or "assoc*" or "correlat*" or "relationship*" or "famil*" or "friend*" or "organization*" or "institution*" or "work*" or "value*" or "norm*" or "law*" or "legal*" or "legislation*" or "polic*")))

7.    5 or 6

8.    3 and 4 and 7

Indexes=SCI-EXPANDED, SSCI, A&HCI, ESCI Timespan=All years

# Data extraction table: included studies

Table 1. Included studies

| **Name** | **Year** | **Title** | **Region** | | **Design** | | **Domain** |  |
| --- | --- | --- | --- | --- | --- | --- | --- | --- |
| Abayneh, S.; Lempp, H.; Alem, A.; Alemayehu, D.; Eshetu, T.; Lund, C.; Semrau, M.; Thornicroft, G.; Hanl | 2017 | Service User Involvement In Mental Health System Strengthening In A Rural African Setting: Qualitative Study | Eastern Africa | | Qualitative | | Structural |  |
| Ackerson, Barry J. | 2003 | Coping With The Dual Demands Of Severe Mental Illness And Parenting: The Parents' Perspective | Northern America | | Qualitative | | Psychosocial |  |
| Acosta, F. J.; Aguilar, E. J.; Cejas, M. R.; Gracia, R. | 2013 | Beliefs About Illness And Their Relationship With Hopelessness, Depression, Insight And Suicide Attempts In Schizophrenia | Southern Europe | | Cross-Sectional | | Health |  |
| Adeosun, Increase Ibukun; Adegbohun, Abosede Adekeji; Jeje, Oyetayo Oyewunmi; Adewumi, Tomilola Adejoke; | 2014 | Experiences Of Discrimination By People With Schizophrenia In Lagos, Nigeria | Western Africa | | Cross-Sectional | | Psychosocial |  |
| Adeponle, A.; Baduku, A.; Adelekan, M.; Suleiman, G.; Adeyemi, S | 2009 | Prospective Study Of Psychiatric Follow-Up Default And Medication Compliance After Discharge At A Psychiatric Hospital In Nigeria | Western Africa | | Cross-Sectional | | Service Use |  |
| Adewuya, A. O.; Owoeye, O. A.; Erinfolami, A. R.; Coker, A. O.; Ogun, O. C.; Okewole, A. O.; Dada, M. U.; Eze, C. N.; Bello-Mojeed, M. A.; Akindipe, T. O.; Olagunju, A. T.; Etim, E.; | 2009 | Prevalence And Correlates Of Poor Medication Adherence Amongst Psychiatric Outpatients In Southwestern Nigeria | Western Africa | | Cross-Sectional | | Service Use |  |
| Adewuya, Abiodun O.; Owoeye, Adekile O.; Erinfolami, A.; Ola, Bolanle A | 2010 | Correlates Of Self-Stigma Among Outpatients With Mental Illness In Lagos, Nigeria | Western Africa | | Cross-Sectional | | Health |  |
| Ahmed, A.; Stewart, D. E.; Teng, L.; Wahoush, O.; Gagnon, A. J. | 2018 | Experiences Of Immigrant New Mothers With Symptoms Of Depression | Northern America | | Qualitative | | Service Use |  |
| Alexova, A.; Kagstrom, A.; Winkler, P.; Kondratova, L.; Janouskova, M.; | 2019 | Correlates Of Internalized Stigma Levels In People With Psychosis In The Czech Republic | Eastern Europe | | Cross-Sectional | | Psychosocial |  |
| Alfayumi-Zeadna, Samira; Froimovici, Miron; Azbarga, Zoya; Grotto, Itmar; Daoud, Nihaya; | 2018 | Barriers To Postpartum Depression Treatment Among Indigenous Bedouin Women In Israel: A Focus Group Study | Western Asia | | Qualitative | | Service Use |  |
| Allan, E. B.; Najm, A. F.; Fernandes, H.; Allchin, B. | 2018 | Participatory Lived Experience Research: Barriers And Enablers For Social Inclusion For People With Psychosocial Disability, In Afghanistan? | Southern Asia | | Qualitative | | Psychosocial |  |
| Alonso, J.; Vilagut, G.; Adroher, N. D.; Chatterji, S.; He, Y.; Andrade, L. H.; Bromet, E.; Bruffaerts, R.; Fayyad, J.; Florescu, S.; De Girolamo, G.; Gureje, O.; Haro, J. M.; Hinkov, H.; Hu, C.; Iwata, N.; Lee, S.; Levinson, D.; Lepine, J. P.; Matschinger, H.; Medina-Mora, M. E.; O'Neill, S.; Ormel, J. H.; Posada-Villa, J. A.; Ismet Taib, N.; Xavier, M.; Kessler, R. C.; | 2013 | Disability Mediates The Impact Of Common Conditions On Perceived Health | International | | Cross-Sectional | | Psychosocial |  |
| Alonso, Jordi; Buron, Andrea; Rojas-Farreras, Sonia; De Graaf, Ron; Haro, Josep M.; De Girolamo, Giovanni; Bruffaerts, Ronny; Kovess, Viviane; Matschinger, Herbert; Vilagut, Gemma; | 2009 | Perceived Stigma Among Individuals With Common Mental Disorders | Western Europe, Southern Europe | | Cross-Sectional | | Psychosocial |  |
| Alonso, Jordi; Saha, Sukanta; Lim, Carmen C.; Aguilar-Gaxiola, Sergio; Al-Hamzawi, Ali; Benjet, Corina; Bromet, Evelyn J Et Al.; | 2018 | The Association Between Psychotic Experiences And Health-Related Quality Of Life: A Cross-National Analysis Based On World Mental Health Surveys | International | | Cross-Sectional | | Health |  |
| Andersson, L. M.; Schierenbeck, I.; Strumpher, J.; Krantz, G.; Topper, K.; Backman, G.; Ricks, E.; Van Rooyen, D.; | 2013 | Help-Seeking Behaviour, Barriers To Care And Experiences Of Care Among Persons With Depression In Eastern Cape, South Africa | Southern Africa | | Cross-Sectional | | Service Use |  |
| Andren, Katherine A.; Mckibbin, Christine L.; Wykes, Thomas L.; Lee, Aaron A.; Carrico, Catherine P.; Bourassa, Katelynn A | 2013 | Depression Treatment Among Rural Older Adults: Preferences And Factors Influencing Future Service Use | Northern America | | Qualitative | | Service Use |  |
| Andrzej Cechnicki, Matthias C. Angermeyer & Anna Bielańska | 2010 | Anticipated And Experienced Stigma Among People With Schizophrenia: Its Nature And Correlates | Eastern Europe | | Cross-Sectional | | Psychosocial |  |
| Arnaez, J. M.; Krendl, A. C.; Mccormick, B. P.; Chen, Z. X.; Chomistek, A. K.; | 2019 | The Association Of Depression Stigma With Barriers To Seeking Mental Health Care: A Cross-Sectional Analysis | Northern America | | Cross-Sectional | | Service Use |  |
| Assefa, D.; Shibre, T.; Asher, L.; Fekadu, A.; | 2012 | Internalized Stigma Among Patients With Schizophrenia In Ethiopia: A Cross-Sectional Facility-Based Study | Eastern Africa | | Cross-Sectional | | Health |  |
| Augsberger, A.; Yeung, A.; Dougher, M.; Hahm, H. C.; | 2015 | Factors Influencing The Underutilization Of Mental Health Services Among Asian American Women With A History Of Depression And Suicide | Northern America | | Qualitative | | Service Use |  |
| Aukst-Margetic, B.; Jaksic, N.; Boricevic Marsanic, V.; Jakovljevic, M.; | 2013 | Harm Avoidance Moderates The Relationship Between Internalized Stigma And Depressive Symptoms In Patients With Schizophrenia | Southern Europe | | Cross-Sectional | | Health |  |
| Aydemir, O.; Akkaya, C | 2011 | Association Of Social Anxiety With Stigmatisation And Low Self-Esteem In Remitted Bipolar Patients | Western Asia | | Complex Cross-Sectional | | Psychosocial |  |
| Baert, S.; De Visschere, S.; Schoors, K.; Vandenberghe, D.; Omey, E.; | 2016 | First Depressed, Then Discriminated Against? | Western Europe | | Complex Cross-Sectional | | Economical |  |
| Bahm, Allison, | 2009 | Interlocking Oppressions: The Effect Of A Comorbid Physical Disability On Perceived Stigma And Discrimination Among Mental Health Consumers In Canada | Northern America | | Cross-Sectional | | Health |  |
| Baldwin, M. L.; Marcus, S. C.; | 2006 | Perceived And Measured Stigma Among Workers With Serious Mental Illness | Northern America | | Cross-Sectional | | Economical |  |
| Barney, L. J.; Griffiths, K. M.; Christensen, H.; Jorm, A. F. | 2009 | Exploring The Nature Of Stigmatising Beliefs About Depression And Help-Seeking: Implications For Reducing Stigma | Australia And New Zealand | | Qualitative | | Service Use |  |
| Becker, Anne E.; Arrindell, Adrienne Hadley; Perloe, Alexandra; Fay, Kristen; Striegel-Moore, Ruth H.; | 2010 | A Qualitative Study Of Perceived Social Barriers To Care For Eating Disorders: Perspectives From Ethnically Diverse Health Care Consumers | Northern America | | Qualitative | | Service Use |  |
| Belloch, A.; Del Valle, G.; Morillo, C.; Carrio, C.; Cabedo, E.; | 2009 | To Seek Advice Or Not To Seek Advice About The Problem: The Help-Seeking Dilemma For Obsessive-Compulsive Disorder | Southern Europe | | Cross-Sectional | | Service Use |  |
| Ben-Zeev, Dror; Frounfelker, Rochelle; Morris, Scott B.; Corrigan, Patrick W.; | 2012 | Predictors Of Self-Stigma In Schizophrenia: New Insights Using Mobile Technologies | Northern America | | Longitudinal | | Health |  |
| Berge, M.; Ranney, M.; | 2005 | Self-Esteem And Stigma Among Persons With Schizophrenia: Implications For Mental Health | Northern America | | Cross-Sectional | | Psychosocial |  |
| Berry, C.; Greenwood, K.; | 2018 | Direct And Indirect Associations Between Dysfunctional Attitudes, Self-Stigma, Hopefulness And Social Inclusion In Young People Experiencing Psychosis | Northern Europe | | Longitudinal | | Economical |  |
| Berzins, K. M.; Petch, A.; Atkinson, J. | 2003 | Prevalence And Experience Of Harassment Of People With Mental Health Problems Living In The Community | Northern Europe | | Qualitative | | Psychosocial |  |
| Bjorkman, T.; Svensson, B.; Lundberg, B.; | 2007 | Experiences Of Stigma Among People With Severe Mental Illness. Reliability, Acceptability And Construct Validity Of The Swedish Versions Of Two Stigma Scales Measuring Devaluation/Discrimination And Rejection Experiences | Northern Europe | | Cross-Sectional | | Psychosocial |  |
| Blais, Rebecca K.; Tsai, Jack; Southwick, Steven M.; Pietrzak, Robert H.; | 2015 | Barriers And Facilitators Related To Mental Health Care Use Among Older Veterans In The United States | Northern America | | Cross-Sectional | | Service Use |  |
| Blankertz, L.; | 2001 | Cognitive Components Of Self Esteem For Individuals With Severe Mental Illness | Northern America | | Cross-Sectional | | Psychosocial |  |
| Blignault, I.; Ponzio, V.; Rong, Y.; Eisenbruch, M.; | 2008 | A Qualitative Study Of Barriers To Mental Health Services Utilisation Among Migrants From Mainland China In South-East Sydney | Australia And New Zealand | | Qualitative | | Service Use |  |
| Bloch Raz, Shulamit, Igra, Libby, Lavi-Rotenberg, Adi, Pijnenborg, Gerdina Hendrika Maria, Van Der Meer, Lisette, Hasson-Ohayon, Ilanit, Aldao, A., Nolen-Hoeksema, S., Aldao, Beck, A. T., Steer, R. A., Brown, G., Beck, Berking, M | 2024 | Emotion Regulation And Self-Stigma Among People With Schizophrenia: The Mediating Role Of Depression. | Northern Europe | | Cross-Sectional | | Health |  |
| Bolton, Jim; | 2018 | We've Got Another One For You!' : Liaison Psychiatry's Experience Of Stigma Towards Patients With Mental Illness And Mental Health Professionals | Northern Europe | | Cross-Sectional | | Health |  |
| Bos, A. E.; Kanner, D.; Muris, P.; Janssen, B.; Mayer, B.; | 2009 | Mental Illness Stigma And Disclosure: Consequences Of Coming Out Of The Closet | Western Europe | | Cross-Sectional | | Psychosocial |  |
| Boucher, M. E.; Groleau, D.; Whitley, R | 2016 | Recovery And Severe Mental Illness: The Role Of Romantic Relationships, Intimacy, And Sexuality. | Northern America | | Qualitative | | Psychosocial |  |
| Boyd, Candice; Francis, Kristy; Aisbett, Damon; Newnham, Krystal; Sewell, Jessica; Dawes, Graham; Nur | 2007 | Australian Rural Adolescents' Experiences Of Accessing Psychological Help For A Mental Health Problem | Australia And New Zealand | | Qualitative | | Service Use |  |
| Boyd, J. E.; Hayward, H.; Bassett, E. D.; Hoff, R. | 2016 | Internalized Stigma Of Mental Illness And Depressive And Psychotic Symptoms In Homeless Veterans Over 6 Months | Northern America | | Longitudinal | | Health |  |
| Brain, C.; Sameby, B.; Allerby, K.; Quinlan, P.; Joas, E.; Lindstrom, E.; Burns, T.; Waern, M.; | 2014 | Stigma, Discrimination And Medication Adherence In Schizophrenia: Results From The Swedish COAST Study | Northern Europe | | Longitudinal | | Health |  |
| Brenman, N. F.; Luitel, N. P.; Mall, S.; Jordans, M. J.; | 2014 | Demand And Access To Mental Health Services: A Qualitative Formative Study In Nepal | Southern Asia | | Qualitative | | Service Use |  |
| Brondani, M. A.; Alan, R.; Donnelly, L. | 2017 | Stigma Of Addiction And Mental Illness In Healthcare: The Case Of Patients' Experiences In Dental Settings | Northern America | | Qualitative | | Service Use |  |
| Brophy, Lisa M.; Roper, Catherine E.; Hamilton, Bridget E.; Tellez, Juan Jose; Mcsherry, Bernadette M. | 2016 | Consumers And Carer Perspectives On Poor Practice And The Use Of Seclusion And Restraint In Mental Health Settings: Results From Australian Focus Groups Qualitative | Australia And New Zealand | | Qualitative | | Psychosocial |  |
| Brouwers, E. P.; Mathijssen, J.; Van Bortel, T.; Knifton, L.; Wahlbeck, K.; Van Audenhove, C.; Et Al | 2016 | Discrimination In The Workplace, Reported By People With Major Depressive Disorder: A Cross-Sectional Study In 35 Countries. | International | | Cross-Sectional | | Economical |  |
| Brown, Charlotte; Conner, Kyaien O.; Copeland, Valire Carr; Grote, Nancy; Beach, Scott; Battista, Deena; Reynolds, Charles F., III; | 2010 | Depression Stigma, Race, And Treatment Seeking Behavior And Attitudes | Northern America | | Cross-Sectional | | Service Use |  |
| Brown, Ellie; Topping, Annie; Cheston, Richard; | 2019 | What Are The Barriers To Accessing Psychological Therapy In Qatar: A Concept Mapping Study | Western Asia | | Qualitative | | Psychosocial |  |
| Brown, M. C.; Creel, A. H.; Engel, C. C.; Herrell, R. K.; Hoge, C. W. | 2011 | Factors Associated With Interest In Receiving Help For Mental Health Problems In Combat Veterans Returning From Deployment To Iraq | Northern America | | Cross-Sectional | | Service Use |  |
| Bryant, K.; Greer-Williams, N.; Willis, N.; Hartwig, M.; | 2013 | Barriers To Diagnosis And Treatment Of Depression: Voices From A Rural African-American Faith Community | Northern America | | Qualitative | | Service Use |  |
| Buizza, Chiara; Schulze, Beate; Bertocchi, Elena; Rossi, Giuseppe; Ghilardi, Alberto; Pioli, Rosaria; | 2007 | The Stigma Of Schizophrenia From Patients' And Relatives' View: A Pilot Study In An Italian Rehabilitation Residential Care Unit | Southern Europe | | Qualitative | | Health |  |
| Burke, Eilish; Wood, Lisa; Zabel, Elisabeth; Clark, Alexandra; Morrison, Anthony P.; | 2016 | Experiences Of Stigma In Psychosis: A Qualitative Analysis Of Service Users' Perspectives | Northern Europe | | Qualitative | | Health |  |
| Burtea, V.; Mosoiu, C. | 2008 | Stigmatization Of People With Schizophrenia In Clinical Settings | Eastern Europe | | Cross-Sectional | | Psychosocial |  |
| Busby Grant, J.; Bruce, C.; Batterham, P. | 2019 | Predictors Of Personal, Perceived And Self-Stigma Towards Anxiety And Depression | Australia And New Zealand | | Cross-Sectional | | Health |  |
| Byatt, N.; Biebel, K.; Friedman, L.; Debordes-Jackson, G.; Ziedonis, D.; Pbert, L.; | 2013 | Patient's Views On Depression Care In Obstetric Settings: How Do They Compare To The Views Of Perinatal Health Care Professionals? | Northern America | | Qualitative | | Service Use |  |
| Bye, A.; Shawe, J.; Bick, D.; Easter, A.; Kash-Macdonald, M.; Micali, | 2018 | Barriers To Identifying Eating Disorders In Pregnancy And In The Postnatal Period: A Qualitative Approach | Northern Europe | | Qualitative | | Service Use |  |
| Byrow, Y.; Pajak, R.; Mcmahon, T.; Rajouria, A.; Nickerson, A.; | 2019 | Barriers To Mental Health Help-Seeking Amongst Refugee Men | Australia And New Zealand | | Complex Cross-Sectional | | Psychosocial |  |
| Cai, C.; Yu, L.; | 2017 | Quality Of Life In Patients With Schizophrenia In China: Relationships Among Demographic Characteristics, Psychosocial Variables, And Symptom Severity | Eastern Asia | | Cross-Sectional | | Health |  |
| Campbell, D. G.; Bonner, L. M.; Bolkan, C. R.; Lanto, A. B.; Zivin, K.; Waltz, T. J.; Klap, R.; Rubenstein, L. V.; Chaney, E. F.; | 2016 | Stigma Predicts Treatment Preferences And Care Engagement Among Veterans Affairs Primary Care Patients With Depression | Northern America | | Longitudinal | | Service Use |  |
| Campbell, Rosalyn Denise; Mowbray, Orion | 2016 | The Stigma Of Depression: Black American Experiences | Northern America | | Qualitative | | Service Use |  |
| Campellone, T. R.; Caponigro, J. M.; Kring, A. M.; | 2014 | The Power To Resist: The Relationship Between Power, Stigma, And Negative Symptoms In Schizophrenia | Northern America | | Cross-Sectional | | Psychosocial |  |
| Capar, Meltem; Kavak, Funda; | 2018 | Effect Of Internalized Stigma On Functional Recovery In Patients With Schizophrenia | Western Asia | | Cross-Sectional | | Psychosocial |  |
| Caqueo-Urizar, Alejandra; Urzua, Alfonso; Habib, Julia; Loundou, Anderson; Boucekine, Mohamed; Boyer, Laurent; Fond, Guillaume | 2019 | Relationships Between Social Stigma, Stigma Experience And Self-Stigma And Impaired Quality Of Life In Schizophrenia Across Three Latin-American Countries | South America | | Cross-Sectional | | Psychosocial |  |
| Cárcamo Guzmán, K., Cofré Lira, I., Flores Oyarzo, G., Lagos Arriagada, D., Oñate Vidal, N., & Grandón Fernández, P. | 2019 | Mental Health Care For Persons With A Severe Psychiatric Diagnosis And Their Recovery | South America | | Qualitative | | Service Use |  |
| Cavelti, M.; Kvrgic, S.; Beck, E. M.; Rusch, N.; Vauth, R.; | 2012 | Self-Stigma And Its Relationship With Insight, Demoralization, And Clinical Outcome Among People With Schizophrenia Spectrum Disorders | Western Europe | | Complex Cross-Sectional | | Psychosocial |  |
| Cavelti, M.; Rusch, N.; Vauth, R | 2014 | Is Living With Psychosis Demoralizing? Insight, Self-Stigma, And Clinical Outcome Among People With Schizophrenia Across 1 Year | Western Europe | | Longitudinal | | Psychosocial |  |
| Cerit, Cem; Filizer, Arzu; Tural, Umit; Tufan, Ali Evren; | 2012 | Stigma: A Core Factor On Predicting Functionality In Bipolar Disorder | Western Asia | | Cross-Sectional | | Health |  |
| Chan, K. K. S.; Fung, W. T. W.; | 2019 | The Impact Of Experienced Discrimination And Self-Stigma On Sleep And Health-Related Quality Of Life Among Individuals With Mental Disorders In Hong Kong | Eastern Asia | | Complex Cross-Sectional | | Psychosocial |  |
| Chan, K. K. S.; Lam, C. B.; | 2018 | The Impact Of Familial Expressed Emotion On Clinical And Personal Recovery Among Patients With Psychiatric Disorders: The Mediating Roles Of Self-Stigma Content And Process | Eastern Asia | | Complex Cross-Sectional | | Health |  |
| Chan, K. K. S.; Mak, W. W. S.; | 2014 | The Mediating Role Of Self-Stigma And Unmet Needs On The Recovery Of People With Schizophrenia Living In The Community | Eastern Asia | | Cross-Sectional | | Psychosocial |  |
| Chan, Randolph C.; Mak, Winnie W.; | 2016 | Common Sense Model Of Mental Illness: Understanding The Impact Of Cognitive And Emotional Representations Of Mental Illness On Recovery Through The Mediation Of Self-Stigma | Eastern Asia | | Complex Cross-Sectional | | Health |  |
| Chan, Sonia Ying Yin; Ho, Grace W.; Bressington, Daniel; | 2018 | Experiences Of Self-Stigmatization And Parenting In Chinese Mothers With Severe Mental Illness | Eastern Asia | | Qualitative | | Psychosocial |  |
| Chavira, Denise A.; Bantados, Brenda; Rapp, Amy; Firpo-Perretti, Yudelki M.; Escovar, Emily; Dixon, Louise; Drahota, Amy; Palinkas, Lawrence A.; | 2017 | Parent-Reported Stigma And Child Anxiety: A Mixed Methods Research Study | Northern America | | Qualitative | | Service Use |  |
| Chen, E. S.; Chang, W. C.; Hui, C. L.; Chan, S. K.; Lee, E. H.; Chen, E | 2016 | Self-Stigma And Affiliate Stigma In First-Episode Psychosis Patients And Their Caregivers | Eastern Asia | | Cross-Sectional | | Psychosocial |  |
| Chen, F. P.; Wu, H. C.; Huang, C. J.; | 2014 | Influences Of Attribution And Stigma On Working Relationships With Providers Practicing Western Psychiatry In The Taiwanese Context | Eastern Asia | | Cross-Sectional | | Service Use |  |
| Chen, H. T.; Coakley, E. H.; Cheal, K.; Maxwell, J.; Costantino, G.; Krahn, D. D.; Malgady, R. G.; Durai, U. N. B.; Quijano, L. M.; Zaman, S.; Miller, C. J.; Ware, J. H.; Chung, H.; Aoyama, C.; Van Stone, W. W.; Levkoff, S. E.; | 2006 | Satisfaction With Mental Health Services In Older Primary Care Patients | Northern America | | Cross-Sectional | | Health |  |
| Chen, Haide; Fang, Xiaoyi; Liu, Chaoying; Hu, Wei; Lan, Jing; Deng, Linyuan; | 2014 | Associations Among The Number Of Mental Health Problems, Stigma, And Seeking Help From Psychological Services: A Path Analysis Model Among Chinese Adolescents | Eastern Asia | | Complex Cross-Sectional | | Service Use |  |
| Chien, W. T.; Lam, C. K.; Ng, B. F | 2015 | Predictors Of Levels Of Functioning Among Chinese People With Severe Mental Illness: A 12-Month Prospective Cohort Study | Eastern Asia | | Longitudinal | | Psychosocial |  |
| Chien, Waitong; Yeung, F. K. K.; Chan, A. H. L.; | 2014 | Perceived Stigma Of Patients With Severe Mental Illness In Hong Kong: Relationships With Patients' Psychosocial Conditions And Attitudes Of Family Caregivers And Health Professionals | Eastern Asia | | Cross-Sectional | | Health |  |
| Chotkowska, K.; | 2014 | Experienced And Perceived Stigma Among People Suffering From Schizophrenia | Eastern Europe | | Cross-Sectional | | Psychosocial |  |
| Chronister, J.; Chou, C. C.; Liao, Hsinya | 2013 | The Role Of Stigma Coping And Social Support In Mediating The Effect Of Societal Stigma On Internalized Stigma, Mental Health Recovery, And Quality Of Life Among People With Serious Mental Illness | Northern America | | Complex Cross-Sectional | | Health |  |
| Chung, K. F.; Tse, S.; Lee, C. T.; Chan, W. M.; | 2019 | Changes In Stigma Experience Among Mental Health Service Users Over Time: A Qualitative Study With Focus Groups | Eastern Asia | | Qualitative | | Economical |  |
| Chung, L.; Pan, A. W.; Hsiung, P. C. | 2009 | Quality Of Life For Patients With Major Depression In Taiwan:A Model-Based Study Of Predictive Factors | Eastern Asia | | Complex Cross-Sectional | | Psychosocial |  |
| Cinculova, A.; Prasko, J.; Kamaradova, D.; Ociskova, M.; Latalova, K.; Vrbova, K.; Kubinek, R.; Mainerova, B.; Grambal, A.; Tichackova, A.; | 2017 | Adherence, Self-Stigma And Discontinuation Of Pharmacotherapy In Patients With Anxiety Disorders - Cross-Sectional Study | Eastern Europe | | Cross-Sectional | | Health |  |
| Clement, Sarah; Williams, Paul; Farrelly, Simone; Hatch, Stephani L.; Schauman, Oliver; Jeffery, Debra; Henderson, R.; Thornicroft, Graham | 2015 | Mental Health-Related Discrimination As A Predictor Of Low Engagement With Mental Health Services | Northern Europe | | Complex Cross-Sectional | | Service Use |  |
| Collins, P. Y.; Unger, H. Von; Armbrister, A.; | 2009 | Church Ladies, Good Girls, And Locas: Stigma And The Intersection Of Gender, Ethnicity, Mental Illness, And Sexuality In Relation To HIV Risk | Northern America | | Qualitative | | Psychosocial |  |
| Conner, K. O.; Copeland, V. C.; Grote, N. K.; Koeske, G.; Rosen, D.; Reynolds, C. F., 3rd; Brown, C.; | 2010 | Mental Health Treatment Seeking Among Older Adults With Depression: The Impact Of Stigma And Race | Northern America | | Cross-Sectional | | Service Use |  |
| Conner, K. O.; Lee, B.; Mayers, V.; Robinson, D.; Reynolds, C. F.; Albert, S.; Brown, C.; | 2010 | Attitudes And Beliefs About Mental Health Among African American Older Adults Suffering From Depression | Northern America | | Qualitative | | Service Use |  |
| Conrad, Margaret M.; Pacquiao, Dula F.; | 2010 | Manifestation, Attribution, And Coping With Depression Among Asian Indians From The Perspectives Of Health Care Practitioners | Northern America | | Qualitative | | Service Use |  |
| Corbiere, M.; Renard, M.; St-Arnaud, L.; Coutu, M. F.; Negrini, A.; Sauve, G.; Lecomte, T.; | 2015 | Union Perceptions Of Factors Related To The Return To Work Of Employees With Depression | Northern America | | Qualitative | | Economical |  |
| Corker, E.; Henderson, C.; Thornicroft, G | 2011 | The Viewpoint Discrimination Survey - The Extent Of Discrimination Faced By Mental Health Service Users In England | Northern Europe | | Cross-Sectional | | Psychosocial |  |
| Corker, Elizabeth A.; Beldie, Alina; Brain, Cecilia; Jakovljevic, Miro; Jarema, Marek; Karamustafalioglu, Oguz; Marksteiner, Josef; Mohr, Pavel; Prelipceanu, Dan; Vasilache, Anamaria; Waern, Margda; Sartorius, Norman; Thornicroft, Graham; | 2015 | Experience Of Stigma And Discrimination Reported By People Experiencing The First Episode Of Schizophrenia And Those With A First Episode Of Depression: The FEDORA Project | International | | Cross-Sectional | | Psychosocial |  |
| Corrigan, P. W., Watson, A. C., Heyrman, M. L., Warpinski, A., Gracia, G., Slopen, N., & Hall, L. L | 2005 | Structural Stigma In State Legislation | Northern America | | Qualitative | | Structural |  |
| Corrigan, P. W.; Bink, A. B.; Schmidt, A.; Jones, N.; Rusch, N. | 2016 | What Is The Impact Of Self-Stigma? Loss Of Self-Respect And The "Why Try" Effect | Northern America | | Cross-Sectional | | Psychosocial |  |
| Corrigan, P. W.; Powell, K. J.; Rusch, N.; | 2012 | How Does Stigma Affect Work In People With Serious Mental Illnesses? | Northern America | | Cross-Sectional | | Economical |  |
| Corrigan, P.; Thompson, V.; Lambert, D.; Sangster, Y.; Noel, J. G.; Campbell, J. | 2003 | Perceptions Of Discrimination Among Persons With Serious Mental Illness | Northern America | | Cross-Sectional | | Psychosocial |  |
| Crowe, Allison; Averett, Paige; Glass, J.; | 2016 | Mental Illness Stigma, Psychological Resilience, And Help Seeking: What Are The Relationships? | Northern America | | Qualitative | | Service Use |  |
| Crumb, Loni; Mingo, Taryne M.; Crowe, Allison; | 2019 | Get Over It And Move On: The Impact Of Mental Illness Stigma In Rural, Low-Income United States Populations | Northern America | | Qualitative | | Service Use |  |
| Cruwys, T.; Gunaseelan, S.; | 2016 | Depression Is Who I Am: Mental Illness Identity, Stigma And Wellbeing | International | | Cross-Sectional | | Health |  |
| Cullen, B. A.; Mojtabai, R.; Bordbar, E.; Everett, A.; Nugent, K. L.; Eaton, W. W.; | 2017 | Social Network, Recovery Attitudes And Internal Stigma Among Those With Serious Mental Illness | Northern America | | Cross-Sectional | | Psychosocial |  |
| Daumerie, N.; Bacle, S. V.; Giordana, J. Y.; Mannone, C. B.; Caria, A.; Roelandt, J. L.; | 2012 | Discrimination Perceived By People With A Diagnosis Of Schizophrenic Disorders. International Study Of Discrimination And Stigma Outcomes (INDIGO): French Results | Western Europe | | Cross-Sectional | | Economical |  |
| Davison, Jo; Zamperoni, Victoria; Stain, Helen J. | 2017 | Vulnerable Young People’s Experiences Of Child And Adolescent Mental Health Services | Northern Europe | | Qualitative | | Service Use |  |
| Demery, Rachel; Thirlaway, Kathryn; Mercer, Jenny; | 2012 | The Experiences Of University Students With A Mood Disorder | Northern Europe | | Qualitative | | Service Use |  |
| Denenny, D.; Thompson, E.; Pitts, S. C.; Dixon, L. B.; Schiffman, J.; | 2015 | Subthreshold Psychotic Symptom Distress, Self-Stigma, And Peer Social Support Among College Students With Mental Health Concerns | Northern America | | Cross-Sectional | | Health |  |
| Depla, Mfia; De Graaf, R.; Van Weeghel, J.; Heeren, T. J | 2005 | The Role Of Stigma In The Quality Of Life Of Older Adults With Severe Mental Illness | Western Europe | | Cross-Sectional | | Psychosocial |  |
| Dickerson, F. B.; Sommerville, J.; Origoni, A. E.; Ringel, N. B.; Parente, F.; | 2001 | Experiences Of Stigma Among Outpatients With Schizophrenia | Northern America | | Complex Cross-Sectional | | Economical |  |
| Dikec, G.; Uzunoglu, G.; Gumus, F.; | 2019 | Stigmatization Experiences Of Turkish Parents Of Patients Hospitalized In Child And Adolescent Psychiatric Clinic | Western Asia | | Qualitative | | Economical |  |
| Dimitropoulos, G.; Mccallum, L.; Colasanto, M.; Freeman, V. E.; Gadalla, T.; | 2016 | The Effects Of Stigma On Recovery Attitudes In People With Anorexia Nervosa In Intensive Treatment | Northern America | | Cross-Sectional | | Psychosocial |  |
| Dinos, S.; Stevens, S.; Serfaty, M.; Weich, S.; King, M.; | 2004 | Stigma: The Feelings And Experiences Of 46 People With Mental Illness | Northern Europe | | Qualitative | | Psychosocial |  |
| Dockery, L.; Jeffery, D.; Schauman, O.; Williams, P.; Farrelly, S.; Bonnington, O.; Gabbidon, J.; Lassman, F.; Szmukler, G.; Thornicroft, G.; Clement, S.; Group, Miriad Study; | 2015 | Stigma- And Non-Stigma-Related Treatment Barriers To Mental Healthcare Reported By Service Users And Caregivers | Northern Europe | | Cross-Sectional | | Service Use |  |
| Drapalski, A. L.; Lucksted, A.; Perrin, P. B.; Aakre, J. M.; Brown, C. H.; Deforge, B. R.; Boyd, J. E.; | 2013 | A Model Of Internalized Stigma And Its Effects On People With Mental Illness | Northern America | | Complex Cross-Sectional | | Psychosocial |  |
| Eapen, Valsamma; Ghibash, Rafia | 2014 | Help-Seeking For Mental Health Problems Of Children: Preferences And Attitudes In The United Arab Emirates | Western Asia | | Qualitative | | Service Use |  |
| Ebuenyi, I. D.; Guxens, M.; Ombati, E.; Bunders-Aelen, J. F. G.; Regeer, B. J.; | 2019 | Employability Of Persons With Mental Disability: Understanding Lived Experiences In Kenya | Eastern Africa | | Qualitative | | Economical |  |
| Ebuenyi, I. D.; Regeer, B. J.; Ndetei, D. M.; Bunders-Aelen, J. F. G.; Guxens | 2019 | Experienced And Anticipated Discrimination And Social Functioning In Persons With Mental Disabilities In Kenya: Implications For Employment | Eastern Africa | | Cross-Sectional | | Economical |  |
| Edwards, Elizabeth; Timmons, Stephen; | 2005 | A Qualitative Study Of Stigma Among Women Suffering Postnatal Illness | Northern Europe | | Qualitative | | Service Use |  |
| Egbe, C. O.; Brooke-Sumner, C.; Kathree, T.; Selohilwe, O.; Thornicroft, G.; Petersen, I.; | 2014 | Psychiatric Stigma And Discrimination In South Africa: Perspectives From Key Stakeholders | Southern Africa | | Qualitative | | Psychosocial |  |
| El-Badri, S.; Mellsop, G.; | 2007 | Stigma And Quality Of Life As Experienced By People With Mental Illness | International | | Cross-Sectional | | Psychosocial |  |
| Elkington, K. S.; Mckinnon, K.; Mann, C. G.; Collins, P. Y.; Leu, C. S.; Wainberg, M. L.; | 2010 | Perceived Mental Illness Stigma And HIV Risk Behaviors Among Adult Psychiatric Outpatients In Rio De Janeiro, Brazil | South America | | Cross-Sectional | | Psychosocial |  |
| Elkington, Katherine S.; Hackler, Dusty; Mckinnon, Karen; Borges, Cristiane; Wright, Eric R.; Wainberg, Milton L.; | 2012 | Perceived Mental Illness Stigma Among Youth In Psychiatric Outpatient Treatment | Northern America | | Qualitative | | Psychosocial |  |
| Elkington, Katherine S.; Hackler, Dusty; Walsh, Tracy A.; Latack, Jessica A.; Mckinnon, Karen; Borges, Cristiane; Wright, Eric R.; Wainberg, Milton L.; | 2013 | Perceived Mental Illness Stigma, Intimate Relationships, And Sexual Risk Behavior In Youth With Mental Illness | Northern America | | Qualitative | | Psychosocial |  |
| Elliott, L.; Bennett, A. S.; Szott, K.; Golub, A | 2018 | Competing Constructivisms: The Negotiation Of PTSD And Related Stigma Among Post-9/11 Veterans In New York City | Northern America | | Qualitative | | Service Use |  |
| Elliott, Marta; Doane, Michael J. | 2015 | Stigma Management Of Mental Illness: Effects Of Concealment, Discrimination, And Identification On Well-Being | Northern America | | Cross-Sectional | | Psychosocial |  |
| Ellouze, S, Jenhani, R, Bougacha, D, Turki, M, Aloulou, J, Ghachem, R, Au, C.H., Wong, C.M., Law, C.W., Et Al., Au, C.H., Wong, C.M., Law, C.W., Et Al., Aydemir, O., Ak... | 2022 | Self-Stigma And Functioning In Patients With Bipolar Disorder. | Northern Africa | | Cross-Sectional | | Psychosocial |  |
| Espinosa, R.; Valiente, C.; Rigabert, A.; Song, H. | 2016 | Recovery Style And Stigma In Psychosis: The Healing Power Of Integrating | Southern Europe | | Cross-Sectional | | Health |  |
| Evans-Lacko, S | 2011 | Association Between Public Views Of Mental Illness And Self-Stigma Among Individuals With Mental Illness In 14 European Countries | International | | Cross-Sectional | | Psychosocial |  |
| Evans-Lacko, S.; Clement, S.; Corker, E.; Brohan, E.; Dockery, L.; Farrelly, S.; Hamilton, S.; Pinfold, V.; Rose, D.; Henderson, C.; Thornicroft, G.; Mccrone, P.; | 2015 | How Much Does Mental Health Discrimination Cost: Valuing Experienced Discrimination In Relation To Healthcare Care Costs And Community Participation | Northern Europe | | Cross-Sectional | | Economical |  |
| Evans, E. J.; Hay, P. J.; Mond, J.; Paxton, S. J.; Quirk, F.; Rodgers, B.; Jhajj, A. K.; Sawoniewska, M. A. | 2011 | Barriers To Help-Seeking In Young Women With Eating Disorders: A Qualitative Exploration In A Longitudinal Community Survey | Australia And New Zealand | | Qualitative | | Service Use |  |
| Farrelly, S.; Clement, S.; Gabbidon, J.; Jeffery, D.; Dockery, L.; Lassman, F.; Brohan, E.; Henderson, R. C.; Williams, P.; Howard, L. M.; Thornicroft, G.; Grp, Miriad Study; | 2014 | Anticipated And Experienced Discrimination Amongst People With Schizophrenia, Bipolar Disorder And Major Depressive Disorder: A Cross Sectional Study | Northern Europe | | Cross-Sectional | | Psychosocial |  |
| Farrelly, S.; Jeffery, D.; Rusch, N.; Williams, P.; Thornicroft, G.; Clement, S.; | 2015 | The Link Between Mental Health-Related Discrimination And Suicidality: Service User Perspectives | Northern Europe | | Complex Cross-Sectional | | Health |  |
| Farrelly, S.; Jeffery, D.; Rusch, N.; Williams, P.; Thornicroft, G.; Clement, S.; | 2015 | The Link Between Mental Health-Related Discrimination And Suicidality: Service User Perspectives | Northern Europe | | Qualitative | | Economical |  |
| Feldhaus, T.; Falke, S.; Von Gruchalla, L.; Maisch, B.; Uhlmann, C.; Bock, E.; Lencer | 2018 | The Impact Of Self-Stigmatization On Medication Attitude In Schizophrenia Patients | Western Europe | | Cross-Sectional | | Psychosocial |  |
| Fennell, Dana; Liberato, Ana S.; | 2007 | Learning To Live With OCD: Labeling, The Self, The Stigma | Northern America | | Qualitative | | Psychosocial |  |
| Fernandez, Y. Garcia E.; Duberstein, P.; Paterniti, D. A.; Cipri, C. S.; Kravitz, R. L.; Epstein, R. M.; | 2012 | Feeling Labeled, Judged, Lectured, And Rejected By Family And Friends Over Depression: Cautionary Results For Primary Care Clinicians From A Multi-Centered, Qualitative Study | Northern America | | Qualitative | | Psychosocial |  |
| Fernando, S. M.; Deane, F. P.; Mcleod, H. J.; | 2017 | The Delaying Effect Of Stigma On Mental Health Help-Seeking In Sri Lanka | Southern Asia | | Cross-Sectional | | Service Use |  |
| Ferrari, M.; Flora, N.; Anderson, K. K.; Tuck, A.; Archie, S.; Kidd, S.; Mckenzie, K.; Team, A. C. E. Project; | 2015 | The African, Caribbean And European (ACE) Pathways To Care Study: A Qualitative Exploration Of Similarities And Differences Between African-Origin, Caribbean-Origin And European-Origin Groups In Pathways To Care For Psychosis | International | | Qualitative | | Service Use |  |
| Firmin, Ruth L, Zalzala, Aieyat B, Hamm, Jay A, Luther, Lauren, Lysaker, Paul H AI - Firmin, Ruth L. | 2020 | How Psychosis Interrupts The Lives Of Women And Men Differently: A Qualitative Comparison. | Northern America | | Qualitative | | Psychosocial |  |
| Forchuk, Cheryl; Nelson, Geoffrey; Hall, G. | 2006 | It's Important To Be Proud Of The Place You Live In: Housing Problems And Preferences Of Psychiatric Survivor | Northern America | | Qualitative | | Economical |  |
| Forthal, Sarah; Fekadu, Abebaw; Medhin, Girmay; Selamu, Medhin; Thornicroft, Graham; Hanlon, Charlotte; | 2019 | Rural Vs Urban Residence And Experience Of Discrimination Among People With Severe Mental Illnesses In Ethiopia | Eastern Africa | | Cross-Sectional | | Psychosocial |  |
| Fowler, Christopher A.; Rempfer, Melisa V.; Murphy, Meghan E.; Barnes, Amy L.; Hoover, Elise D.; | 2015 | Exploring The Paradoxical Effects Of Insight And Stigma In Psychological Recovery | Northern America | | Cross-Sectional | | Psychosocial |  |
| Frankhouser, T. L.; Defenbaugh, N. L. | 2017 | An Autoethnographic Examination Of Postpartum Depression | Northern America | | Qualitative | | Service Use |  |
| Franz, L.; Carter, T.; Leiner, A. S.; Bergner, E.; Thompson, N. J.; Compton, M | 2010 | Stigma And Treatment Delay In First-Episode Psychosis: A Grounded Theory Studyz | Northern America | | Qualitative | | Service Use |  |
| Frieh, Emma C AI - Frieh, Emma C | 2019 | Stigma, Trauma And Sexuality: The Experiences Of Women Hospitalised With Serious Mental Illness | Northern America | | Cross-Sectional | | Psychosocial |  |
| Fukuda, Claudia Cristina; Penso, Maria Aparecida; Do Amparo, Deise Matos; De Almeida, Bruno Coimbras; De Aquino Morais, Camila | 2016 | Mental Health Of Young Brazilians: Barriers To Professional Help-Seeking | South America | | Cross-Sectional | | Service Use |  |
| Fung, K. M.; Tsang, H. W.; Chan, F. | 2010 | Self-Stigma, Stages Of Change And Psychosocial Treatment Adherence Among Chinese People With Schizophrenia: A Path Analysis | Eastern Asia | | Complex Cross-Sectional | | Service Use |  |
| Fung, Kelvin M.; Tsang, Hector W.; Corrigan, Patrick W.; Lam, Chow S.; Cheng, Wai-Ming | 2007 | Measuring Self-Stigma Of Mental Illness In China And Its Implications For Recovery | Eastern Asia | | Cross-Sectional | | Service Use |  |
| Gabbidon, J.; Farrelly, S.; Hatch, S. L.; Henderson, C.; Williams, P.; Bhugra, D.; Dockery, L.; Lassman, F.; Thornicroft, G.; Clement, S. | 2014 | Discrimination Attributed To Mental Illness Or Race-Ethnicity By Users Of Community Psychiatric Services | Northern Europe | | Cross-Sectional | | Economical |  |
| Gaziel, M.; Hasson-Ohayon, I.; Morag-Yaffe, M.; Schapir, L.; Zalsman, G.; Shoval, G. | 2015 | Insight And Satisfaction With Life Among Adolescents With Mental Disorders: Assessing Associations With Self-Stigma And Parental Insight | Western Asia | | Cross-Sectional | | Psychosocial |  |
| Givens, J. L.; Tjia, J. | 2002 | Depressed Medical Students' Use Of Mental Health Services And Barriers To Use | Northern America | | Cross-Sectional | | Service Use |  |
| Gladman, B.; Waghorn, G.; | 2016 | Personal Experiences Of People With Serious Mental Illness When Seeking, Obtaining And Maintaining Competitive Employment In Queensland, Australia | Australia And New Zealand | | Qualitative | | Economical |  |
| Goetter, Elizabeth M.; Frumkin, Madelyn R.; Palitz, Sophie A.; Swee, Michaela B.; Baker, Amanda W.; Bui, Eric; Simon, Naomi M.; | 2018 | Barriers To Mental Health Treatment Among Individuals With Social Anxiety Disorder And Generalized Anxiety Disorder | Northern America | | Cross-Sectional | | Psychosocial |  |
| Gonzales, L.; Yanos, P. T.; Stefancic, A.; Alexander, M. J.; Harney-Delehanty, B.; | 2018 | The Role Of Neighborhood Factors And Community Stigma In Predicting Community Participation Among Persons With Psychiatric Disabilities | Northern America | | Cross-Sectional | | Psychosocial |  |
| Gonzalez-Torres, M. A.; Oraa, R.; Aristegui, M.; Fernandez-Rivas, A.; Guimon, J.; | 2007 | Stigma And Discrimination Towards People With Schizophrenia And Their Family Members. A Qualitative Study With Focus Groups | Southern Europe | | Qualitative | | Psychosocial |  |
| Gorman, L. A.; Blow, A. J.; Ames, B. D.; Reed, P. L. | 2011 | National Guard Families After Combat: Mental Health, Use Of Mental Health Services, And Perceived Treatment Barriers | Northern America | | Cross-Sectional | | Service Use |  |
| Graf, J.; Lauber, C.; Nordt, C.; Ruesch, P.; Meyer, P. C.; Rossler, W.; | 2004 | Perceived Stigmatization Of Mentally Ill People And Its Consequences For The Quality Of Life In A Swiss Population | Western Europe | | Cross-Sectional | | Psychosocial |  |
| Griffiths, S.; Mitchison, D.; Murray, S. B.; Mond, J. M.; Bastian, B. B. | 2018 | How Might Eating Disorders Stigmatization Worsen Eating Disorders Symptom Severity? Evaluation Of A Stigma Internalization Model | International | | Complex Cross-Sectional | | Health |  |
| Griffiths, S.; Mond, J. M.; Li, Z.; Gunatilake, S.; Murray, S. B.; Sheffield, J.; Touyz, S.; | 2015 | Self-Stigma Of Seeking Treatment And Being Male Predict An Increased Likelihood Of Having An Undiagnosed Eating Disorder | International | | Cross-Sectional | | Service Use |  |
| Griffiths, S.; Mond, J. M.; Murray, S. B.; Touyz, S.; | 2015 | The Prevalence And Adverse Associations Of Stigmatization In People With Eating Disorders | International | | Cross-Sectional | | Service Use |  |
| Grover, S.; Avasthi, A.; Singh, A.; Dan, A.; Neogi, R.; Kaur, D.; Lakdawala, B.; Rozatkar, A. R.; Nebhinani, N.; Patra, S.; Sivashankar, P.; Subramanyam, A. A.; Tripathi, A.; Gania, A.; Singh, G. P.; Behere, P.; | 2017 | Stigma Experienced By Patients With Severe Mental Disorders: A Nationwide Multicentric Study From India | Southern Asia | | Cross-Sectional | | Economical |  |
| Grover, S.; Hazari, N.; Aneja, J.; Chakrabarti, S.; Sharma, S.; Avasthi, A.; | 2016 | Recovery And Its Correlates Among Patients With Bipolar Disorder: A Study From A Tertiary Care Centre In North India | Southern Asia | | Cross-Sectional | | Psychosocial |  |
| Grover, Sandeep; Sahoo, Swapnajeet; Chakrabarti, Subho; Avasthi, Ajit; | 2018 | Association Of Internalized Stigma And Insight In Patients With Schizophrenia | Southern Asia | | Cross-Sectional | | Health |  |
| Guo, Y.; Qu, S. M.; Qin, H. Y.; | 2018 | Study Of The Relationship Between Self-Stigma And Subjective Quality Of Life For Individuals With Chronic Schizophrenia In The Community | Eastern Asia | | Cross-Sectional | | Psychosocial |  |
| Gupta, Nilu; Mohanty, Sandhyrani | 2016 | Stigma And Expressed Emotion In Spouses Of Schizophrenic Patients | Southern Asia | | Cross-Sectional | | Psychosocial |  |
| Gwaikolo, W. S.; Kohrt, B. A.; Cooper, J. L.; | 2017 | Health System Preparedness For Integration Of Mental Health Services In Rural Liberia | Western Africa | | Qualitative | | Structural |  |
| Habtamu, K.; Alem, A.; Medhin, G.; Fekadu, A.; Hanlon, C. | 2018 | Functional Impairment Among People With Severe And Enduring Mental Disorder In Rural Ethiopia: A Cross-Sectional Study | Eastern Africa | | Cross-Sectional | | Psychosocial |  |
| Hackler, Ashley H.; Vogel, David L.; Wade, Nathaniel G.; | 2010 | Attitudes Toward Seeking Professional Help For An Eating Disorder: The Role Of Stigma And Anticipated Outcomes | Northern America | | Cross-Sectional | | Service Use |  |
| Hailemariam, Maji; Ghebrehiwet, Senait; Baul, Tithi; Restivo, Juliana L.; Shibre, Teshome; Henderson, David C.; Girma, Eshetu; Fekadu, Abebaw; Teferra, Solomon; Hanlon, Charlotte; Johnson, Jennifer E.; Borba, Christina P.; | 2019 | He Can Send Her To Her Parents: The Interaction Between Marriageability, Gender And Serious Mental Illness In Rural Ethiopia | Eastern Africa | | Qualitative | | Psychosocial |  |
| Hall, T.; Kakuma, R.; Palmer, L.; Minas, H.; Martins, J.; Kermode, M | 2019 | Social Inclusion And Exclusion Of People With Mental Illness In Timor-Leste: A Qualitative Investigation With Multiple Stakeholders | South-Eastern Asia | | Qualitative | | Economical |  |
| Hamilton, S.; Pinfold, V.; Cotney, J.; Couperthwaite, L.; Matthews, J.; Barret, K.; Warren, S.; Corker, E.; Rose, D.; Thornicroft, G.; Henderson, C | 2016 | Qualitative Analysis Of Mental Health Service Users' Reported Experiences Of Discrimination | Northern Europe | | Qualitative | | Health |  |
| Hamilton, Sarah; Lewis-Holmes, Elanor; Pinfold, Vanessa | 2014 | Discrimination Against People With A Mental Health Diagnosis: Qualitative Analysis Of Reported Experiences | Northern Europe | | Qualitative | | Psychosocial |  |
| Hampson, M.; Hicks, R.; Watt, B.; | 2016 | Understanding The Employment Barriers And Support Needs Of People Living With Psychosis | Australia And New Zealand | | Qualitative | | Economical |  |
| Hanafiah, Ainul Nadhirah; Van Bortel, Tine; | 2015 | A Qualitative Exploration Of The Perspectives Of Mental Health Professionals On Stigma And Discrimination Of Mental Illness In Malaysia | South-Eastern Asia | | Qualitative | | Service Use |  |
| Handley, Tonelle E.; Kay-Lambkin, Frances J.; Inder, Kerry J.; Lewin, Terry J.; Attia, John R.; Fuller, Jeffrey; Perkins, David; Coleman, Clare; Weaver, Natasha; Kelly, Brian J. | 2014 | Self-Reported Contacts For Mental Health Problems By Rural Residents: Predicted Service Needs, Facilitators And Barriers | Australia And New Zealand | | Cross-Sectional | | Health |  |
| Hansen, M. C.; Cabassa, L. J.; | 2012 | Pathways To Depression Care: Help-Seeking Experiences Of Low-Income Latinos With Diabetes And Depression | Northern America | | Qualitative | | Service Use |  |
| Hansson, L.; Bjorkman, T.; | 2005 | Empowerment In People With A Mental Illness: Reliability And Validity Of The Swedish Version Of An Empowerment Scale | Northern Europe | | Cross-Sectional | | Psychosocial |  |
| Hansson, L.; Stjernsward, S.; Svensson, B.; | 2014 | Perceived And Anticipated Discrimination In People With Mental Illness--An Interview Study | Northern Europe | | Cross-Sectional | | Psychosocial |  |
| Harangozo, J.; Reneses, B.; Brohan, E.; Sebes, J.; Csukly, G.; Lopez-Ibor, J.; Sartorius, N.; Rose, D.; Thornicroft, G.; | 2014 | Stigma And Discrimination Against People With Schizophrenia Related To Medical Services | International | | Cross-Sectional | | Psychosocial |  |
| Hasan, A. A.; Musleh, M. | 2017 | Barriers To Seeking Early Psychiatric Treatment Amongst First-Episode Psychosis Patients: A Qualitative Study | Western Asia | | Qualitative | | Service Use |  |
| Haynes, Tiffany F.; Cheney, Ann M.; Sullivan, J.; Bryant, Keneshia; Curran, Geoffrey M.; Olson, Mary; Cottoms, Naomi; Reaves, Christina; | 2017 | Addressing Mental Health Needs: Perspectives Of African Americans Living In The Rural South | Northern America | | Qualitative | | Service Use |  |
| Hayward, P.; Wong, G.; Bright, J. A.; Lam, D. | 2002 | Stigma And Self-Esteem In Manic Depression: An Exploratory Study | Northern Europe | | Cross-Sectional | | Psychosocial |  |
| Heredia Montesinos, A.; Rapp, M. A.; Temur-Erman, S.; Heinz, A.; Hegerl, U.; Schouler-Ocak, M.; | 2015 | The Influence Of Stigma On Depression, Overall Psychological Distress, And Somatization Among Female Turkish Migrants | Western Europe | | Cross-Sectional | | Health |  |
| Heydari, A.; Saadatian, V.; Soodmand, P | 2017 | Black Shadow Of Stigma: Lived Experiences Of Patients With Psychiatric Disorders On The Consequences Of Stigma | Southern Asia | | Qualitative | | Psychosocial |  |
| Hill, S. K.; Cantrell, P.; Edwards, J.; Dalton, W.; | 2016 | Factors Influencing Mental Health Screening And Treatment Among Women In A Rural South Central Appalachian Primary Care Clinic Qualitative | Northern America | | Qualitative | | Service Use |  |
| Hipes, C.; Lucas, J.; Phelan, J. C.; White, R. C.; | 2016 | The Stigma Of Mental Illness In The Labor Market | Northern America | | Complex Cross-Sectional | | Economical |  |
| Ho, Henry C. Y, Chan, Chung Ho, Chan, Ying Chuen, Chan, Kevin Ka Shing AI | 2025 | Perceived Workplace Discrimination And Recovery Of People With Mental Illness: The Mediating Roles Of Self-Stigma, Organizational Justice, And Psychological Capital. | East Asia | | Complex Cross-Sectional | | Economical |  |
| Ho, W. W.; Chiu, M. Y.; Lo, W. T.; Yiu, M. G.; | 2010 | Recovery Components As Determinants Of The Health-Related Quality Of Life Among Patients With Schizophrenia: Structural Equation Modelling Analysis | Eastern Asia | | Cross-Sectional | | Psychosocial |  |
| Hofer, Alex; Post, Fabienne; Pardeller, Silvia; Frajo-Apor, Beatrice; Hoertnagl, Christine M.; Kemmler, Georg; Fleischhacker, W.; | 2019 | Self-Stigma Versus Stigma Resistance In Schizophrenia: Associations With Resilience, Premorbid Adjustment, And Clinical Symptoms | Western Europe | | Cross-Sectional | | Psychosocial |  |
| Hoge, C. W.; Castro, C. A.; Messer, S. C.; Mcgurk, D.; Cotting, D. I.; Koffman, R. L. | 2004 | Combat Duty In Iraq And Afghanistan, Mental Health Problems, And Barriers To Care | Northern America | | Cross-Sectional | | Service Use |  |
| Hoge, C. W.; Grossman, S. H.; Auchterlonie, J. L.; Riviere, L. A.; Milliken, C. S.; Wilk, J. E.; | 2014 | PTSD Treatment For Soldiers After Combat Deployment: Low Utilization Of Mental Health Care And Reasons For Dropout | Northern America | | Cross-Sectional | | Service Use |  |
| Holley, Lynn C.; Oh, Hyunsung; Thomas, De'Shay; | 2019 | Mental Illness Discrimination And Support Experienced By People Who Are Of Color And/Or LGB: Considering Intersecting Identities | Northern America | | Qualitative | | Psychosocial |  |
| Holubova, M.; Prasko, J.; Latalova, K.; Ociskova, M.; Grambal, A.; Kamaradova, D.; Vrbova, K.; Hruby, R.; | 2016 | Are Self-Stigma, Quality Of Life, And Clinical Data Interrelated In Schizophrenia Spectrum Patients? A Cross-Sectional Outpatient Study | Eastern Europe | | Cross-Sectional | | Psychosocial |  |
| Holubova, M.; Prasko, J.; Ociskova, M.; Kantor, K.; Vanek, J.; Slepecky, M.; Vrbova, K.; | 2019 | Quality Of Life, Self-Stigma, And Coping Strategies In Patients With Neurotic Spectrum Disorders: A Cross-Sectional Study | Eastern Europe | | Cross-Sectional | | Psychosocial |  |
| Holubova, Michaela; Prasko, Jan; Ociskova, Marie; Marackova, Marketa; Grambal, Ales; Slepecky, Milos; | 2016 | Self-Stigma And Quality Of Life In Patients With Depressive Disorder: A Cross-Sectional Study | Eastern Europe | | Cross-Sectional | | Psychosocial |  |
| Horsselenberg, E. M. A.; Van Busschbach, J. T.; Aleman, A.; Pijnenborg, G. H. M. | 2016 | Self-Stigma And Its Relationship With Victimization, Psychotic Symptoms And Self-Esteem Among People With Schizophrenia Spectrum Disorders | Western Europe | | Cross-Sectional | | Psychosocial |  |
| Howe, Lorna; Tickle, Anna; Brown, Ian; | 2018 | Schizophrenia Is A Dirty Word’ : Service Users’ Experiences Of Receiving A Diagnosis Of Schizophrenia | Western Europe | | Qualitative | | Psychosocial |  |
| Howland, M.; Levin, J.; Blixen, C.; Tatsuoka, C.; Sajatovic, M.; | 2016 | Mixed-Methods Analysis Of Internalized Stigma Correlates In Poorly Adherent Individuals With Bipolar Disorder | Northern America | | Qualitative | | Psychosocial |  |
| Hsiung, P. C.; Pan, A. W.; Liu, S. K.; Chen, S. C.; Peng, S. Y.; Chung, L.; | 2010 | Mastery And Stigma In Predicting The Subjective Quality Of Life Of Patients With Schizophrenia In Taiwan | Eastern Asia | | Cross-Sectional | | Psychosocial |  |
| Ilic, Marie; Reinecke, Jost; Bohner, Gerd; Rottgers, Hans-Onno; Beblo, Thomas; Driessen, Martin; Frommberger, Ulrich; Corrigan, Patrick William; | 2013 | Belittled, Avoided, Ignored, Denied: Assessing Forms And Consequences Of Stigma Experiences Of People With Mental Illness | Western Europe | | Longitudinal | | Health |  |
| Interian, A.; Martinez, I. E.; Guarnaccia, P. J.; Vega, W. A.; Escobar, J. I.; | 2007 | A Qualitative Analysis Of The Perception Of Stigma Among Latinos Receiving Antidepressants | Northern America | | Qualitative | | Health |  |
| Jack Tsai, Phd, Julia M. Whealin, Phd, And Robert H. Pietrzak, Phd, MPH | 2014 | Asian American And Pacific Islander Military Veterans In The United States: Health Service Use And Perceived Barriers To Mental Health Services | Northern America | | Cross-Sectional | | Service Use |  |
| Jack-Ide, I. O.; Uys, L.; | 2013 | Barriers To Mental Health Services Utilization In The Niger Delta Region Of Nigeria: Service Users' Perspectives | Western Africa | | Qualitative | | Service Use |  |
| Jahn, Danielle R.; Leith, Jaclyn; Muralidharan, Anjana; Brown, Clayton H.; Drapalski, Amy L.; Hack, Samantha; Lucksted, Alicia | 2019 | The Influence Of Experiences Of Stigma On Recovery: Mediating Roles Of Internalized Stigma, Self-Esteem, And Self-Efficacy | Northern America | | Complex Cross-Sectional | | Psychosocial |  |
| James, B. O.; Thomas, F. I.; Seb-Akahomen, O. J.; Igbinomwanhia, N. G.; Inogbo, C. F.; Thornicroft, G. | 2019 | Barriers To Care Among People With Schizophrenia Attending A Tertiary Psychiatric Hospital In Nigeria | Western Africa | | Cross-Sectional | | Service Use |  |
| James, T. T.; Kutty, V. R.; | 2015 | Assessment Of Internalized Stigma Among Patients With Mental Disorders In Thiruvananthapuram District, Kerala, India | Southern Asia | | Cross-Sectional | | Economical |  |
| Jansen, J. E.; Pedersen, M. B.; Hastrup, L. H.; Haahr, U. H.; Simonsen, E.; | 2018 | Important First Encounter: Service User Experience Of Pathways To Care And Early Detection In First-Episode Psychosis | Northern Europe | | Qualitative | | Service Use |  |
| Jansen, J. E.; Woldike, P. M.; Haahr, U. H.; Simonsen | 2015 | Service User Perspectives On The Experience Of Illness And Pathway To Care In First-Episode Psychosis: A Qualitative Study Within The TOP Project | Northern Europe | | Qualitative | | Service Use |  |
| Jazmín Mora-Ríos,1 Natalia Bautista1 | 2014 | Estigma Estructural, Género E Interseccionalidad. Implicaciones En La Atención A La Salud Mental | South America | | Qualitative | | Service Use |  |
| Jo Anne Sirey,., Martha L. Bruce, George S. Alexopoulos., Deborah A. Perlick, Ph.D., Patrick Raue, Ph.D., Steven J. Friedman, M.S., And Barnett S. Meyers, M.D. | 2001 | Perceived Stigma As A Predictor Of Treatment Discontinuation In Young And Older Outpatients With Depression | Northern America | | Cross-Sectional | | Health |  |
| Johnson, M.; Mills, T. L.; Deleon, J. M.; Hartzema, A. G.; Haddad, J.; | 2019 | Lives In Isolation: Stories And Struggles Of Low-Income African American Women With Panic Disorder | Northern America | | Qualitative | | Service Use |  |
| Jon-Ubabuco, N.; Champion, J. D.; | 2019 | Perceived Mental Healthcare Barriers And Health-Seeking Behavior Of African-American Caregivers Of Adolescents With Mental Health Disorders | Northern America | | Qualitative | | Service Use |  |
| Jormfeldt, H.; Arvidsson, B.; Svensson, B.; Hansson, L.; | 2008 | Construct Validity Of A Health Questionnaire Intended To Measure The Subjective Experience Of Health Among Patients In Mental Health Services | Northern Europe | | Cross-Sectional | | Health |  |
| Juurlink, T. T.; Vukadin, M.; Stringer, B.; Westerman, M. J.; Lamers, F.; Anema, J. R.; Bee | 2019 | Barriers And Facilitators To Employment In Borderline Personality Disorder: A Qualitative Study Among Patients, Mental Health Practitioners And Insurance Physicians | Western Europe | | Qualitative | | Economical |  |
| Kaewprom, C.; Curtis, J.; Deane, F. P. | 2011 | Factors Involved In Recovery From Schizophrenia: A Qualitative Study Of Thai Mental Health Nurses | South-Eastern Asia | | Qualitative | | Health |  |
| Kahng, S. K.; Mowbray, C. T.; | 2005 | Psychological Traits And Behavioral Coping Of Psychiatric Consumers: The Mediating Role Of Self-Esteem | Northern America | | Complex Cross-Sectional | | Psychosocial |  |
| Kahng, S. K.; Mowbray, C. T.; | 2005 | What Affects Self-Esteem Of Persons With Psychiatric Disabilities: The Role Of Causal Attributions Of Mental Illnesses | Northern America | | Cross-Sectional | | Psychosocial |  |
| Kalisova, L.; Michalec, J.; Hadjipapanicolaou, D.; Raboch, J.; | 2018 | Factors Influencing The Level Of Self-Stigmatisation In People With Mental Illness | Eastern Europe | | Cross-Sectional | | Health |  |
| Kamaradova, D.; Latalova, K.; Prasko, J.; Kubinek, R.; Vrbova, K.; Mainerova, B.; Cinculova, A.; Ociskova, M.; Holubova, M.; Smoldasova, J.; Tichackova, A. | 2016 | Connection Between Self-Stigma, Adherence To Treatment, And Discontinuation Of Medication | Eastern Europe | | Cross-Sectional | | Health |  |
| Kao, Yu-Chen; Lien, Yin-Ju; Chang, Hsin-An; Wang, Sheng-Chiang; Tzeng, Nian-Sheng; Loh, Ching-Hui | 2016 | Evidence For The Indirect Effects Of Perceived Public Stigma On Psychosocial Outcomes: The Mediating Role Of Self-Stigma | Eastern Asia | | Cross-Sectional | | Psychosocial |  |
| Kapungwe, A.; Cooper, S.; Mwanza, J.; Mwape, L.; Sikwese, A.; Kakuma, R.; Lund, C.; Flisher, A. J.; Consortium, M. Happ Research Programme; | 2010 | Mental Illness--Stigma And Discrimination In Zambia | Eastern Africa | | Qualitative | | Psychosocial |  |
| Karakas, S. A.; Okanli, A.; Yilmaz, E.; | 2016 | The Effect Of Internalized Stigma On The Self Esteem In Patients With Schizophrenia | Western Asia | | Cross-Sectional | | Psychosocial |  |
| Karanci, N. A.; Aras, A.; Kumpasoglu, G. B.; Can, D.; Cakir, E.; Karaaslan, C.; Semerci, M.; Tuzun, D | 2019 | Living With Schizophrenia: Perspectives Of Turkish People With Schizophrenia From Two Patient Associations On How The Illness Affects Their Lives | Western Asia | | Qualitative | | Psychosocial |  |
| Karidi, M. V.; Stefanis, C. N.; Theleritis, C.; Tzedaki, M.; Rabavilas, A. D.; Stefanis, N. C.; 2010 | 2010 | Perceived Social Stigma, Self-Concept, And Self-Stigmatization Of Patient With Schizophrenia | Southern Europe | | Cross-Sectional | | Health |  |
| Karidi, M.; Vassilopoulou, D.; Savvidou, E.; Vitoratou, S.; Maillis, A.; Rabavilas, A.; Stefanis, C. | 2015 | Bipolar Disorder And Self-Stigma: A Comparison With Schizophrenia | Southern Europe | | Cross-Sectional | | Health |  |
| Keyes, Carly; Nolte, Lizette; Williams, Timothy I.; | 2018 | The Battle Of Living With Obsessive Compulsive Disorder: A Qualitative Study Of Young People's Experiences | Northern Europe | | Qualitative | | Service Use |  |
| Kim, P. Y.; Toblin, R. L.; Riviere, L. A.; Kok, B. C.; Grossman, S. H.; Wilk, J. E | 2016 | Provider And Nonprovider Sources Of Mental Health Help In The Military And The Effects Of Stigma, Negative Attitudes, And Organizational Barriers To Care | Northern America | | Cross-Sectional | | Service Use |  |
| Kim, W. J.; Song, Y. J.; Ryu, H. S.; Ryu, V.; Kim, J. M.; Ha, R. Y.; Lee, S. J.; Namkoong, K.; Ha, K.; Cho, H. S. | 2015 | Internalized Stigma And Its Psychosocial Correlates In Korean Patients With Serious Mental Illness | Eastern Asia | | Cross-Sectional | | Psychosocial |  |
| King, M.; Dinos, S.; Shaw, J.; Watson, R.; Stevens, S.; Passetti, F.; Weich, S.; Serfaty, M.; | 2007 | The Stigma Scale: Development Of A Standardised Measure Of The Stigma Of Mental Illness. | Northern Europe | | Cross-Sectional | | Psychosocial |  |
| Kira, I. A.; Lewandowski, L.; Ashby, J. S.; Templin, T.; Ramaswamy, V.; Mohanesh, J.; | 2014 | The Traumatogenic Dynamics Of Internalized Stigma Of Mental Illness Among Arab American, Muslim, And Refugee Clients | Northern America | | Complex Cross-Sectional | | Health |  |
| Kleim, B.; Vauth, R.; Adam, G.; Stieglitz, R. D.; Hayward, P.; Corrigan, P.; | 2008 | Perceived Stigma Predicts Low Self-Efficacy And Poor Coping In Schizophrenia | Western Europe | | Cross-Sectional | | Psychosocial |  |
| Kleintjes, S.; Lund, C.; Swartz, L.; | 2013 | Barriers To The Participation Of People With Psychosocial Disability In Mental Health Policy Development In South Africa: A Qualitative Study Of Perspectives Of Policy Makers, Professionals, Religious Leaders And Academics | Southern Africa | | Qualitative | | Service Use |  |
| Komiti, Angela; | 2006 | The Influence Of Stigma And Attitudes On Seeking Help From A GP For Mental Health Problems | Northern America | | Cross-Sectional | | Service Use |  |
| Kondrat, D. C.; Early, T. J | 2011 | Battling In The Trenches: Case Managers' Ability To Combat The Effects Of Mental Illness Stigma On Consumers' Perceived Quality Of Life | Northern America | | Cross-Sectional | | Psychosocial |  |
| Kondratova, L.; Koenig, D.; Mlada, K.; Winkler, P.; | 2019 | Correlates Of Negative Attitudes Towards Medication In People With Schizophrenia | Eastern Europe | | Cross-Sectional | | Service Use |  |
| Koschorke, M.; Padmavati, R.; Kumar, S.; Cohen, A.; Weiss, H. A.; Chatterjee, S.; Pereira, J.; Naik, S.; John, S.; Dabholkar, H.; Balaji, M.; Chavan, A.; Varghese, M.; Thara, R.; Thornicroft, G.; Patel, V.; | 2014 | Experiences Of Stigma And Discrimination Of People With Schizophrenia In India | Southern Asia | | Qualitative | | Health |  |
| Koskela, S.; Pettitt, B.; Drennan, V.; | 2016 | The Experiences Of People With Mental Health Problems Who Are Victims Of Crime With The Police In England: A Qualitative Study | Northern Europe | | Qualitative | | Psychosocial |  |
| Kranke, D. A.; Floersch, J.; Kranke, B. O.; Munson, M. R.; | 2011 | A Qualitative Investigation Of Self-Stigma Among Adolescents Taking Psychiatric Medication | Northern America | | Qualitative | | Psychosocial |  |
| Kranke, Derrick; Floersch, Jerry; Townsend, Lisa; Munson, Michelle; | 2010 | Stigma Experience Among Adolescents Taking Psychiatric Medication | Northern America | | Qualitative | | Psychosocial |  |
| Kristine Kahr Nilsson A, Pirathiv Kugathasan B, Krista Nielsen Straarup C | 2016 | Characteristics, Correlates And Outcomes Of Perceived Stigmatization In Bipolar Disorder Patients | Northern Europe | | Longitudinal | | Psychosocial |  |
| Krupchanka, D.; Chrtkova, D.; Vitkova, M.; Munzel, D.; Ciharova, M.; Ruzickova, T.; Winkler, P.; Janouskova, M.; Albanese, E.; Sartorius, N. | 2018 | Experience Of Stigma And Discrimination In Families Of Persons With Schizophrenia In The Czech Republic | Eastern Europe | | Qualitative | | Psychosocial |  |
| Kular, Ariana; Perry, Benjamin I.; Brown, Luke; Gajwani, Ruchika; Jasini, Rubina; Islam, Zoebia; Birchwood, Max; Singh, Swaran P.; | 2018 | Stigma And Access To Care In First-Episode Psychosis | Northern Europe | | Cross-Sectional | | Service Use |  |
| Kvrgic, S.; Cavelti, M.; Beck, E. M.; Rusch, N.; Vauth, R.; | 2013 | Therapeutic Alliance In Schizophrenia: The Role Of Recovery Orientation, Self-Stigma, And Insight | Western Europe | | Cross-Sectional | | Service Use |  |
| Kwan, Patchareeya P.; Soniega-Sherwood, Jennie; Esmundo, Shenazar; Watts, Jonathan; Pike, James; Sabado-Liwag, Melanie; Palmer, Paula H.; | 2019 | Access And Utilization Of Mental Health Services Among Pacific Islanders | Northern America | | Qualitative | | Service Use |  |
| Lacey, M.; Paolini, S.; Hanlon, M. C.; Melville, J.; Galletly, C.; Campbell, L. E.; | 2015 | Parents With Serious Mental Illness: Differences In Internalised And Externalised Mental Illness Stigma And Gender Stigma Between Mothers And Fathers | Australia And New Zealand | | Cross-Sectional | | Psychosocial |  |
| Ladd, W.; | 2018 | Born Out Of Fear: A Grounded Theory Study Of The Stigma Of Bipolar Disorder For New Mothers | Northern America | | Qualitative | | Economical |  |
| Lai, Y. M.; Hong, C. P.; Chee, C. Y.; | 2001 | Stigma Of Mental Illness. Descriptive | South-Eastern Asia | | Cross-Sectional | | Psychosocial |  |
| Lakeman, R.; Mcgowan, P.; Macgabhann, L.; Parkinson, M.; Redmond, M.; Sibitz, I.; Stevenson, C.; Walsh, J.; | 2012 | A Qualitative Study Exploring Experiences Of Discrimination Associated With Mental-Health Problems In Ireland | Northern Europe | | Qualitative | | Psychosocial |  |
| Laquidara, Jill R, Furgason, Katelyn, Banks, Lindsay M, Saavedra, Sophia, Lincoln, Sarah Hope | 2025 | Perceived Impacts Of Internalized Stigma In Individuals With Schizophrenia And Schizoaffective Disorder. | Northern America | | Cross-Sectional | | Service Use |  |
| Lasalvia, A.; Zoppei, S.; Bonetto, C.; Tosato, S.; Zanatta, G.; Cristofalo, D.; De Santi, K.; Bertani, M.; Bissoli, S.; Lazzarotto, L.; Ceccato, E.; Riolo, R.; Marangon, V.; Cremonese, C.; Boggian, I.; Tansella, M.; Ruggeri, M.; | 2014 | The Role Of Experienced And Anticipated Discrimination In The Lives Of People With First-Episode Psychosis | Southern Europe | | Cross-Sectional | | Psychosocial |  |
| Lasalvia, Antonio; Zoppei, Silvia; Bortel, Tine Van; | 2013 | Global Pattern Of Experienced And Anticipated Discrimination Reported By People With Major Depressive Disorder : A Cross-Sectional Survey | International | | Cross-Sectional | | Health |  |
| Lawn, S.; Mcmahon, J.; | 2015 | Experiences Of Care By Australians With A Diagnosis Of Borderline Personality Disorder | Australia And New Zealand | | Cross-Sectional | | Service Use |  |
| Lazear, K. J.; Pires, S. A.; Isaacs, M. R.; Chaulk, P.; Huang, L.; | 2008 | Depression Among Low-Income Women Of Color: Qualitative Findings From Cross-Cultural Focus Groups | Northern America | | Qualitative | | Service Use |  |
| Lee, A. M.; Simeon, D.; Cohen, L. J.; Samuel, J.; Steele, A.; Galynker, II; | 2011 | Predictors Of Patient And Caregiver Distress In An Adult Sample With Bipolar Disorder Seeking Family Treatment | Northern America | | Cross-Sectional | | Health |  |
| Lempp, H.; Abayneh, S.; Gurung, D.; Kola, L.; Abdulmalik, J.; Evans-Lacko, S.; Semrau, M.; Alem, A.; Thornicroft, G.; Hanlon, C. | 2018 | Service User And Caregiver Involvement In Mental Health System Strengthening In Low- And Middle-Income Countries: A Cross-Country Qualitative Study | Eastern Africa, Western Africa, Southern Asia | | Qualitative | | Service Use |  |
| Li, J.; Guo, Y. B.; Huang, Y. G.; Liu, J. W.; Chen, W.; Zhang, X. Y.; Evans-Lacko, S.; Thornicroft, G.; | 2017 | Stigma And Discrimination Experienced By People With Schizophrenia Living In The Community In Guangzhou, China | Eastern Asia | | Cross-Sectional | | Psychosocial |  |
| Li, Xu-Hong, Zhang, Tian-Ming, Yau, Yuen Yum, Wang, Yi-Zhou, Wong, Yin-Ling Irene, Yang, Lawrence, Tian, Xiao-Li, Chan, Cecilia Lai-Wan, Ran, Mao-Sheng AI - Wang, Yi-Zhou | 2020 | Peer-To-Peer Contact, Social Support And Self-Stigma Among People With Severe Mental Illness In Hong Kong. | Eastern Asia | | Cross-Sectional | | Psychosocial |  |
| Liegghio, Maria; Jaswal, Prableen; | 2015 | Police Encounters In Child And Youth Mental Health: Could Stigma Informed Crisis Intervention Training (CIT) For Parents Help? | Northern America | | Qualitative | | Psychosocial |  |
| Lien, Y. J.; Chang, H. A.; Kao, Y. C.; Tzeng, N. S.; Lu, C. W.; Loh, C. H.; | 2018 | Insight, Self-Stigma And Psychosocial Outcomes In Schizophrenia: A Structural Equation Modelling Approach | Eastern Asia | | Cross-Sectional | | Health |  |
| Lien, Y. J.; Chang, H. A.; Kao, Y. C.; Tzeng, N. S.; Lu, C. W.; Loh, C. H.; | 2018 | The Impact Of Cognitive Insight, Self-Stigma, And Medication Compliance On The Quality Of Life In Patients With Schizophrenia | Eastern Asia | | Cross-Sectional | | Psychosocial |  |
| Lien, Y. J.; Chang, H. A.; Kao, Y. C.; Tzeng, N. S.; Yeh, C. B.; Loh, C. H.; | 2018 | Self-Stigma Mediates The Impact Of Insight On Current Suicide Ideation In Suicide Attempters With Schizophrenia: Results Of A Moderated Mediation Approach | Eastern Asia | | Complex Cross-Sectional | | Health |  |
| Lim, L.; Goh, J.; Chan, Y. H. | 2019 | Internalized Stigma, Disclosure And Self-Esteem Among Psychiatric Patients In A General Hospital Outpatient Clinic | South-Eastern Asia | | Cross-Sectional | | Psychosocial |  |
| Lin, C. L. E., Kopelowicz, A., Chan, C. H., & Hsiung, P. C | 2008 | A Qualitative Inquiry Into The Taiwanese Mentally Ill Persons' Difficulties Living In The Community | Eastern Asia | | Qualitative | | Psychosocial |  |
| Lin, C. Y., Chang, C. C., Wu, T. H., & Wang, J. D | 2016 | Dynamic Changes Of Self-Stigma, Quality Of Life, Somatic Complaints, And Depression Among People With Schizophrenia: A Pilot Study Applying Kernel Smoothers | Eastern Asia | | Cross-Sectional | | Health |  |
| Lin, S. Y. | 2013 | Beliefs About Causes, Symptoms, And Stigma Associated With Severe Mental Illness Among 'Highly Acculturated' Chinese-American Patients | Northern America | | Qualitative | | Psychosocial |  |
| Lindsey, M. A.; Korr, W. S.; Broitman, M.; Bone, L.; Green, A.; Leaf, P. J | 2006 | Help-Seeking Behaviors And Depression Among African American Adolescent Boys | Northern America | | Qualitative | | Service Use |  |
| Link, B. G., Struening, E. L., Neese-Todd, S., Asmussen, S., & Phelan, J. C | 2001 | Stigma As A Barrier To Recovery: The Consequences Of Stigma For The Self-Esteem Of People With Mental Illnesses | Northern America | | Longitudinal | | Psychosocial |  |
| Link, B. G., Wells, J., Phelan, J. C., & Yang, L. | 2015 | Understanding The Importance Of "Symbolic Interaction Stigma": How Expectations About The Reactions Of Others Adds To The Burden Of Mental Illness Stigma | Northern America | | Cross-Sectional | | Psychosocial |  |
| Liu, Ying, Li, Ying | 2024 | Community Participation And Subjective Perception Of Recovery And Quality Of Life Among People With Serious Mental Illnesses: The Mediating Role Of Self-Stigma. | East Asia | | Complex Cross-Sectional | | Economical |  |
| Livingston, J | 2012 | Self-Stigma And Quality Of Life Among People With Mental Illness Who Receive Compulsory Community Treatment Services | Northern America | | Longitudinal | | Psychosocial |  |
| Lloyd, C., Sullivan, D., & Williams, P. L. | 2005 | Perceptions Of Social Stigma And Its Effect On Interpersonal Relationships Of Young Males Who Experience A Psychotic Disorder | Australia And New Zealand | | Qualitative | | Psychosocial |  |
| Loch, A. A. | 2012 | Stigma And Higher Rates Of Psychiatric Re-Hospitalization: Sao Paulo Public Mental Health System | South America | | Longitudinal | | Health |  |
| Loganathan, S., & Murthy, R. S | 2011 | Living With Schizophrenia In India: Gender Perspectives | Southern Asia | | Qualitative | | Psychosocial |  |
| Loganathan, S., & Murthy, S. R | 2008 | Experiences Of Stigma And Discrimination Endured By People Suffering From Schizophrenia | Southern Asia | | Qualitative | | Psychosocial |  |
| Lorona, R. T., Fergus, T. A., Valentiner, D. P., Miller, L. M., & Mcgrath, P. B | 2018 | Self-Stigma And Etiological Attributions About Symptoms Among Individuals Diagnosed With An Anxiety Disorder: Relations With Symptom Severity And Symptom Improvement Following CBT | Northern America | | Cross-Sectional | | Health |  |
| Loughhead M, Guy S, Furber G & Segal L | 2018 | Consumer Views On Youth-Friendly Mental Health Services In South Australia | Australia And New Zealand | | Qualitative | | Service Use |  |
| Lueck, J. A | 2019 | What's The Risk In Seeking Help For Depression? Assessing The Nature And Pleasantness Of Outcome Perceptions Among Individuals With Depressive Symptomatology | Northern America | | Qualitative | | Service Use |  |
| Lund, Crick; Kleintjes, Sharon; Cooper, Sara; Petersen, Inge; Bhana, Arvin; Flisher, Alan J.; | 2011 | Challenges Facing South Africa's Mental Health Care System: Stakeholders' Perceptions Of Causes And Potential Solutions | | Southern Africa | | Qualitative | | Service Use |
| Lundberg, B., Hansson, L., Wentz, E., & Björkman, T | 2008 | Stigma, Discrimination, Empowerment And Social Networks: A Preliminary Investigation Of Their Influence On Subjective Quality Of Life In A Swedish Sample | Northern Europe | | Cross-Sectional | | Psychosocial |  |
| Lundberg, B., Hansson, L., Wentz, E., & Björkman, T. | 2009 | Are Stigma Experiences Among Persons With Mental Illness, Related To Perceptions Of Self-Esteem, Empowerment And Sense Of Coherence? | Northern Europe | | Cross-Sectional | | Psychosocial |  |
| Lv, Y., Wolf, A., & Wang, X. | 2013 | Experienced Stigma And Self-Stigma In Chinese Patients With Schizophrenia | Eastern Asia | | Cross-Sectional | | Psychosocial |  |
| Lyons, C., Hopley, P., & Horrocks, J. | 2009 | A Decade Of Stigma And Discrimination In Mental Health: Plus Ca Change, Plus C'est La Meme Chose (The More Things Change, The More They Stay The Same) | Northern Europe | | Qualitative | | Psychosocial |  |
| Lysaker, P. H., Davis, L. W., Warman, D. M., Strasburger, A., & Beattie, N | 2007 | Stigma, Social Function And Symptoms In Schizophrenia And Schizoaffective Disorder: Associations Across 6 Months | Northern America | | Longitudinal | | Psychosocial |  |
| Lysaker, P. H., Tsai, J., Yanos, P., & Roe, D | 2008 | Associations Of Multiple Domains Of Self-Esteem With Four Dimensions Of Stigma In Schizophrenia | Northern America | | Cross-Sectional | | Psychosocial |  |
| Lysaker, P., Yanos, P., Outcalt, J., & Roe, D | 2010 | Association Of Stigma, Self-Esteem, And Symptoms With Concurrent And Prospective Assessment Of Social Anxiety In Schizophrenia | Northern America | | Cross-Sectional | | Psychosocial |  |
| M. Cynthialogsdonwayneusuimelissapinto-Foltzvivianleffler Rakestraw | 2009 | Intention To Seek Depression Treatment In Adolescent Mothers And A Comparison Group Of Adolescent Girls | Northern America | | Cross-Sectional | | Service Use |  |
| Machin, A.; Hider, S.; Dale, N.; Chew-Graham, C.; | 2017 | Improving Recognition Of Anxiety And Depression In Rheumatoid Arthritis: A Qualitative Study In A Community Clinic | Western Europe | | Qualitative | | Service Use |  |
| Magallares, A., Perez-Garin, D., & Molero, F | 2016 | Social Stigma And Well-Being In A Sample Of Schizophrenia Patients | Southern Europe | | Cross-Sectional | | Psychosocial |  |
| Maggiolo, D., Buizza, C., Vittorielli, M., Lanfredi, M., Rossi, G., Ricci, A., ... & Lasalvia, A | 2010 | Patterns Of Experienced And Anticipated Discrimination In Patients With Schizophremia. Italian Results From The INDIGO International Multisite Project] | Southern Europe | | Cross-Sectional | | Psychosocial |  |
| Maharjan, S., & Panthee, B. | 2019 | Prevalence Of Self-Stigma And Its Association With Self-Esteem Among Psychiatric Patients In A Nepalese Teaching Hospital: A Cross-Sectional Study | Southern Asia | | Cross-Sectional | | Psychosocial |  |
| Mahomed, F.; Stein, M. A.; Chauhan, A.; Pathare, S.; | 2018 | They Love Me, But They Don't Understand Me': Family Support And Stigmatisation Of Mental Health Service Users In Gujarat, India | Northern America | | Qualitative | | Psychosocial |  |
| Maier, A., Ernst, J. P., Müller, S., Gross, D., Zepf, F. D., Herpertz-Dahlmann, B., & Hagenah, U. | 2014 | Self-Perceived Stigmatization In Female Patients With Anorexia Nervosa-Results From An Explorative Retrospective Pilot Study Of Adolescents | Western Europe | | Cross-Sectional | | Service Use |  |
| Manos, R. C., Rusch, L. C., Kanter, J. W., & Clifford, L. M | 2009 | Depression Self-Stigma As A Mediator Of The Relationship Between Depression Severity And Avoidance | Northern America | | Cross-Sectional | | Health |  |
| Mansouri, L., & Dowell, D. A. | 1989 | Perceptions Of Stigma Among The Long-Term Mentally Ill | Northern America | | Cross-Sectional | | Psychosocial |  |
| Marcussen, K., Gallagher, M., & Ritter, C | 2019 | Mental Illness As A Stigmatized Identity | Northern America | | Complex Cross-Sectional | | Health |  |
| Marcussen, K., Ritter, C., & Munetz, M. R | 2010 | The Effect Of Services And Stigma On Quality Of Life For Persons With Serious Mental Illnesses | Northern America | | Cross-Sectional | | Psychosocial |  |
| Markowitz, F. E | 1998 | The Effects Of Stigma On The Psychological Well-Being And Life Satisfaction Of Persons With Mental Illness | Northern America | | Longitudinal | | Psychosocial |  |
| Marques, L., Leblanc, N. J., Weingarden, H. M., Timpano, K. R., Jenike, M., & Wilhelm, S. | 2010 | Barriers To Treatment And Service Utilization In An Internet Sample Of Individuals With Obsessive-Compulsive Symptoms | Northern America | | Cross-Sectional | | Service Use |  |
| Marques, L., Weingarden, H. M., Leblanc, N. J., & Wilhelm, S | 2011 | Treatment Utilization And Barriers To Treatment Engagement Among People With Body Dysmorphic Symptoms | Northern America | | Cross-Sectional | | Service Use |  |
| Marquez, J. A.; Garcia, J. I. R.; | 2013 | Family Caregivers' Narratives Of Mental Health Treatment Usage Processes By Their Latino Adult Relatives With Serious And Persistent Mental Illness | Northern America | | Qualitative | | Service Use |  |
| Marthoenis, M.; Aichberger, M. C.; Schouler-Ocak, M.; | 2016 | Patterns And Determinants Of Treatment Seeking Among Previously Untreated Psychotic Patients In Aceh Province, Indonesia: A Qualitative Study | Eastern Asia | | Qualitative | | Service Use |  |
| Mashiach-Eizenberg, M., Hasson-Ohayon, I., Yanos, P. T., Lysaker, P. H., & Roe, D | 2013 | Internalized Stigma And Quality Of Life Among Persons With Severe Mental Illness: The Mediating Roles Of Self-Esteem And Hope | Western Asia | | Complex Cross-Sectional | | Psychosocial |  |
| Masuch, T. V., Bea, M., Alm, B., Deibler, P., & Sobanski, E | 2019 | Internalized Stigma, Anticipated Discrimination And Perceived Public Stigma In Adults With Adhd | Western Europe | | Cross-Sectional | | Psychosocial |  |
| Mathias, K.; Kermode, M.; San Sebastian, M.; Koschorke, M.; Goicolea, I.; | 2015 | Under The Banyan Tree - Exclusion And Inclusion Of People With Mental Disorders In Rural North India | Southern Asia | | Qualitative | | Psychosocial |  |
| Mccoll, L. | 2007 | They Just Don't Care': The Experiences Of Mental Health Consumers In A Queensland Bush Community | Australia And New Zealand | | Qualitative | | Service Use |  |
| Mckenzie, Sarah K, Oliffe, John L, Black, Alice, Collings, Sunny AI - Black, Alice | 2022 | Men's Experiences Of Mental Illness Stigma Across The Lifespan: A Scoping Review. | Northern America | | Qualitative | | Psychosocial |  |
| Mellotte, Harriet; Murphy, Dominic; Rafferty, Laura; Greenberg, Neil; | 2017 | Pathways Into Mental Health Care For UK Veterans: A Qualitative Study | Western Europe | | Qualitative | | Service Use |  |
| Michalak, E. E.; Yatham, L. N.; Maxwell, V.; Hale, S.; Lam, R. W.; | 2007 | The Impact Of Bipolar Disorder Upon Work Functioning: A Qualitative Analysis | Northern America | | Qualitative | | Economical |  |
| Mittal, D.; Drummond, K. L.; Blevins, D.; Curran, G.; Corrigan, P.; Sullivan, G.; | 2013 | Stigma Associated With PTSD: Perceptions Of Treatment Seeking Combat Veterans | Northern America | | Qualitative | | Service Use |  |
| Montesinos, A. H., Rapp, M. A., Temur-Erman, S., Heinz, A., Hegerl, U., & Schouler-Ocak, M | 2012 | The Influence Of Stigma On Depression, Overall Psychological Distress, And Somatization Among Female Turkish Migrants | Western Europe | | Cross-Sectional | | Psychosocial |  |
| Morgades-Bamba, C. I., Fuster-Ruizdeapodaca, M. J., & Molero, F | 2019 | The Impact Of Internalized Stigma On The Well-Being Of People With Schizophrenia | Southern Europe | | Complex Cross-Sectional | | Psychosocial |  |
| Morgades-Bamba, C. I., Fuster-Ruizdeapodaca, M. J., & Molero, F. | 2019 | Internalized Stigma And Its Impact On Schizophrenia Quality Of Life | Southern Europe | | Complex Cross-Sectional | | Psychosocial |  |
| Moriarty, A., Jolley, S., Callanan, M. M., & Garety, P | 2012 | Understanding Reduced Activity In Psychosis: The Roles Of Stigma And Illness Appraisals | Northern Europe | | Cross-Sectional | | Psychosocial |  |
| Mosanya, T. J., Adelufosi, A. O., Adebowale, O. T., Ogunwale, A., & Adebayo, O. K. | 2014 | Self-Stigma, Quality Of Life And Schizophrenia: An Outpatient Clinic Survey In Nigeria | Western Africa | | Cross-Sectional | | Health |  |
| Moses, T. | 2010 | Being Treated Differently: Stigma Experiences With Family, Peers, And School Staff Among Adolescents With Mental Health Disorders | Northern America | | Qualitative | | Psychosocial |  |
| Mulfinger, N.; Rusch, N.; Bayha, P.; Muller, S.; Boge, I.; Sakar, V.; Krumm, S.; | 2019 | Secrecy Versus Disclosure Of Mental Illness Among Adolescents: I. The Perspective Of Adolescents With Mental Illness | Western Europe | | Qualitative | | Psychosocial |  |
| Muñoz, M., Sanz, M., Pérez-Santos, E., & De Los Ángeles Quiroga, M | 2011 | Proposal Of A Socio-Cognitive-Behavioral Structural Equation Model Of Internalized Stigma In People With Severe And Persistent Mental Illness | Southern Europe | | Complex Cross-Sectional | | Psychosocial |  |
| Nadeem, E., Lange, J. M., Edge, D., Fongwa, M., Belin, T., & Miranda, J. | 2007 | Does Stigma Keep Poor Young Immigrant And U.S.-Born Black And Latina Women From Seeking Mental Health Care? | Northern America | | Cross-Sectional | | Service Use |  |
| Nathalie Oexle, Nicolas Rüsch, Sandra Viering, Christine Wyss, Erich Seifritz, Ziyan Xu & Wolfram Kawohl | 2017 | Self-Stigma And Suicidality: A Longitudinal Study | Western Europe | | Longitudinal | | Health |  |
| Nieminen, Irja; Ramon, Shulamit; Dawson, Ian; Flores, Paz; Leahy, Eithne; Pedersen, Maria Louise; Kaunonen, Marja; | 2014 | Experiences Of Social Inclusion And Employment Of Mental Health Service Users In A European Union Project | International | | Qualitative | | Economical |  |
| Nithsdale, V., Davies, J., & Croucher, P | 2008 | Psychosis And The Experience Of Employment | Northern Europe | | Qualitative | | Economical |  |
| Norman, R. M., Windell, D., Lynch, J., & Manchanda, R | 2011 | Parsing The Relationship Of Stigma And Insight To Psychological Well-Being In Psychotic Disorders | Northern America | | Cross-Sectional | | Psychosocial |  |
| Nxumalo Ngubane, Siphiwe; Mcandrew, Sue; Collier, Elizabeth; | 2019 | The Experiences And Meanings Of Recovery For Swazi Women Living With "Schizophrenia" | Southern Africa | | Qualitative | | Economical |  |
| O'Dare, Kellie, Atwell, Leah, King, Erin, Dillard, Dana, Herzog, Joseph, Rotunda, Robert, Bowers, C., Beidel, D., Marks, M., Bowers, Britt, T. W., Mcfadden, A. C., Britt, Casas, J. B | 2024 | Subjective Experiences, Perceptions, And Meanings Associated With Barriers To Mental Seeking Health Care Among First Responders. | Northern America | | Qualitative | | Service Use |  |
| Ociskova, M., Prasko, J., & Kamaradova, D | 2015 | Relationship Between Personality And Self-Stigma In Mixed Neurotic Spectrum And Depressive Disorders - Cross Sectional Study | Eastern Europe | | Cross-Sectional | | Psychosocial |  |
| Ociskova, M., Prasko, J., Kamaradova, D., Grambal, A., Latalova, K., & Sigmundova, Z. | 2014 | Relationship Between Internalized Stigma And Treatment Efficacy In Mixed Neurotic Spectrum And Depressive Disorders | Eastern Europe | | Cross-Sectional | | Service Use |  |
| Ociskova, M., Prasko, J., Vrbova, K., Kasalova, P., Holubova, M., Grambal, A., & Machu, K. | 2018 | Self-Stigma And Treatment Effectiveness In Patients With Anxiety Disorders-A Mediation Analysis | Eastern Europe | | Cross-Sectional | | Service Use |  |
| Oexle, N., Ajdacic-Gross, V., Kilian, R., Müller, M., Rodgers, S., Xu, Z., ... & Rüsch, N | 2017 | Mental Illness Stigma, Secrecy And Suicidal Ideation | Western Europe | | Cross-Sectional | | Health |  |
| Oexle, N., Müller, M., Kawohl, W., Xu, Z., Viering, S., Wyss, C., ... & Rüsch, N | 2018 | Self-Stigma As A Barrier To Recovery: A Longitudinal Study | Western Europe | | Longitudinal | | Psychosocial |  |
| Olasoji, M.; Maude, P.; Mccauley, K.; | 2016 | A Journey Of Discovery: Experiences Of Carers Of People With Mental Illness Seeking Diagnosis And Treatment For Their Relative | Australia And New Zealand | | Qualitative | | Service Use |  |
| Olaw; E, T. I.; Ajayi, M. P.; Amoo, E. O.; Iruonagbe, C. T.; | 2019 | Variations In Family Attitudes Towards Coping With People Living With Mental Illness | Western Africa | | Qualitative | | Service Use |  |
| Olçun, Z., & Altun, Ö. Ş. | 2017 | The Correlation Between Schizophrenic Patients' Level Of Internalized Stigma And Their Level Of Hope | Western Asia | | Cross-Sectional | | Psychosocial |  |
| Oliffe, J. L., Han, C. S., Ogrodniczuk, J. S., Phillips, J. C., & Roy, P. | 2011 | Suicide From The Perspectives Of Older Men Who Experience Depression: A Gender Analysis | Northern America | | Qualitative | | Psychosocial |  |
| Oliveira, S. E., Carvalho, H., & Esteves, F. | 2016 | Internalized Stigma And Quality Of Life Domains Among People With Mental Illness: The Mediating Role Of Self-Esteem | Southern Europe | | Complex Cross-Sectional | | Psychosocial |  |
| Oshodi, Y. O., Abdulmalik, J., Ola, B., James, B. O., Bonetto, C., Cristofalo, D., ... & Thornicroft, G | 2014 | Pattern Of Experienced And Anticipated Discrimination Among People With Depression In Nigeria: A Cross-Sectional Study | Western Africa | | Cross-Sectional | | Psychosocial |  |
| Ow, C. Y., & Lee, B. O | 2015 | Relationships Between Perceived Stigma, Coping Orientations, Self-Esteem, And Quality Of Life In Patients With Schizophrenia | Eastern Asia | | Cross-Sectional | | Psychosocial |  |
| Pal, A., Sharan, P., & Chadda, R. K | 2017 | Internalized Stigma And Its Impact In Indian Outpatients With Bipolar Disorder | Southern Asia | | Cross-Sectional | | Economical |  |
| Pardo Cely, E. E., Fierro, M., & Ibánez Pinilla, M. | 2011 | Prevalence And Associated Factors Of Non-Adherence To Treatment In Bipolar Disorder | South America | | Cross-Sectional | | Health |  |
| Park, S. G., Bennett, M. E., Couture, S. M., & Blanchard, J. J | 2013 | Internalized Stigma In Schizophrenia: Relations With Dysfunctional Attitudes, Symptoms, And Quality Of Life | Northern America | | Cross-Sectional | | Psychosocial |  |
| Patra, Bichitra Nanda, Patil, Vaibhav, Balhara, Yatan Pal Singh, Khandelwal, Sudhir K AI - Balhara, Yatan Pal Singh | 2020 | Self-Stigma In Patients With Major Depressive Disorder: An Exploratory Study From India. | Southern Asia | | Cross-Sectional | | Psychosocial |  |
| Paul, S. | 2016 | Responses To Stigma-Related Stressors: A Qualitative Inquiry Into The Lives Of People Living With Schizophrenia In India | Southern Asia | | Qualitative | | Economical |  |
| Paul, Sayani; Nadkarni, Vimla V.; | 2014 | A Qualitative Study On Family Acceptance, Stigma And Discrimination Of Persons With Schizophrenia In An Indian Metropolis | Southern Asia | | Qualitative | | Psychosocial |  |
| Pearl, R. L., Forgeard, M. J., Rifkin, L., Beard, C., & Björgvinsson, T | 2017 | Internalized Stigma Of Mental Illness: Changes And Associations With Treatment Outcomes | Northern America | | Longitudinal | | Health |  |
| Pellet, J., Golay, P., Nguyen, A., Suter, C., Ismailaj, A., Bonsack, C., & Favrod, J | 2019 | The Relationship Between Self-Stigma And Depression Among People With Schizophrenia-Spectrum Disorders: A Longitudinal Study | Western Europe | | Longitudinal | | Health |  |
| Penas, Patricia, Uriarte, Jose Juan, Alvarez-Gonzalez, Alexander, Moreno-Calvete, Maria-Concepcion, Garay, Maria Asuncion, Iraurgi, Ioseba | 2024 | The Role Of Personal Recovery And Internalised Stigma On The Expression Of Symptomatology In Severe Mental Disorders: Mediating And Moderating Effects. | Southern Europe | | Cross-Sectional | | Health |  |
| Peng, Yanan, Xu, Ronghua, Li, Yan, Li, Ling, Song, Lanjun, Xi, Juzhe AI - Xi, Juzhe | 2024 | Dyadic Effects Of Stigma On Quality Of Life In People With Schizophrenia And Their Family Caregivers: Mediating Role Of Patients' Perception Of Caregivers' Expressed Emotion. | Eastern Asia | | Longitudinal | | Psychosocial |  |
| Pérez-Garín, D., Molero, F., & Bos, A. E | 2015 | Internalized Mental Illness Stigma And Subjective Well-Being: The Mediating Role Of Psychological Well-Being | Southern Europe | | Cross-Sectional | | Psychosocial |  |
| Pérez-Garín, D., Molero, F., & Bos, A. E. | 2017 | The Effect Of Personal And Group Discrimination On The Subjective Well-Being Of People With Mental Illness: The Role Of Internalized Stigma And Collective Action Intention | Southern Europe | | Complex Cross-Sectional | | Psychosocial |  |
| Perlick, D. A., Rosenheck, R. A., Clarkin, J. F., Sirey, J. A., Salahi, J., Struening, E. L., & Link, B. G | 2001 | Stigma As A Barrier To Recovery: Adverse Effects Of Perceived Stigma On Social Adaptation Of Persons Diagnosed With Bipolar Affective Disorder | Northern America | | Cross-Sectional | | Psychosocial |  |
| Pettersen, G., Rosenvinge, J. H., & Ytterhus, B | 2008 | The “Double Life” Of Bulimia: Patients’ Experiences In Daily Life Interactions | Northern Europe | | Qualitative | | Psychosocial |  |
| Phillips, M. R., Pearson, V., Li, F., Xu, M., & Yang, L. | 2002 | Stigma And Expressed Emotion: A Study Of People With Schizophrenia And Their Family Members In China | Eastern Asia | | Cross-Sectional | | Economical |  |
| Picco, L., Lau, Y. W., Pang, S., Abdin, E., Vaingankar, J. A., Chong, S. A., & Subramaniam, M. | 2017 | Mediating Effects Of Self-Stigma On The Relationship Between Perceived Stigma And Psychosocial Outcomes Among Psychiatric Outpatients: Findings From A Cross-Sectional Survey In Singapore | Eastern Asia | | Cross-Sectional | | Psychosocial |  |
| Picco, L., Pang, S., Lau, Y. W., Jeyagurunathan, A., Satghare, P., Abdin, E., ... & Subramaniam, M. | 2016 | Internalized Stigma Among Psychiatric Outpatients: Associations With Quality Of Life, Functioning, Hope And Self-Esteem | Eastern Asia | | Cross-Sectional | | Psychosocial |  |
| Pinfold, V.; Byrne, P.; Toulmin, H.; | 2005 | Challenging Stigma And Discrimination In Communities: A Focus Group Study Identifying UK Mental Health Service Users' Main Campaign Priorities | Western Europe | | Qualitative | | Psychosocial |  |
| Pitanupong, Jarurin, Aunjitsakul, Warut AI - Aunjitsakul, Warut | 2023 | Personal And Perceived Stigma In Relation To Diverse Domains Of Quality Of Life Among Patients With Major Depressive Disorder Having Residual Symptoms: A Hospital-Based Cross-Sectionalstudy In Thailand. | South-East Asia | | Cross-Sectional | | Health |  |
| Polacsek, M., Boardman, G. H., & Mccann, T. V | 2019 | Help-Seeking Experiences Of Older Adults With A Diagnosis Of Moderate Depression | Australia And New Zealand | | Qualitative | | Service Use |  |
| Prince, J. D., Oyo, A., Mora, O., Wyka, K., & Schonebaum, A. D. | 2018 | Loneliness Among Persons With Severe Mental Illness | Northern America | | Cross-Sectional | | Psychosocial |  |
| Prizeman, Katie, Mccabe, Ciara, Weinstein, Netta | 2025 | Internalized Stigma Is A Predictor Of Mental Health Secrecy And Loneliness In Young People With Clinical Depression Symptoms: A Longitudinal Study. | Northern America | | Longitudinal | | Psychosocial |  |
| Pyle, M., & Morrison, A. P. | 2017 | Internalised Stereotypes Across Ultra-High Risk Of Psychosis And Psychosis Populations | Northern Europe | | Cross-Sectional | | Psychosocial |  |
| Pyle, M., & Morrison, A. P. | 2014 | It's Just A Very Taboo And Secretive Kind Of Thing: Making Sense Of Living With Stigma And Discrimination From Accounts Of People With Psychosis | Northern Europe | | Qualitative | | Psychosocial |  |
| Pyle, M., Stewart, S. L., French, P., Byrne, R., Patterson, P., Gumley, A., ... & Morrison, A. P. | 2015 | Internalized Stigma, Emotional Dysfunction And Unusual Experiences In Young People At Risk Of Psychosis | Northern Europe | | Cross-Sectional | | Health |  |
| Pyne, J. M., Kuc, E. J., Schroeder, P. J., Fortney, J. C., Edlund, M., & Sullivan, G | 2004 | Relationship Between Perceived Stigma And Depression Severity | Northern America | | Cross-Sectional | | Health |  |
| Qin, Sang, Corrigan, Patrick, Margaglione, Maria, Smith, Andrew, Auch, Wongcsm, Lawcw, Wongmc, Chungkf, Bennettda, Bentlerpm, Bonettdg, Canadyva, Chiangyc, Liouth... | 2023 | Self-Stigma's Effect On Psychosocial Functioning Among People With Mental Illness. | Eastern Asia | | Cross-Sectional | | Psychosocial |  |
| Quinn, Neil; Wilson, Alistair; Macintyre, Gillian; Tinklin, Teresa; | 2009 | People Look At You Differently': Students' Experience Of Mental Health Support Within Higher Education | Western Europe | | Qualitative | | Psychosocial |  |
| Raguram, R., Raghu, T. M., Vounatsou, P., & Weiss, M. G | 2004 | Schizophrenia And The Cultural Epidemiology Of Stigma In Bangalore, India | Southern Asia | | Complex Cross-Sectional | | Psychosocial |  |
| Rayan, A., & Aldaieflih, M. T | 2019 | Public Stigma Toward Mental Illness And Its Correlates Among Patients Diagnosed With Schizophrenia | Western Asia | | Cross-Sectional | | Health |  |
| Razali, S. M., Hussein, S., & Ismail, T. A. T | 2010 | Perceived Stigma And Self-Esteem Among Patients With Schizophrenia | Southern Asia | | Cross-Sectional | | Psychosocial |  |
| Reavley, N. J., & Jorm, A. F. | 2015 | Experiences Of Discrimination And Positive Treatment In People With Mental Health Problems: Findings From An Australian National Survey | Australia And New Zealand | | Cross-Sectional | | Economical |  |
| Redmond, C., Larkin, M., & Harrop, C | 2010 | The Personal Meaning Of Romantic Relationships For Young People With Psychosis | Northern Europe | | Qualitative | | Psychosocial |  |
| Reupert, A.; Maybery, D.; | 2009 | Fathers' Experience Of Parenting With A Mental Illness | Australia And New Zealand | | Qualitative | | Structural |  |
| Rezaie, Leeba; Shafaroodi, Narges; Philips, David; | 2017 | The Barriers To Participation In Leisure Time Physical Activities Among Iranian Women With Severe Mental Illness: A Qualitative Study | Western Asia | | Qualitative | | Health |  |
| Rezayat, F., Mohammadi, E., Fallahi‐Khoshknab, M., & Sharifi, V | 2019 | Experience And The Meaning Of Stigma In Patients With Schizophrenia Spectrum Disorders And Their Families: A Qualitative Study | Western Asia | | Qualitative | | Psychosocial |  |
| Rezayat, Fatemeh; Mohammadi, Eesa; Fallahi-Khoshknab, Masoud; | 2019 | The Process Of Responding To Stigma In People With Schizophrenia Spectrum Disorders And Families: A Grounded Theory Study | Western Asia | | Qualitative | | Psychosocial |  |
| Ritsher, J. B., & Phelan, J. C | 2004 | Internalized Stigma Predicts Erosion Of Morale Among Psychiatric Outpatients | Northern America | | Longitudinal | | Health |  |
| Robinson, W. D.; Springer, P. R.; Bischoff, R.; Geske, J.; Backer, E.; Olson, M.; Jarzynka, K.; Swinton, J.; | 2012 | Rural Experiences With Mental Illness: Through The Eyes Of Patients And Their Families | Northern America | | Qualitative | | Service Use |  |
| Romero-Castillejo, Ana, Lopez-Carrilero, Raquel, Vila-Badia, Regina, Lamarca, Maria, Pousa, Esther, Grasa, Eva, Barajas, Ana, Barrigon, Maria Luisa, Birules, Irene, Pelaez, Trinidad, Gutierrez-Zotes, Alfonso, Lorente-Rovira, Ester, Cid, Jordi, Ruiz-Delgado, Isabel, Gonzalez-Higueras, Fermin, Legido, Teresa, Moreno-Kustner, Berta, Moritz, Steffen, Spanish Metacognition Group, Ochoa, Susana | 2024 | The Influence Of Metacognition, Social Cognition, Self-Esteem, And Quality Of Life On Personal Stigma In First-Episode Psychosis. | Southern Europe | | Cross-Sectional | | Psychosocial |  |
| Rose, D., Willis, R., Brohan, E., Sartorius, N., Villares, C., Wahlbeck, K., & Thornicroft, G., INDIGO | 2011 | Reported Stigma And Discrimination By People With A Diagnosis Of Schizophrenia | International | | Qualitative | | Psychosocial |  |
| Rosenfield, S. | 1997 | Labeling Mental Illness: The Effects Of Received Services And Perceived Stigma On Life Satisfaction | Northern America | | Cross-Sectional | | Psychosocial |  |
| Rüsch N, Corrigan PW, Todd AR, Bodenhausen GV | 2010 | Implicit Self-Stigma In People With Mental Illness | Northern America | | Cross-Sectional | | Psychosocial |  |
| Rusch, L. C., Kanter, J. W., Manos, R. C., & Weeks, C. E | 2008 | Depression Stigma In A Predominantly Low Income African American Sample With Elevated Depressive Symptoms | Northern America | | Cross-Sectional | | Health |  |
| Rüsch, N., Brohan, E., Gabbidon, J., Thornicroft, G., & Clement, S. | 2014 | Stigma And Disclosing One's Mental Illness To Family And Friends | Northern Europe | | Cross-Sectional | | Psychosocial |  |
| Rüsch, N., Corrigan, P. W., Wassel, A., Michaels, P., Larson, J. E., Olschewski, M., ... & Batia, K | 2009 | Self-Stigma, Group Identification, Perceived Legitimacy Of Discrimination And Mental Health Service Use | Northern America | | Longitudinal | | Health |  |
| Rüsch, N., Heekeren, K., Theodoridou, A., Müller, M., Corrigan, P. W., Mayer, B., ... & Rössler, W | 2015 | Stigma As A Stressor And Transition To Schizophrenia After One Year Among Young People At Risk Of Psychosis | Western Europe | | Longitudinal | | Health |  |
| Rüsch, N., Müller, M., Heekeren, K., Theodoridou, A., Metzler, S., Dvorsky, D., ... & Rössler, W | 2014 | Longitudinal Course Of Self-Labeling, Stigma Stress And Well-Being Among Young People At Risk Of Psychosis | Western Europe | | Longitudinal | | Psychosocial |  |
| Russinova, Zlatka; Griffin, Shanta; Bloch, Philippe; Wewiorski, Nancy J.; Rosoklija, Ilina; | 2011 | Workplace Prejudice And Discrimination Toward Individuals With Mental Illnesses | Northern America | | Qualitative | | Economical |  |
| Samuel, Ignatius A. | 2014 | Utilization Of Mental Health Services Among African-American Male Adolescents Released From Juvenile Detention: Examining Reasons For Within-Group Disparities In Help-Seeking Behaviors | Northern America | | Qualitative | | Service Use |  |
| Sandra E. H. Oliveira, Helena Carvalho & Francisco Esteves | 2016 | Internalized Stigma And Quality Of Life Domains Among People With Mental Illness: The Mediating Role Of Self-Esteem | Southern Europe | | Cross-Sectional | | Psychosocial |  |
| Sarısoy, G., Kaçar, Ö. F., Pazvantoğlu, O., Korkmaz, I. Z., Öztürk, A., Akkaya, D., ... & Sahin, A. R. | 2013 | Internalized Stigma And Intimate Relations In Bipolar And Schizophrenic Patients: A Comparative Study | Western Asia | | Cross-Sectional | | Psychosocial |  |
| Sathyanath M., Shashwath, Shanmukhappa, Sachin Beesanahalli, Kakunje, Anil, Nath, Santanu, Veetil, Mohanchandran Varikara, Alonso, J., Buron, A., Bruffaerts, R., He, Y., Posada-Villa, J., Lepine, J. P., Et Al., Bipeta, R... | 2023 | Stigma And Discrimination Among Persons With Mental Illness In A Tertiary Care Medical Institution In Southern India. | Southern Asia | | Cross-Sectional | | Psychosocial |  |
| Schomerus, G., Stolzenburg, S., Freitag, S., Speerforck, S., Janowitz, D., Evans-Lacko, S., ... & Schmidt, S. | 2019 | Stigma As A Barrier To Recognizing Personal Mental Illness And Seeking Help: A Prospective Study Among Untreated Persons With Mental Illness | Western Europe | | Longitudinal | | Service Use |  |
| Schulze, B., & Angermeyer, M. C. | 2003 | Subjective Experiences Of Stigma. A Focus Group Study Of Schizophrenic Patients, Their Relatives And Mental Health Professionals | Western Europe | | Qualitative | | Structural |  |
| Schuy, Katrin; Brants, Loni; Dors, Simone; Horzetzky, Marie; Willmund, Gerd; Zimmermann, Peter; Strohle, Andreas; Rau, Heinrich; Siegel, Stefan; | 2019 | Psychological Stigma Costs As Barriers To Healthcare Use In Former Soldiers Of The German Armed Forces: A Qualitative Analysis | Western Europe | | Qualitative | | Service Use |  |
| Sedlackova, Z., Kamarádová, D., Prasko, J., Látalová, K., Ocisková, M., Ocisková, M., & Vrbová, K. | 2015 | Treatment Adherence And Self-Stigma In Patients With Depressive Disorder In Remission - A Cross-Sectional Study | Eastern Europe | | Cross-Sectional | | Health |  |
| Segalovich, J., Doron, A., Behrbalk, P., Kurs, R., & Romem, P | 2013 | Internalization Of Stigma And Self-Esteem As It Affects The Capacity For Intimacy Among Patients With Schizophrenia | Western Asia | | Cross-Sectional | | Psychosocial |  |
| Shimotsu, S., & Horikawa, N. | 2016 | Self-Stigma In Depressive Patients: Association Of Cognitive Schemata, Depression, And Self-Esteem | Eastern Asia | | Complex Cross-Sectional | | Psychosocial |  |
| Shin, Y. J., Joo, Y. H., & Kim, J. H. | 2016 | Self-Perceived Cognitive Deficits And Their Relationship With Internalized Stigma And Quality Of Life In Patients With Schizophrenia | Eastern Asia | | Cross-Sectional | | Psychosocial |  |
| Sibitz, M. Amering. Unger, M.E. Seyringer, A. Bachmann B. Schrank, T. Benesch, B. Schulze, A. Woppmann | 2010 | The Impact Of The Social Network, Stigma And Empowerment On The Quality Of Life In Patients With Schizophrenia | Western Europe | | Cross-Sectional | | Psychosocial |  |
| Sickel, A. E., Seacat, J. D., & Nabors, N. A | 2019 | Mental Health Stigma: Impact On Mental Health Treatment Attitudes And Physical Health | Northern America | | Complex Cross-Sectional | | Service Use |  |
| Sirey, J. A., Bruce, M. L., Alexopoulos, G. S., Perlick, D. A., Friedman, S. J., & Meyers, B. S | 2001 | Stigma As A Barrier To Recovery: Perceived Stigma And Patient-Rated Severity Of Illness As Predictors Of Antidepressant Drug Adherence | Northern America | | Cross-Sectional | | Service Use |  |
| Srimongkon, P., Aslani, P., & Chen, T. F | 2018 | Consumer-Related Factors Influencing Antidepressant Adherence In Unipolar Depression: A Qualitative Study | Australia And New Zealand | | Qualitative | | Health |  |
| Subu, Muhammad Arsyad, Wati, Del Fatma, Netrida, Netrida, Priscilla, Vetty, Dias, Jacqueline Maria, Abraham, Mini Sarah, Slewa-Younan, Shameran, Al-Yateem, Nabeel AI - Subu, Muhammad Arsyad | 2021 | Types Of Stigma Experienced By Patients With Mental Illness And Mental Health Nurses In Indonesia: A Qualitative Content Analysis. | South-East Asia | | Qualitative | | Psychosocial |  |
| Szcześniak, D., Kobyłko, A., Wojciechowska, I., Kłapciński, M., & Rymaszewska, J | 2018 | Internalized Stigma And Its Correlates Among Patients With Severe Mental Illness | Eastern Europe | | Cross-Sectional | | Psychosocial |  |
| Tanaka, C., Tuliao, M. T. R., Tanaka, E., Yamashita, T., & Matsuo, H. | 2018 | A Qualitative Study On The Stigma Experienced By People With Mental Health Problems And Epilepsy In The Philippines | South-Eastern Asia | | Qualitative | | Health |  |
| Tang, I. C., & Wu, H. C | 2012 | Quality Of Life And Self-Stigma In Individuals With Schizophrenia | Eastern Asia | | Cross-Sectional | | Psychosocial |  |
| Teferra, S.; Hanlon, C.; Beyero, T.; Jacobsson, L.; Shibre, T.; | 2013 | Perspectives On Reasons For Non-Adherence To Medication In Persons With Schizophrenia In Ethiopia: A Qualitative Study Of Patients, Caregivers And Health Workers | Eastern Africa | | Qualitative | | Health |  |
| Thomé, E. S., Dargél, A. A., Migliavacca, F. M., Potter, W. A., Jappur, D. M. C., Kapczinski, F., & Ceresér, K. M | 2012 | Stigma Experiences In Bipolar Patients: The Impact Upon Functioning | South America | | Cross-Sectional | | Economical |  |
| Thompson, V. L. S., Noel, J. G., & Campbell, J. | 2004 | Stigmatization, Discrimination, And Mental Health: The Impact Of Multiple Identity Status | Northern America | | Cross-Sectional | | Health |  |
| Thornicroft, G., Brohan, E., Rose, D., Sartorius, N., Leese, M., & INDIGO Study Group | 2009 | Global Pattern Of Experienced And Anticipated Discrimination Against People With Schizophrenia: A Cross-Sectional Survey | International | | Cross-Sectional | | Economical |  |
| Touriño R, Acosta FJ, Giráldez A, Álvarez J, González JM, Abelleira C, Benítez N, Baena E, Fernández JA, Rodriguez CJ | 2018 | Suicidal Risk, Hopelessness And Depression In Patients With Schizophrenia And Internalized Stigma | Southern Europe | | Cross-Sectional | | Health |  |
| Trani, J. F., Bakhshi, P., Kuhlberg, J., Narayanan, S. S., Venkataraman, H., Mishra, N. N., ... & Deshpande, S. | 2015 | Mental Illness, Poverty And Stigma In India: A Case-Control Study | Southern Asia | | Cross-Sectional | | Economical |  |
| Tsang, H. W. H., Fung, K. M. T., & Chung, R. C. K. | 2010 | Self-Stigma And Stages Of Change As Predictors Of Treatment Adherence Of Individuals With Schizophrenia | Eastern Asia | | Cross-Sectional | | Service Use |  |
| Üçok, A., Brohan, E., Rose, D., Sartorius, N., Leese, M., Yoon, C. K., ... & INDIGO Study Group | 2012 | Anticipated Discrimination Among People With Schizophrenia | International | | Cross-Sectional | | Psychosocial |  |
| Uhlmann, C., Kaehler, J., Harris, M. S., Unser, J., Arolt, V., & Lencer, R. | 2014 | Negative Impact Of Self-Stigmatization On Attitude Toward Medication Adherence In Patients With Psychosis | Northern America | | Cross-Sectional | | Health |  |
| Van Schie, Charlotte C, Lewis, Kate, Barr, Karlen R, Jewell, Mahlie, Malcolmson, Natalie, Townsend, Michelle L, Grenyer, Brin F. S | 2024 | Borderline Personality Disorder And Stigma: Lived Experience Perspectives On Helpful And Hurtful Language. | Australia And New Zealand | | Qualitative | | Psychosocial |  |
| Van Zelst, C., Van Nierop, M., Oorschot, M., Myin-Germeys, I., Van Os, J., & Delespaul, P | 2014 | Stereotype Awareness, Self-Esteem And Psychopathology In People With Psychosis | Western Europe | | Cross-Sectional | | Psychosocial |  |
| Vass, V., Morrison, A. P., Law, H., Dudley, J., Taylor, P., Bennett, K. M., & Bentall, R. P | 2015 | How Stigma Impacts On People With Psychosis: The Mediating Effect Of Self-Esteem And Hopelessness On Subjective Recovery And Psychotic Experiences | Northern Europe | | Longitudinal | | Psychosocial |  |
| Vass, V., Sitko, K., West, S., & Bentall, R. P. | 2017 | How Stigma Gets Under The Skin: The Role Of Stigma, Self-Stigma And Self-Esteem In Subjective Recovery From Psychosis | Northern Europe | | Complex Cross-Sectional | | Psychosocial |  |
| Vauth, R., Kleim, B., Wirtz, M., & Corrigan, P. W | 2007 | Self-Efficacy And Empowerment As Outcomes Of Self-Stigmatizing And Coping In Schizophrenia | Western Europe | | Complex Cross-Sectional | | Psychosocial |  |
| Vázquez, G. H., Kapczinski, F., Magalhaes, P. V., Córdoba, R., Jaramillo, C. L., Rosa, A. R., ... & On Bipolar, T. I. A. N. | 2011 | Stigma And Functioning In Patients With Bipolar Disorder | South America | | Cross-Sectional | | Psychosocial |  |
| Verhaeghe, M., Bracke, P., & Christiaens, W. | 2010 | Stigma And Client Satisfaction In Mental Health Services | Western Europe | | Cross-Sectional | | Psychosocial |  |
| Vidojević, I. M., Jočić, D. Ð., & Tošković, O. | 2012 | Comparative Study Of Experienced And Anticipated Stigma In Serbia And The World | Eastern Europe | | Cross-Sectional | | Psychosocial |  |
| Violeau, Louis, Dudilot, Anthony, Roux, Solenne, Prouteau, Antoinette AI - Violeau, Louis | 2020 | How Internalised Stigma Reduces Self-Esteem In Schizophrenia: The Crucial Role Of Off-Line Metacognition. | Western Europe | | Cross-Sectional | | Psychosocial |  |
| Von Mach, Tara, Rodriguez, Katrina, Mojtabai, Ramin, Spivak, Stanislav, Eaton, William W, Cullen, Bernadette A AI | 2020 | The Relationship Between Social And Environmental Factors And Symptom Severity In The Seriously Mentally Ill Population. | Northern America | | Longitudinal | | Health |  |
| Vrbová, K., Kamarádová, D., Látalová, K., Ocisková, M., Prasko, J., Mainerová, B., ... & Tichackova, A | 2014 | Self-Stigma, Adherence And Discontinuation Of Medication In Patients With Psychotic Disorders-Cross-Sectional Study | Eastern Europe | | Cross-Sectional | | Service Use |  |
| Vrbova, K., Prasko, J., Ociskova, M., Holubova, M., Kantor, K., Kolek, A., ... & Slepecky, M | 2018 | Suicidality, Self-Stigma, Social Anxiety And Personality Traits In Stabilized Schizophrenia Patients-A Cross-Sectional Study | Eastern Europe | | Cross-Sectional | | Health |  |
| Vrbova, K., Prasko, J., Ociskova, M., Kamaradova, D., Marackova, M., Holubova, M., ... & Latalova, K | 2017 | Quality Of Life, Self-Stigma, And Hope In Schizophrenia Spectrum Disorders: A Cross-Sectional Study | Eastern Europe | | Cross-Sectional | | Psychosocial |  |
| Werner, P., Aviv, A., & Barak, Y. | 2008 | Self-Stigma, Self-Esteem And Age In Persons With Schizophrenia | Western Asia | | Cross-Sectional | | Psychosocial |  |
| Windell, D., & Norman, R. M. | 2013 | A Qualitative Analysis Of Influences On Recovery Following A First Episode Of Psychosis | Northern America | | Qualitative | | Psychosocial |  |
| Wong, Y. L. I.; Kong, D.; Tu, L. F.; Frasso, R.; | 2018 | My Bitterness Is Deeper Than The Ocean: Understanding Internalized Stigma From The Perspectives Of Persons With Schizophrenia And Their Family Caregivers | Eastern Asia | | Qualitative | | Economical |  |
| Wood, L., & Irons, C | 2017 | Experienced Stigma And Its Impacts In Psychosis: The Role Of Social Rank And External Shame | Northern Europe | | Cross-Sectional | | Health |  |
| Wood, L., Byrne, R., Burke, E., Enache, G., & Morrison, A. P | 2017 | The Impact Of Stigma On Emotional Distress And Recovery From Psychosis: The Mediatory Role Of Internalised Shame And Self-Esteem | Northern Europe | | Cross-Sectional | | Health |  |
| Wright, E. R., Wright, D. E., Perry, B. L., & Foote-Ardah, C. E | 2007 | Stigma And The Sexual Isolation Of People With Serious Mental Illness | Northern America | | Qualitative | | Psychosocial |  |
| Wynaden, D., Chapman, R., Orb, A., Mcgowan, S., Zeeman, Z., & Yeak, S. | 2005 | Factors That Influence Asian Communities' Access To Mental Health Care | Australia And New Zealand | | Qualitative | | Service Use |  |
| Xu, Z., Mayer, B., Müller, M., Heekeren, K., Theodoridou, A., Dvorsky, D., ... & Rüsch, N. | 2016 | Stigma And Suicidal Ideation Among Young People At Risk Of Psychosis After One Year | Western Europe | | Longitudinal | | Health |  |
| Xu, Z., Müller, M., Heekeren, K., Theodoridou, A., Dvorsky, D., Metzler, S., ... & Rüsch, N. | 2016 | Self-Labelling And Stigma As Predictors Of Attitudes Towards Help-Seeking Among People At Risk Of Psychosis: 1-Year Follow-Up | Western Europe | | Cohort | | Service Use |  |
| Xu, Z., Müller, M., Lay, B., Oexle, N., Drack, T., Bleiker, M., ... & Rüsch, N | 2018 | Involuntary Hospitalization, Stigma Stress And Suicidality: A Longitudinal Study | Western Europe | | Longitudinal | | Health |  |
| Yanos, P. T., Lysaker, P. H., & Roe, D. | 2010 | Internalized Stigma As A Barrier To Improvement In Vocational Functioning Among People With Schizophrenia-Spectrum Disorders | Northern America | | Complex Cross-Sectional | | Economical |  |
| Yanos, P. T., Roe, D., Markus, K., & Lysaker, P. H. | 2008 | Pathways Between Internalized Stigma And Outcomes Related To Recovery In Schizophrenia Spectrum Disorders | Northern America | | Complex Cross-Sectional | | Psychosocial |  |
| Yanos, P. T., West, M. L., Gonzales, L., Smith, S. M., Roe, D., & Lysaker, P. H. | 2012 | Change In Internalized Stigma And Social Functioning Among Persons Diagnosed With Severe Mental Illness | Northern America | | Longitudinal | | Psychosocial |  |
| Yarborough, Bobbi J.; Yarborough, Micah T.; Cavese, Julie C.; | 2020 | Factors That Hindered Care Seeking Among People With A First Diagnosis Of Psychosis | Northern America | | Qualitative | | Psychosocial |  |
| Yen, C. F., Chen, C. C., Lee, Y., Tang, T. C., Ko, C. H., & Yen, J. Y | 2009 | Association Between Quality Of Life And Self-Stigma, Insight, And Adverse Effects Of Medication In Patients With Depressive Disorders | Eastern Asia | | Cross-Sectional | | Psychosocial |  |
| Yen, Shang-Yu, Huang, Xuan-Yi, Chien, Ching-Hui, Brown, E.C., Tas, C., Can, H., Esen-Danaci, A., Brune, M., Chen, L.W., Chen, L.Y., Chou, H.C., Ch... | 2020 | The Self-Stigmatization Of Patients With Schizophrenia: A Phenomenological Study. | Eastern Asia | | Qualitative | | Psychosocial |  |
| Yildirim, T., & Kavak Budak, F. | 2020 | The Relationship Between Internalized Stigma And Loneliness In Patients With Schizophrenia | Western Asia | | Cross-Sectional | | Psychosocial |  |
| Yildirim, Tulay, Kavak Budak, Funda AI - Yildirim, Tulay | 2019 | The Relationship Between Internalized Stigma And Loneliness In Patients With Schizophrenia. | Western Asia | | Cross-Sectional | | Psychosocial |  |
| Yılmaz, E., & Okanlı, A | 2015 | The Effect Of Internalized Stigma On The Adherence To Treatment In Patients With Schizophrenia | Western Asia | | Cross-Sectional | | Health |  |
| Yoshimura, Y., Bakolis, I., & Henderson, C. | 2018 | Psychiatric Diagnosis And Other Predictors Of Experienced And Anticipated Workplace Discrimination And Concealment Of Mental Illness Among Mental Health Service Users In England | Northern Europe | | Cross-Sectional | | Economical |  |
| Young, D. K. W., & Ng, P. Y. N | 2016 | The Prevalence And Predictors Of Self-Stigma Of Individuals With Mental Health Illness In Two Chinese Cities | Eastern Asia | | Cross-Sectional | | Psychosocial |  |
| Yuksel, C.; Bingol, F.; Oflaz, F.; | 2013 | Stigma: The Cul-De-Sac Of The Double Bind' The Perspective Of Turkiye; A Phenomenological Study | Western Asia | | Qualitative | | Psychosocial |  |
| Zäske, H., Linden, M., Degner, D., Jockers-Scherübl, M., Klingberg, S., Klosterkötter, J., ... & Gaebel, W | 2019 | Stigma Experiences And Perceived Stigma In Patients With First-Episode Schizophrenia In The Course Of 1 Year After Their First In-Patient Treatment | Western Europe | | Longitudinal | | Psychosocial |  |
| Zhang, T. M., Wong, I. Y. L., Yu, Y. H., Ni, S. G., He, X. S., Bacon-Shone, J., ... & Ran, M. S | 2019 | An Integrative Model Of Internalized Stigma And Recovery-Related Outcomes Among People Diagnosed With Schizophrenia In Rural China | Eastern Asia | | Complex Cross-Sectional | | Psychosocial |  |


# Types of stigma definitions

Experienced stigma**:** Stigma Scale of Explanatory Model Interview Catalogue; ; experienced stigma subscale of The Semi-structured Interview Measure of Stigma (SIMS); The overall effect of stigma and discrimination on the lives of patients (bespoke questionnaire); How much discrimination or unfair treatment did you experience because of your health problems during the past 30 days? (none, a little, some, a lot, or extreme unfair treatment); questionnaire that measure perceived rejection experiences; Multifaceted Stigma Experiences Scale (MSES); asking whether respondents were excluded from family decision compared with other household members of the same generation; whether the condition made it difficult to change jobs, made it difficult to advance in their current job, or ever caused them to lose a job; rejection experiences; The Explanatory Model Interview Catalogue Stigma Scale; The stigma and discrimination questionnaire; Personal experiences of discrimination included feeling mistrusted, rejected, or treated differently because of having a mental illness; “Do you believe that you’ve had trouble because you are a mental health consumer?”

Personal experiences of mental illness stigma subscale of The Stigma of Psychiatric Illness and Sexuality among Women (SPISEW); Inventory of Stigmatizing Experiences (ISE); Stigma subscale of Consumer Experiences of Stigma Questionnaire (CESQ); asking respondents the following question: "During the last six months, do you feel you have been discriminated against or stigmatized because of your mental illness diagnosis?”; The Stigma Scale; extent of embarrassment (stigma) people experienced due to their health condition; Burden due to stigma experiences scale

Perceived stgima**:** perceived subscale of The Semi-structured Interview Measure of Stigma (SIMS), Perceived Psychiatric Stigma Scale; Perceived Devaluation and Discrimination Scale; modified from the 5-item Stigma Scale for Receiving Psychological Help (SSRPH); The Personal Beliefs about Experiences Questionnaire (PBEQ); rate the degree of discrimination/stigma they face; 10-item Self-Concurrence/Application subscale of Self-Stigma of Mental Illness Scale.; stigmatising attitudes and beliefs; stereotype awareness subscale of The Chinese Self-stigma of Mental Illness Scale (CSSMIS); Link's scale of perceived stigma; The Perceived Stigma Scale; Devaluation of Consumers and Devaluation of Consumer Families Scales (DCS and DCFS); perceptions of social attitudes and behaviors toward mentally ill; Multidimensional Perceived Discrimination Scale;

Self-stigma**:** Internalized stigma of mental illness scale (ISMI), Stigma scale of Personal Beliefs about Illness Questionnaire (PBIQ); The Self-Stigma of Mental Illness (SSMI); Self-Stigma Scale-Short Form (SSS-S); The Self-Stigma Scale; The Self-Stigmatization Questionnaire; Self-concurrence and Self -esteem Decrement of the Self-stigma of Mental Illness Scale; disclosure subscale of the Stigma Scale (SS); Stereotype agreement, self-concurrence, and self-esteem decrement subscales of The Chinese Self-stigma of Mental Illness Scale (CSSMIS); personal stigma (“Social Impact” scale); “Would you be embarrassed or ashamed if you had a mental health problem?"

Help-seeking stigma: Stigma-related concerns about mental health care; Perceived stigma toward seeking mental health care

Stigma stress**:** Stigma Stress Scale

# Quantitative studies (full synthesis*)

## Health consequences

 The health domain included the next outcomes and their measurements:

- Depression was measured by: Hamilton Rating Scale for Depression; Center for Epidemiological Studies-Depression scale (CES-D); SCL-90-R depression subscale; PHQ-9; DSSS; Beck Depression Index (BDI); Beck Depression Inventory-Primary Care; Calgary Depression Scale (CDS); Calgary Depression Scale for Schizophrenia (CDSS); PANSS depression scores; Depression Anxiety Stress Scale (Depression subscale), Depression subscale of BPRS; Depression Anxiety Stress Scales
- Anxiety: Depression Anxiety Stress Scale (Anxiety scale), Beck Anxiety Index,  Generalised Anxiety Disorder 7-item Scale, m Spielberger State Trait Anxiety Inventory, Social Interaction Anxiety Scale (SIAS). Penn State Worry Questionnaire (PSWQ Panic and Agoraphobia Scale (PAS) Dimensional Obsessive-Compulsive Scale (DOCS); anxiety subscale of BPRS; GAD-7 scale; Social Interaction Anxiety Scale; Liebowitz Social Anxiety Scale;  Multidimensional Anxiety Questionnaire
- PTSD: Cumulative Trauma Disorder Scale; The Post-Traumatic Diagnostic Scale
- Psychotic symptoms: Psychosis subscale of the Colorado Symptom Index; DSM-4 assessment; Psychotic Symptom Rating Scales
- Transition to SZ
- Symptom severity: Brief Psychiatric Rating Scale BPRS; Colorado Client Assessment Record (CCAR); Hopkins Symptom Checklist HSCL; Positive and Negative Syndrome Scale (PANSS), Brief Symptom Inventory 18 (BPI); Colorado Symptom Index Prorodromal Questionnaire–Brief version (PQ-B)
- Recovery process: The Process of Recovery Questionnaire, Clinical Global Improvement Scale; Clinical Global Impression (CGI); Behavior and Symptom Identification Scale;
- Eating disorder: The Eating Disorder Examination Questionnaire (EDE-Q)
- Substance use: AUDIT, self-reported alcohol and tobacco use, medical history data
- Poor physical health: Physical health rating 1 to 5; Health Questionnaire; *describe your own overall physical and mental health during the past 30 days*
- Suicidality: suicidal attempt, suicidal index from MINI, suicidal ideation, suicidal risk, self-harm/suicidal thoughts, Suicide Behaviours Questionnaire (SBQ)
- Number of hospitalizations
- Number of outpatient visits
- Duration of illness
- Comorbidity


### Cross-sectional data

*Experienced stigma* was positively associated with depressive symptoms in four studies,^1–4^ and psychiatric symptoms severity in two studies.^4,5^ There were other positive associations found with psychotic symptoms,^6^ and personal recovery.^3,7^

Experienced stigma was also associated with poorer self-rated physical health.^8,9^ Additionally, it was associated with psychiatric hospitalizations.^10,11^ There was no evidence of association with duration of illness.^12^

Another study demonstrated that stigma mediates the effect of disability on perceived health.^13^ Self-esteem, and external shame were found to be significant mediators in the relationships between experienced stigma and depression.^3^

*Perceived stigma*

Another ten studies demonstrated a positive link between perceived stigma and depressive symptoms.^3,14–23^ Other positive associations were found with psychiatric symptoms,^9,24^ duration of illness,^25^ suicidality,^26,27^ social anxiety,^28^ and poor personal recovery.^3^ One study found a negative association of perceived stigma and severity of social anxiety and no association with duration of illness and number of episodes.^29^ Another study did not find association between perceived stigma and suicide attempts or number of affective episodes.^30^ There was also no evidence of association of perceived stigma number of hospital admission.^31^

Perceived stigma was shown to be mediating the relationship between level of insight and depressive symptoms,^32^ as well as moderating it.^33^ Another SEM analysis also demonstrated the effect of perceived stigma on depressive symptoms and self-esteem.^20^

*Self-stigma*

Nineteen studies demonstrated positive association of self-stigma with depressive symptoms and one study showed negative association,^34^ another no significant association in patients with schizophrenia.^35^ Number of depressive episodes was also shown not to be associated with the level of self-stigma.^36^ Eight studies demonstrated a positive association of self-stigma with anxiety symptoms.^14,37–43^ Higher self-stigma was also associated with having psychotic symptoms,^44^ PTSD symptoms,^37,43,45^ substance use and other self-reported illnesses,^46^ manic symptoms,^47^ mental comorbidities,^48^ and ADHD symptoms.^49^ Three studies did not find significant association between self-stigma and severity of psychiatric symptoms.^50,51^ Self-stigma was also shown to be associated with suicidal ideations,^52–54^ and suicide attempts.^44^ Thirteen studies demonstrated positive association of self-stigma with worse psychiatric symptoms (negative, positive or other symptoms).^35,48,49,55–64^

Another 10 studies displayed negative association of self-stigma with recovery from mental illness.^40,65–73^ Further six studies identified association of self-stigma with the number of psychiatric hospitalizations and two with the number outpatient visits.^67,71^ Self-stigma was positively associated with duration of mental illness in five studies and negatively in one study.^48^ There was no association found in one study with duration of illness or with number of hospitalizations.^70^ Lysaker et al (2012) also did not find association with number of hospital admission.^74^ Self-reported physical health was negatively associated with internalized stigma of mental illness in one study.^75^

Self-stigma was shown to be mediating the relationship between level of insight and depressive symptoms,^32,76^  and between quality of life and clinical symptoms.^68^ A PA demonstrated that self-stigma directly predicts depressive symptoms, anxiety and PTSD symptoms.^37^ Higher stigma was associated with higher levels of depression in a SEM model showing that self-stigma is a mediator between resilience and depression.^77^ In another study model self-stigma had a positive effect on depression and a negative effect on hope.^78^

Another three studies demonstrated a PA effect of stigma on symptoms severity through greater social avoidance.^35,79,80^ Two studies used SEM showing an effect of self-stigma on depressive symptoms,^81^ and anxiety.^42^ Another two studies demonstrated in a SEM analysis the mediating role of stigma on the relationship between family expressed emotions and recovery from mental illness,^56^ and between illness perceptions and recovery.^66^ Another study also demonstrated that effect of self-stigma on suicidality is mediated by hopelessness.^82^

It was also demonstrated that the link between perceived stigma and suicidality is mediated by secrecy and hopelessness.^26^

### Longitudinal data

Longitudinal studies demonstrate that self-stigma,^83,84^ and experienced stigma at baseline each were associated with higher depressive symptoms at follow-up.^12^ Similarly, self-stigma at baseline was associated with higher psychotic symptoms at follow-up.^83^ Another study demonstrated that reduction in self-stigma scores during the follow-up period was associated with reduction in depressive and anxiety symptoms and clinical recovery.^75^ It was shown as well that stigma stress is associated with poor recovery at 2 year follow-up.^85^ Conversely, perceived stigma was not shown to be a significant predictor of depression at 3 months follow-up.^86^

It was also demonstrated that psychotic symptom severity proved to be both a predictor and an outcome of intensification in self-stigmatizing beliefs.^87^ An increase of stigma stress score was associated with suicidality after one year of follow-up.^27^ Greater self-stigma associated with mental disorder at baseline also predicted suicidal ideation after 1 year but not after 2 years.^88^

Stigma experiences have been shown to have a small adverse effect on psychiatric symptoms and life satisfaction across the 18-month interval.^89^ Additionally, baseline levels of stereotype endorsement were associated with social anxiety at baseline and at 5-months follow up.^90^ Higher levels of self-stigma at baseline were significantly associated with more positive symptoms and lower levels of role functioning after 12 months,^91^ and with negative symptoms, namely emotional discomfort,^92^ or psychiatric hospitalization at six months follow-up.^93^ Conversely perceived stigma was not shown to be predictive of affective relapses at 24 month follow up.^94^ Ilic and colleagues showed in a cohort study a causal relationship between the presence of stigma experiences and worse mental health measured by severity of psychiatric symptoms.^95^ In a survival analysis by Rusch et al., stigma stress was shown to be increasing the risks for transitioning to schizophrenia among young people at risk for psychosis.^96^

## Personal consequences

 The personal domain included the next outcomes and their measurements:

- Negative attitudes to treatment were assessed by Attitudes toward mental health services scale,
- Attitudes toward mental health treatment; Drug Attitude Inventory, hope for cure with medication; attitudes toward psychotherapy.
- Satisfaction with services: satisfaction with services scale; number of unmet service needs.
- Adherence to treatment: Morisky (Motisky-Green) medication adherence questionnaire; Rating of Medical Influence (ROMI); Psychosocial Treatment Compliance Scale; Medication Adherence Rating Scale; adherence to subsequent treatment; discontinuation of medication; regular medication use; self-reported frequency and pattern of missed doses and whether patients had stopped taking their medication completely.
- Therapeutic alliance: Working Alliance Inventory-Short Form; Scale to Assess the Therapeutic Relationship–Patients Version.
- Attitudes to illness: susceptibility subscale of illness concepts Scale; Mishel Uncertainty in Illness Scale.
- Lower help-seeking: having been treated previously, The barriers to care checklist, Attitudes toward seeking counseling, intention toward seeking mental health services, Willingness to seek psychological help; The General Help-Seeking Questionnaire; current services utilization
- Recovery orientation: The Mental Health Recovery Scale; Maryland Assessment of Recovery in Serious Mental Illness Scale; Stages of Recovery Scale; Recovery Assessment scale; Recovery Style Questionnaire; Stages of Recovery Instrument (STORI); The Questionnaire about the Process of Recovery
- Well-being: Profile of Mood States (POMS); Subjective Wellbeing Under Neuroleptic Treatment; Positive and Negative Affect Scale; Ryff’s Scales of Psychological Well-Being; Psychological wellbeing scale
- Quality of life: Lehman's measure of life quality; Schizophrenia Quality of Life Scale; WHO Quality of Life-Bref; Wisconsin Quality of Life index; Health-related quality-of-life score 5-level EQ-5D; Quality of Life Satisfaction and Enjoyment Questionnaire; Multidimensional Students’ Life Satisfaction Scale–MSLSS; Q-LES-Q-SUM; Health Survey - 12 item short form
- Psychological distress: Global Severity Index; General Health Questionnaire -12; Emotional Discomfort component of PANSS
- Self-esteem: Rosenberg Self-Esteem Scale; Multidimensional self-esteem inventory; The Success and Resource Appraisals Questionnaire; Self-Esteem Rating Scale
- Self-efficacy: Pearlin’s Mastery Scale; why-try effect; Self-efficacy Scale
- Empowerment: Empowerment-Making Decisions; Empowerment Scale
- Hope: The Beck Hopelessness Scale; Herth Hope scale; Adult Dispositional Hope Scale; Domain-Specific Hope Scale
- Experienced discrimination: DISC
- Shame: Internalised Shame Scale, The Other as Shamer (OAS) Scale

Table 3. Distribution by region and level of evidence - personal consequences*

| Outcome | Northern America | | | South America | | | Eastern Asia | | | Southern Asia | | | Western Asia | | | South-eastern Asia | | | Australia and N Zealand | | | Eastern Europe | | | Southern Europe | | | Northern Europe | | | Western Europe | | | International | | | Eastern Africa | | | Western Africa | | |
| --- | --- | --- | --- | --- | --- | --- | --- | --- | --- | --- | --- | --- | --- | --- | --- | --- | --- | --- | --- | --- | --- | --- | --- | --- | --- | --- | --- | --- | --- | --- | --- | --- | --- | --- | --- | --- | --- | --- | --- | --- | --- | --- |
|  | A | B | C | A | B | C | A | B | C | A | B | C | A | B | C | A | B | C | A | B | C | A | B | C | A | B | C | A | B | C | A | B | C | A | B | C | A | B | C | A | B | C |
| Negative attitudes to treatment | 2 |  |  |  |  |  |  |  |  | 1 |  |  | 1 |  |  |  |  |  |  |  |  | 5 |  |  |  |  |  |  |  |  |  | 1 |  |  |  |  |  |  |  |  |  |  |
| Satisfaction with services | 2 |  | 1 |  |  |  | 1 |  |  |  |  |  |  |  |  |  |  |  |  |  |  |  |  |  |  |  |  |  |  |  |  |  |  |  |  |  |  |  |  |  |  |  |
| Treatment adherence | 4 |  |  | 1 |  |  | 3 | 1 |  |  |  |  | 1 |  |  |  |  |  |  |  |  | 1 |  |  |  |  |  | 1 |  |  | 1 |  |  |  |  |  | 1 |  |  | 1 |  |  |
| Therapeutic alliance |  |  |  |  |  |  | 1 |  |  |  |  |  |  |  |  |  |  |  |  |  |  |  |  |  |  |  |  |  |  |  | 1 |  |  |  |  |  |  |  |  |  |  |  |
| Attitudes to illness |  |  |  |  |  |  | 1 |  |  |  |  |  |  |  |  |  |  |  |  |  |  | 1 |  |  |  |  |  |  |  |  |  |  |  |  |  |  |  |  |  |  |  |  |
| Expressed emotions |  |  |  |  |  |  | 2 | 1 |  | 2 |  |  |  |  |  |  |  |  |  |  |  |  |  |  |  |  |  |  |  |  |  |  |  |  |  |  |  |  |  |  |  |  |
| Non-disclousre |  |  |  |  |  |  | 1 |  |  |  |  |  |  |  |  |  |  |  |  |  |  |  |  |  |  |  |  | 1 |  |  |  |  |  |  |  |  |  |  |  |  |  |  |
| Help-seeking | 12 |  | 3 |  |  |  |  | 1 |  | 1 |  |  |  |  |  |  |  |  |  | 1 |  |  |  |  |  |  |  |  | 1 |  | 1 |  |  | 1 |  |  |  |  |  |  |  |  |
| Recovery orientation | 2 | 2 |  |  |  |  | 1 | 2 |  | 1 |  |  |  |  |  |  |  |  |  |  |  |  |  |  | 2 |  |  |  |  | 1 |  |  | 1 |  |  |  |  |  |  |  |  |  |
| Self-esteem | 9 | 5 | 1 |  |  |  | 8 | 2 |  | 2 |  |  | 3 | 2 |  | 1 |  |  |  |  |  |  |  |  | 2 | 3 |  | 4 | 1 | 1 | 5 |  | 2 | 1 |  |  |  |  |  |  |  |  |
| Quality of life | 4 | 2 | 4 | 1 |  |  | 17 | 4 |  | 1 |  |  | 1 | 1 |  |  |  |  |  |  |  | 5 |  |  | 2 | 3 |  | 1 |  |  | 6 | 1 |  | 2 |  |  |  |  |  | 1 |  |  |
| Well-being | 2 |  |  |  |  |  |  |  |  |  |  |  |  |  |  |  |  |  |  |  |  |  |  |  | 2 | 1 |  | 1 |  |  | 1 |  | 1 |  |  |  |  |  |  |  |  |  |
| Self-efficacy | 4 | 2 |  |  |  |  | 1 |  |  |  |  |  |  |  |  |  |  |  |  |  |  |  |  |  |  | 1 |  |  |  |  | 2 | 1 |  |  |  |  |  |  |  |  |  |  |
| Empowerment | 1 |  |  |  |  |  | 1 |  |  |  |  |  |  |  |  |  |  |  |  |  |  |  |  |  |  |  |  | 3 |  |  |  | 1 |  |  |  |  |  |  |  |  |  |  |
| Hopelessness |  | 2 |  |  |  |  |  | 2 |  |  |  |  | 1 | 1 |  |  |  |  |  |  |  | 1 |  |  | 1 |  |  | 1 | 1 | 2 |  | 1 |  |  |  |  |  |  |  |  |  |  |
| Shame |  |  |  |  |  |  |  |  |  |  |  |  |  |  |  |  |  |  |  |  |  |  |  |  |  |  |  | 2 |  |  |  |  |  |  |  |  |  |  |  |  |  |  |
| Total | 42 | 13 | 9 | 2 |  |  | 37 | 13 |  | 8 |  |  | 7 | 4 |  | 1 |  |  |  | 1 |  | 13 |  |  | 9 | 8 |  | 14 | 3 | 4 | 17 | 5 | 4 | 4 |  |  | 1 |  |  | 2 |  |  |

      A – cross-sectional design. B – cross-sectional design with complex analysis, C – longitudinal design

### Cross-sectional evidence

We found 18 studies where participants who have mental disorders reported stigma being a reason for not seeking help or for delaying help-seeking for mental health problem and two studies where stigma was reported as a reason for either dropping out of treatment,^97^ or non-adherence to treatment.^98^

*Self-stigma*

Eight studies displayed association of self-stigma with negative attitudes to treatment.^46,67,71,99–103^

Higher self-stigma was associated with medication non-adherence in another nine studies. . Conversely, a Taiwanese study failed to find evidence of association between self-stigma and medical compliance.^104^ Self-stigma was shown to have negative association with establishing good therapeutic alliance in two studies and also to mediate the negative impact of symptom severity on therapeutic alliance.^60,105^ It was also positively associated with uncertainty about illness.^55^ Higher self-stigma was also associated with non-disclosure of mental illness,^106^ reduced empowerment,^36,55,107,108^ experienced discrimination,^106,109^ and higher symptom distress.^41,110^ SEM analysis confirmed that poor social capital contributes to increased self-stigma and indirectly.^36^ Four American studies displayed that self-stigma was significantly associated with lower help-seeking.^99,111–113^ In India, lower stigma was associated with informal help-seeking.^114^

Furthermore, nine studies showed that self-stigma is negatively associated with subjective recovery from mental illness. Recovery style itself also moderated the stigma effect on depression.^115^ The effect of self-stigma on baseline recovery judgements appeared to be mediated through low self-esteem and hopelessness, but there was no mediation at 6-months follow-up.^116^ Self-stigma was associated with less satisfaction with mental health services,^117^ and with lower well-being.^118–120^ It was also mediating the association of perceived discrimination and well-being.^121^ Twenty four studies identified negative association between internalized stigma and self-esteem, two studies found positive association and one study did not identify significant association with self-esteem. With regard to self-efficacy, four studies demonstrated negative association.^81,122–124^ Forty two studies found negative association of self-stigma with quality of life and ten studies demonstrated positive association with hopelessness or negative association with hopefulness. One study demonstrated that self-stigma did not have a significant association with quality of life.^125^

In a Chinese PA study self-stigma was shown to have direct and indirect effect on psychosocial treatment adherence.^126^ Another PA model demonstrated that self-stigma mediates the effect of PTSD symptoms on intention to seek help.^45^

It was also demonstrated in a SEM analysis that self-stigma mediates the effect of expressed emotions,^56^ and illness perceptions on recovery and that self-stigma, self-esteem and self-efficacy mediate effect of experienced stigma on recovery,^66^ quality of life and social avoidance.^127^

Effect of stigma on treatment attitudes was also mediated by self-efficacy in one study.^42^ Another two studies showed the effect of self-stigma on self-esteem and hope, which in turn affected quality of life.^128,129^ Similar path was demonstrated in another study, which in addition led to increased suicide ideation in people with schizophrenia.^130^ Another study showed mediation of hope and self-esteem on the path between self-stigma and lower quality of life,^131^ and between self-stigma and social avoidance and depression.^35^ One study in a PA showed the effect of self-stigma on self-esteem and self-efficacy, which in turn affect well-being,^129^ and on subjective recovery.^132^ A SEM analysis showed that self-stigma is linked to poor sleep and consequently poorer quality of life.^133^

*Perceived stigma*

Lower perceived stigma was shown to be associated with better adherence to medication treatment,^134,135^ and with attendance to psychosocial treatment programmes.^122^ This was also true for older patients in one study.^135^ It was also shown that caregivers’ stigma significantly predicted patients’ decreased adherence.^136^ Perceived stigma had a negative association with perceived working alliance with a doctor,^137^ and positive association with the number of unmet needs.^138^ It was also associated with more symptom distress.^110^ Higher values of perceived stigma were also associated with the belief that illness is a threat for oneself.^7^ Additionally, perceived stigma was shown to be associated with lower help-seeking in six studies. Conversely, four studies did not find significant association between perceived stigma and seeking psychological help and treatment drop-out.^139–142^

Perceived stigma was also associated with hopelessness,^5^ lower subjective mental health recovery,^110^ poor recovery expectations.^143^ One study showed that the relationship between perceived public stigma and negative attitudes to treatment is mediated by internalized stigma.^144^ Fifteen studies showed a negative association between perceived stigma and self-esteem. Perceived public stigma was associated with why-try effect.^145^ Twelve studies demonstrated negative association of perceived stigma with quality of life and two studies with empowerment,^10,21^ and lower well-being.^23,121,146,147^ One study failed to demonstrate evidence of correlation of stigma with self-esteem, well-being and self-acceptance.^148^

A PA demonstrated the direct influence of perceived public stigma on willingness to seek help, which was mediated by self-stigma and attitudes toward help seeking.^137^ A SEM study showed that participants who adopted secrecy (i.e. non-disclosure) and withdrawal coping orientations had higher levels of self-stigma, which in turn worsened the effects of societal stigma on mental health recovery.^110^ Another SEM demonstrated that self-efficacy and empowerment mediated psychological effects of perceived stigma on depressive symptoms and quality of life.^21^ In addition, a SEM model showed that stigmatization was negatively associated with self-esteem and with social anxiety.^29^ Other SEM analyses also provided evidence for the effect of perceived stigma on self-esteem.^20,33^ Perceived stigma’s effect on quality of life was mediated through the intensity of depressive symptoms and presence of social support.^15^ One study found in a SEM analysis the moderating and mediating role of self-stigma in the association between insight about mental illness and demoralization, however follow-up results did not support the baseline evidence.^149^

*Experienced stigma*

Experienced stigma was shown to be associated with non-adherence.^150^ One study demonstrated that as level of experienced discrimination increases, help‐seeking delay also increases at an exponential rate.^151^ Two studies showed a negative association with quality of life.^1,152^ Experienced stigma also was found to be moderating the relationship between insight and recovery orientation.^153^ It was also associated with hopelessness,^3^ psychological distress,^1^ and in four studies with lower self-esteem.^3,154-156^ Two studies demonstrated its relationship with reduced empowerment,^156,157^ and two with internalized shame.^3,4^

### Longitudinal evidence

Increase in perceived stigma after 1 one year of follow-up predicted more negative attitudes toward psychotherapy.^158^ In another study, it was demonstrated that stigma endorsement in people with depression predicted less help seeking at 6-months follow-up.^159^ However, a taiwanese study demonstrated that self-stigma at baseline did not predict severity of depression symptoms, suicide risk or medication adherence at 1 year follow-up.^160^

More self-stigma at baseline predicted less recovery orientation at baseline and at 1 year after controlling for symptom severity, age and gender, however these results did not hold after two years.^161^ A decrease of stigma stress predicted better well-being at 1 year follow-up,^162^ and an increase of self-stigma predicted higher levels of demoralization at 12 months follow-up.^91^ Perceived stigma at baseline predicted lower self-esteem at 6 months follow-up after controlling for baseline self-esteem.^163^ The link between self-stigma at 1-year follow-up and suicidal ideation after 2 years was mediated by decreased self-esteem at 1-year follow-up.^164^

Reduction in self-stigmatizing beliefs over a follow-up period was associated with improvement in functioning and quality of life,^75^ and less emotional discomfort at six months,^92^ however in another longitudinal analysis self-stigma was not proven to be a significant predictor of quality of life.^165^ Similarly there was no evidence that less self-stigma predicts better quality of life at six months.^166^ Adverse effects of experienced stigma were shown also to have an effect in changes in symptoms and quality of life at 18 months follow up, but not self-esteem.^167^ Although concurrently stereotype endorsement was related to emotional distress, there was no association in the longitudinal design.^74^

One study identified a relationship between stigma experiences and lower self-esteem after 1 year but the sample size was too small.^7^

## Economic consequences

*Self-stigma*

Eight studies demonstrated that higher self-stigma is associated with being unemployed. Three studies did not find association with employment.^36,57,168^ Additionally, two studies found that self-stigma is associated with lower income.^55,61^

*Experienced stigma*

Experienced stigma was shown to be associated with financial difficulties.^169^ THere were no differences in experienced stigma found between employed and unemployed participants.^11^

*Perceived stigma*

In a large multinational study (n=8796) perceived stigma was significantly associated with being unemployed.^170^ It was also associated with lower family income among people with depression.^2^ Two studies demonstrated that perceived stigma was not related to employment status.^23,31^

## Psychosocial consequences

 The psychosocial domain included the next outcomes and their measurements:

- - Avoidance: Cognitive Behavioral Avoidance Scale (CBAS), isolation from relatives; Social engagement/withdrawal subscale score from the Social Functioning Scale.
  - Disability: WHODAS, being on disability benefit
  - Expressed emotions: level of Expressed Emotion (LEE) Scale.
  - Functioning: WHODAS-2.0 and Butajira Functioning Scale; Global Assessment of Functioning; Specific Level of Functioning; Social and Occupational Functioning Scale; Functional Recovery Scale in Schizophrenia; Functional remission of general schizophrenia scale, Social Functioning Scale; Weiss Functional Impairment Rating Scale; The Functioning Assessment Short Test FAST; Camberwell Assessment of Need; The Quality of Life Scale;
  - Social activity: Indicators of social network size and reciprocity (Social Relationship Scale) and social contact (three items from the Social Inclusion Scale) as well Community belonging (cultural inclusion); Bespoke instrument with time budget and schedule for the assessment of activity; Work days lost index; Social limitation index
  - Social capital: Number of friends or relatives; UCLA- Loneliness scale; long-term friendship; Resource Generator-UK (participants’ access to social resources within their own social network)
  - Social rank: the social comparison scale
  - Social support: Social Support List-Interactions; Social Support Rating Scale; The Duke Social Support Index; The Interpersonal Support Evaluation List; Social Support Scale-Friend

### Cross-sectional evidence

*Self-stigma*

It was shown that higher self-stigmatization is associated with social avoidance,^35,79,171^ being on disability pension,^172^ and with higher level of familial expressed emotions.^56,173^ It was also associated with lower education in ten studies and with higher education in two studies. Self-stigma was positively associated with psychosocial functioning impairment,^49,80,174^ negatively with functional restoration,^56,175^ and mediating the relationship between perceived stigma and functioning.^176^ It also predicted reduced social activity.^177^ Higher self-stigma was associated to being single in three studies,^34,44,178^ and not associated with marital status in seven, including when symptom severity was controlled for.^23^ It was associated with lower relational satisfaction and fear of relationship in people with bipolar disorder,^179^ and with poor parenting experience.^180^ Higher levels of self-stigma were associated with reduced social capital in four studies: lower number of friends and relatives and perceived quality of support,^181^ loneliness,^182,183^ and with absence of long-term friendships.^46^ It was also demonstrated that reduced self-stigma is associated with increased number of social contacts and higher social capital.^36,107,108,^ Self-stigma was negatively associated with perceived social support.^80,184,185^ Availability of social support services predicted higher self-stigma.^51^

*Experienced stigma*

Experienced stigma was associated with social withdrawal,^127^ worse sense of coherence,^156^ and higher level of expressed emotions.^5^ Higher values of rejection and discrimination were associated with higher family burden and level of familial expressed emotions.^138^ It was also associated with being on disability benefit.^156^ Severe effects of stigma were associated with higher education.^5^ However another study did not show a difference in level of stigma experiences between education groups.^11^ Higher stigma experiences were also associated with worse functioning.^2,156^ It has also been shown that higher experienced stigma was associated with lower perceived social support.^154^ Greater experience of stigma significantly increased the odds of engaging in risky sexual behaviour as shown in one study.^186^ Social rank was found to be a mediator between experienced stigma and recovery.^4^

*Perceived stigma*

Higher perceived family stigma was associated with suspiciousness and inappropriate behavior.^114^ Perceived stigma was also negatively associated with functionality.^138,176,187,188^  Additionally, it was associated with low education and being married/having a partner.^170^ Three studies did not show association with education time.^23,29,31,^ In another study, perceived community microaggressions were associated with less participation in community.^189^ Perceived stigma was associated with higher work and social limitation.^170^ One study demonstrated a negative association between perceived stigma and perceived social support.^15^

### Longitudinal evidence

A study has shown that self-stigma predicts social withdrawal, guilt feelings and self-blame at six months follow-up.^116^ In addition, change in self-stigma was significantly negatively related to change in social functioning, controlling for negative symptoms at 4 and 7 months follow-up.^190^ Reduction in self-stigma positively predicted changes in the community living skills of patients with affective disorders over a 12-month period.^191^ Patients with bipolar disorder with stigma concerns showed more impairment in social adjustment at 7 months follow-up after symptom severity and social functioning at baseline were controlled.^192^ Greater self-stigma also was shown to be associated with reduced social inclusion and vocational activity at 5-month follow-up and this appeared to be mediated by hopelessness.^193^

In an experimental study significantly less fictitious candidates with reported mental illness history received callbacks then candidates with physical illness history.^194^ However, in another experiment, stigma effect of year inactivity  due to depression in a job application was no different from the effect of year inactivity due to unexplained reason.^195^

Table 4. Distribution by region and level of evidence - psychosocial and economic consequences

| ^Outcome^ | ^Northern America^ | | | ^South America^ | | | ^Eastern Asia^ | | | ^Southern Asia^ | | | ^Western Asia^ | | | ^South-eastern Asia^ | | | ^Eastern Europe^ | | | ^Southern Europe^ | | | ^Northern Europe^ | | | ^Western Europe^ | | | ^International^ | | | ^Eastern Africa^ | | | ^Western Africa^ | | |
| --- | --- | --- | --- | --- | --- | --- | --- | --- | --- | --- | --- | --- | --- | --- | --- | --- | --- | --- | --- | --- | --- | --- | --- | --- | --- | --- | --- | --- | --- | --- | --- | --- | --- | --- | --- | --- | --- | --- | --- |
|  | ^A^ | ^B^ | ^C^ | ^A^ | ^B^ | ^C^ | ^A^ | ^B^ | ^C^ | ^A^ | ^B^ | ^C^ | ^A^ | ^B^ | ^C^ | ^A^ | ^B^ | ^C^ | ^A^ | ^B^ | ^C^ | ^A^ | ^B^ | ^C^ | ^A^ | ^B^ | ^C^ | ^A^ | ^B^ | ^C^ | ^A^ | ^B^ | ^C^ | ^A^ | ^B^ | ^C^ | ^A^ | ^B^ | ^C^ |
| ^Unemployment^ | ^1^ |  |  |  |  |  | ^2^ |  |  | ^1^ |  |  |  |  |  | ^1^ |  |  | ^1^ |  |  |  |  |  |  |  |  | ^1^ |  |  | ^3^ |  |  |  |  |  | ^1^ |  |  |
| ^Low income, povery, financ. issues^ | ^2^ |  |  |  |  |  | ^1^ |  |  | ^1^ |  |  | ^1^ |  |  |  |  |  |  |  |  |  |  |  |  |  |  |  |  |  |  |  |  |  |  |  | ^1^ |  |  |
| ^Avoidance^ | ^2^ | ^2^ |  |  |  |  |  |  |  |  |  |  | ^1^ |  |  |  |  |  |  |  |  |  |  |  |  |  | ^1^ |  |  | ^1^ |  |  |  |  |  |  |  |  |  |
| ^Disability^ |  |  |  | ^1^ |  |  |  |  |  |  |  |  |  |  |  |  |  |  | ^1^ |  |  |  |  |  | ^1^ |  |  |  |  |  |  |  |  | ^1^ |  |  |  |  |  |
| ^Expressed emotions^ |  |  |  | ^2^ |  |  | ^2^ | ^1^ |  | ^1^ |  |  |  |  |  |  |  |  |  |  |  |  |  |  |  |  |  |  |  |  |  |  |  | ^1^ |  |  |  |  |  |
| ^Functioning^ |  |  | ^3^ |  |  |  | ^4^ |  | ^1^ | ^1^ |  |  | ^1^ |  |  |  |  |  |  |  |  | ^1^ | ^1^ |  | ^2^ |  |  | ^1^ |  | ^3^ | ^1^ |  |  |  |  |  |  |  |  |
| ^Social activity^ |  |  |  |  |  |  |  |  |  |  |  |  |  |  |  |  |  |  |  |  |  |  |  |  | ^1^ |  | ^1^ |  |  |  |  |  |  |  |  |  |  |  |  |
| ^Social capital^ | ^2^ |  |  |  |  |  |  |  |  |  |  |  | ^1^ |  |  |  | ^1^ |  |  |  |  |  |  |  | ^1^ |  |  |  |  |  |  |  |  |  |  |  |  |  |  |
| ^Social rank^ | ^2^ |  |  |  |  |  |  |  |  |  |  |  |  |  |  |  |  |  |  |  |  |  |  |  | ^1^ |  |  |  |  |  |  |  |  |  |  |  | ^1^ |  |  |
| ^Social support^ | ^2^ |  |  |  |  |  | ^1^ | ^2^ |  |  |  |  | ^1^ |  |  | ^1^ |  |  |  |  |  |  |  |  |  |  |  | ^1^ |  |  |  |  |  |  |  |  | ^1^ |  |  |
| ^Total^ | ^11^ | ^2^ | ^3^ | ^3^ |  |  | ^10^ | ^3^ | ^1^ | ^4^ |  |  | ^5^ |  |  | ^2^ | ^1^ |  | ^2^ |  |  | ^1^ | ^1^ |  | ^6^ |  |  | ^3^ |  | ^4^ | ^4^ |  |  | ^2^ |  |  | ^4^ |  |  |

        A – cross-sectional design. B – cross-sectional design with complex analysis, C – longitudinal design

# Qualitative studies (full synthesis*)

## Organizational consequences

The domain of organizational outcomes yielded 13 qualitative studies. The domain mostly described consequences or correlates pertaining to the legal area, media representation, service user involvement and availability of healthcare, with one study describing the impact of stigma on access to state benefits.

The first theme to appear within the organizational domain is that of legal issues. Six studies all together reported this theme. The studies came from different regions: Southern Europe, Western Africa and Southern Africa, Eastern Africa, Northern America, South-East Asia.

Some studies described laws as a stigmatizing consequence of stigma in society. Such laws often either deemed PWMI as not capable of deciding on their own or used derogatory and stigmatizing language. This was reported both from the experience of perceived and experienced stigma.^196–198^ Four studies informed on issues related to law regarding custody over children. Mental illness negative perceptions (often related to perceived dangerousness) were used in court as a reason for custody decisions against people with mental illness. Furthermore, a mental illness diagnosis was often perceived by participants as the reason for unfair treatment by child welfare services.^199–201^

The second theme was media and media representation of mental illness. Four studies reported on this theme. The studies were from Northern, Eastern, Southern and Western Europe. All of the studies reported from the perspective of perceived stigma.

All of the studies describe participant’s belief that public stigma fuels negative imagery in the media relating to mental illness. Furthermore, they believed that the media negative portrayal of mental illness was a reflection of public stigma which exists within society. Finally, they believed that negative representation of mental illness in the media in turn increased stigmatization.^197,202–204^

Impact of stigma on service user involvement was described as a theme by two studies. The studies were both from the Eastern Africa region.

Both studies describe stigma and discrimination as barriers to service user involvement in policy making and are both based in perceived and experienced stigma. Frequently, due to experiences of stigma PWMI are not considered as capable of making decisions. As a secondary consequence, PWMI are deterred from trying to impact change and or participate in policy making activities due to fear of not being heard or understood.^205,206^

Inadequate availability of healthcare and access to healthcare was also perceived as a consequence of stigma, this theme was reported in three studies. The studies were from Western Africa, Southern Africa and Western Europe.

Poor quality of mental healthcare services was perceived as the best example of structural stigma.^204^ Other studies described that stigma impacted funding decisions of mental healthcare, which led to care being inadequate for example not having enough medication or not having enough coverage of mental healthcare.^196,207^

Finally, one study from Northern Europe reported on the impacts of mental illness perceived and experienced stigma on accessing state benefits. Participants reported that the process in which state benefits for PWMI have to be obtained were deeply stigmatizing, for example, the language used in the forms suggested that PWMI are not capable of deciding or living on their own.^208^

## Health consequences

The domain describes consequences or correlates pertaining to the following key themes:  aggravation of diagnosed mental illness, other mental health outcomes and quality of obtained healthcare.

Four studies described the theme of aggravated symptoms, where the symptoms pertaining to the mental illness diagnosis of the patient were worsened as a consequence of stigma. Two of the studies were from Northern Europe and one from South East Asia.

While two studies described stigma as having a general impact on aggravating the mental health of participants.^209,210^ Two studies described the impact more specifically, reporting that stigma impacted experiences of psychosis with participants either hearing voices or having an increased sense of paranoia as a consequence to stigma.^86,203^

The appearance of other mental health outcomes as a consequence of stigma was described by two studies. The studies came from Northern Europe and from Southern Africa.

Burke et al. describe a general lower mood and unhappiness as a consequence of stigma.^203^ In Pyle et al.’s study participants described the impact of stigma as psychological distress which then led to anxiety and lowered mood.^86^

Finally, fourteen studies described the impact of stigma on participants' health due to stigmatized healthcare. The studies came from a wide range of regions: South America, Eastern Europe, Northern Europe, Northern America, Southern Europe, Australia and New Zealand.

In their study Lyons et al., (2009) show the experience of one participant with sub-optimal healthcare due to stigmatizing attitudes of healthcare staff resulting in further health complications.^211^ Similarly, Schulze et al.’s study participants describe healthcare professionals’ lack of interest in their history.^204^ Other studies describe instances of being treated differently, instances of diagnostic overshadowing and feeling excluded in the hospital setting as well as in primary care such as GP practices.^212–224^

One study described the impact of stigma on physical activity, where perceived negative attitudes of the public towards mental illness lowered participants' willingness to participate in leisure time physical activities.^225^

## Personal consequences

The domain describes consequences or correlates pertaining to the following key themes: treatment adherence, inhibited process of recovery, negative internal emotions, poor help-seeking, concealment and isolation, self-stigma.

Treatment adherence was described as a theme in eleven studies. Three studies were from Northern America, and the rest from Northern Europe, Australia and New Zealand and one was done in an international context.

One study defined the impact of stigma on the acceptance of illness and therefore the acceptance of treatment.^226^ The rest of the studies described the impact of stigma on adhering to medication intake, participants most often described feeling shame in relation to taking medication.^227–235^ Two studies also described parents of adolescents with mental illness not wanting their child to adhere to treatment due to stigma.^233,236^

Inhibited process recovery appeared as a theme in five studies. The studies were predominantly from Eastern and South Eastern Asia, Australia and New Zealand, Northern America and Northern Europe.

Experienced stigma was reported to generally inhibit or slow down the process of recovery in five studies.^203,226,237–239^ Participants in Windell et al.’s study described stigma as impacting confidence and self-esteem therefore impacting the process of recovery.^226^ Some studies reported only certain aspects of recovery such as decreased resilience or daily functioning being impacted by stigma.^203,240^

Negative internal emotions such as shame or lowered self-esteem were mentioned as a consequence of stigma by twenty-five studies. The studies came from the following regions: Eastern Asia, Northern America, Southern Asia and Australia.

Twelve studies described participants describing shame as a consequence of stigmatizing experiences. Shame and embarrassment most often stemmed from stigmatizing attitudes from friends and family.^203,214,235,237,241–247^  Eight studies described lowered self-esteem as a consequence of stigma. This was most often described as an internalization of public negative beliefs towards PWMI.^86,203,208,226,238,248–250^ One study also described self-doubt connected to lowered self-esteem.^251^ Finally, two studies described participants having feelings of hopelessness as a result of stigma.^221,252^

Fifty-three studies described reduced help seeking as a consequence of stigma. The studies came from the following regions: Eastern Asia, South East Asia, Northern America, Australia and New Zealand and Northern Europe.

Stigma in general impacted willingness to seek help, this was mentioned in twenty-three studies. Three studies described stigma leading to a reluctance to acknowledge mental illness and therefore seek help.^226,237,253,254^ Five studies described shame attached to seeking help.^244,255–258^ Twenty studies explored anticipated stigma as an impact to reduced help seeking. Finally, poor help seeking due to stigma was also perceived in caregivers of children with mental health disorders.^259,260^

Concealment of mental illness diagnosis and isolation from society were described as stigma consequences in forty-two studies. Most of the studies came from: Eastern Asia, South East Asia, Western Europe, Northern America and Eastern Europe.

Thirty studies describe concealment as a consequence of stigma. Generally participants decided to conceal their mental illness diagnosis in all situations due to anticipated stigma and discrimination. Some studies reported on concealment in front of family, or being asked by family members to conceal mental illness diagnosis due to shame on the side of family members.^209,222,232,261^ Finally, some studies describe participants concealing their mental illness in the work environment due to fear of receiving less work opportunities or being fired from work due to stigma.^200,211,261,262^ Isolation was described in fourteen studies, isolation in this case means the voluntary or involuntary removal from social situations and social interactions. Isolation being the results of anticipated stigma was reported in some studies while others assign isolation as an outcome of long-term stigmatizing experiences.

Self-stigma as a consequence of stigma in general was reported by five studies. The studies came from the following regions: Eastern Asia, Northern America, Australia and New Zealand, Southern Europe and Northern Europe.

Participants most often described internalizing stigmatizing messages which they perceived on a daily basis, often also feeling shame or a sense of failure.^197,237,241,263^

## Psychosocial consequences

The domain mostly describes consequences or correlates pertaining to the following key themes: experienced and anticipated discrimination including avoidance, issues in family, friendships and romantic relations, abuse and harassment, difficulties in the employment social environment, difficulties gaining employment, financial strain, difficulties gaining or maintaining housing, lower chances of education and negative impact on quality of life.

Twenty-seven studies described the impact of stigma on experienced and anticipated discrimination. Most of the studies were from: Eastern and Western Asia, Northern Europe, Eastern Europe and Northern America.

Four studies described the general impact of stigma on discrimination of people with mental illness, most often describing unfair treatment, being excluded or misjudged. Three studies described discrimination from law enforcement where participants felt being discredited or disbelieved when reporting crime to law enforcement officers,^264^ or being suspected or targetted for their mental illness.^222,265^ Three studies described participants disproportionately fearing or being preoccupied by the anticipation of discrimination.^203,266,267^ Finally, several studies described avoidance from family members, friends and neighbours as a form experienced discrimination. Avoidance was mostly defined as being excluded from social events, neighbors socially distancing themselves but also physically distancing with bans on sharing food and liquids.

Finally, one study reported participants experiencing anticipated discrimination as they believed  that a mental illness diagnosis would warrant unfair treatment in court such as unreasonable sentences.^202^

Issues in family, romantic and friendship relations as a consequence of stigma were described by forty-two studies. Most of the studies came from: Eastern Asia, South East Asia, Southern Asia, Western Europe, Eastern Europe, Northern Europe and Northern America.

Four studies described general negative impacts of stigma on family relations. Several studies described the negative impact of stigma on family relations. Three studies described participants being ostracized by family members due to beliefs of unreliability or incapability of people with mental illness, or feelings of shame on the side of family members.^200,202,268^

Six studies described participants feeling rejected or abandoned, and family members disengaging. Some studies also describe that participants have a feeling of detachment from the family due to their stigmatizing attitudes.^198,208,245,253^ Two studies described neglect from family members as a consequence of stigma, this was most often described by PWMI  living with their parents. Neglect was most often described as being denied food or separated physically from the rest of the family.^210,269^ Five studies described family members limiting the control PWMI  have regarding decisions around the household or even personal financial decisions.^238,268,270–272^ Finally, three studies described complete avoidance from family members such as not being invited to family functions or being told they are no longer part of the family.^211,249,269^

Fifteen studies described the impact of stigma on friendships. All of the studies describe similar consequences which are a loss of social contacts after disclosure of mental illness diagnosis, avoidance by friends, in some cases even being shunned by friends. Twenty-two studies described the consequences of stigma on romantic relationships. Six studies described the impact of stigma on marriage prospects. Most studies described that family members of either spouse were reluctant towards a marriage due to fear of heritability of mental illness, or belief of increased violence of people in mental illness. This often results in concealment of diagnosis in order to be able to marry.^209,252,261,273^ Some studies described divorce as a consequence of mental illness stigma - spouses decided to leave their partners once mental illness diagnosis was disclosed.^211,261,268,^ Not only marriage is impacted by mental illness stigma but also romantic relationships in general. Twelve studies described that romantic relationships are harder to gain due to mental illness stigma. According to participant reports, due to experienced stigma the dating pool was significantly reduced and due to anticipated stigma participants were often afraid to reach out and meet someone thus remaining single.^197,266,249^ Finally, some studies described that their relationships were unstable due to mental illness stigma, most often this was reported as lack of understanding or rejection from partners after disclosing diagnosis.^204,266,274,275^

Abuse and harassment were reported as a consequence of stigma in twenty-four studies. Most of the studies came from the following regions: South East Asia, Southern Asia, Southern Africa, Northern America and Northern Europe.

Abuse and harassment in general were described in eighteen studies, instances of abuse and harassment were most frequently defined as humiliation or ridicule both verbal and physical, three studies also report sexual abuse and violence.^249,261,272,276–278^ Abusers tended to be people from the community such as neighbors and family members. Physical violence such as beating or throwing things at PWMI was reported in ten studies.

Four studies described physical restriction such as chaining.^209,200,249,279^ Two studies described restriction as locking the person with mental illness in their room.^224,280^

Finally, one study described forced abortion as a result of stigmatizing beliefs regarding heredity of mental illness.^261,268^

Difficulties in the employment social environment were described in eighteen studies. Most of the studies were from: Northern Europe, Southern Europe, Northern America, Australia and New Zealand, South Eastern Asia, Southern Asia, Eastern Asia

Difficulties in the employment environment were most often linked to stigmatizing attitudes of either employers or co-workers. Employer stigma was most often described as impacting the amount of work or importance of work PWMI were assigned. Stigma also impacted whether participants received approval from employers for medical leave or time off to visit mental healthcare professionals. Co-worker stigma was described in seven studies, the main impacts of co-worker stigma was bullying in the workplace and social distancing leading to sharing less work responsibilities with the impacted person.^197,211,215,223,262,272,273,274,281^

Stigma consequences on gaining and retaining employment were described in twenty-two studies. Most of the studies were from: Eastern Asia, South Eastern Asia, Northern Europe, Western Europe and Northern Europe, Australia and New Zealand.

Most of the studies described participants not being offered the same work opportunities when disclosing mental health illness in their application, or even not obtaining the work position at all. Other studies also mentioned participants being fired once they disclosed their mental illness.^198,275,282–284^

Two studies described mental illness disrupting education, the studies were from: Eastern Africa, Southern Asia.

Both studies describe mental illness stigma disrupting the education progress of people with mental illness, this was either linked to a lack of accommodation on the side of educational institutions or withdrawal from educational activities due to anticipated discrimination.^273,285^

Finally, two studies described the impact of stigma on quality of life, the studies were from: Southern Africa, Northern Europe.

The two studies defined quality of life as a general living a full life carrying out normal activities like any other member in the participants community.^210,229^

## Economic consequences

The domain describes consequences or correlates pertaining to the following key themes: financial strain and difficulties in gaining and maintaining housing.

Financial strain as a consequence of mental illness stigma was reported by five studies. The studies came from: South East Asia, Australia and New Zealand, Southern Asia, Northern Europe.

All of the studies described financial strain being directly related to a lack of work opportunities due to stigma.^209,241,268,86^ One study described a discriminatory system of minimum wage set up.^223^

Five studies described difficulties in gaining housing due to mental illness stigma. The studies came from: Southern Asia, Western Europe and Northern America

Loganathan et al. describe a participant experience where parents refused to give them a share of the family property,^268^ similar experiences are described by Mathias et al.^270^ The three other studies describe landlords holding stigmatizing attitudes on the basis of which they refused to let participants live in their properties or evicted them.^204,280,286^

# Studies on intended behaviour

Studies on intended behaviour were not included in this review since it is not known to what extent intended behaviour correlates to true behaviour. However, if intended behaviour is a reflection of true behaviour, it can have serious negative impacts on the lives of PWMI. Studies on intended behaviour indicate that public stigma towards PWMI might be associated with decreased support for research on mental illness;^287^ less willingness to pay additional taxes to improve health and social services;^288^ increased support for mandated medication of PWMI,^289^ discriminatory insurance practices against PWMI;^290^  decreased willingness to provide medication counselling and decreased identification of drug-related problems;^291,292^  heightened perception of dangerousness and greater social distance of PWMI;^,293^lower perceived capacity of patients to report health problems and increased scepticism about recovery;^294,295^belief in patients’ need for long-term pharmacotherapies; belief that PWMI should be discriminated against when hospitalised; and less confidence in psychologists and family support.^293^ Within health professionals, stigma towards PWMI is linked to decreased intention to pursue psychiatry,^297^ less optimistic recovery attitudes and competencies,^298,299^increased endorsement of leveraged, directive or intrusive interventions,^300,301^ and lower willingness on ratings on care and support and higher ratings on fear, avoidance and negative stereotype expectations,^302-304^ negative attitudes towards people with severe mental illness as parents.^305^

# Appendix references

1      Montesinos AH, Rapp MA, Temur-Erman S, Heinz A, Hegerl U, Schouler-Ocak M. The influence of stigma on depression, overall psychological distress, and somatization among female Turkish migrants. Eur Psychiatry. 2012 Jun;27(S2):S22-6.

2      Thomé ES, Dargél AA, Migliavacca FM, Potter WA, Jappur DM, Kapczinski F, Ceresér KM. Stigma experiences in bipolar patients: the impact upon functioning.J Psychiatr Ment Health Nurs J. 2012 Oct;19(8):665-71.

3      Wood L, Byrne R, Burke E, Enache G, Morrison AP. The impact of stigma on emotional distress and recovery from psychosis: The mediatory role of internalised shame and self-esteem. Psychiatry Res. 2017 Sep 1;255:94-100.

4      Wood L, Irons C. Experienced stigma and its impacts in psychosis: The role of social rank and external shame. Psychol Psychother. 2017 Sep;90(3):419-31.

5      Phillips MR, Pearson V, Li F, Xu M, Yang L. Stigma and expressed emotion: a study of people with schizophrenia and their family members in China. BJPsych. 2002 Dec;181(6):488-93.

6      Alonso J, Saha S, Lim CC, Aguilar-Gaxiola S, Al-Hamzawi A, Benjet C, Bromet EJ, Degenhardt L, de Girolamo G, Esan O, Florescu S. The association between psychotic experiences and health-related quality of life: a cross-national analysis based on World Mental Health Surveys. Schizophr Res. 2018 Nov 1;201:46-53.

7      Zäske H, Linden M, Degner D, Jockers-Scherübl M, Klingberg S, Klosterkötter J, Maier W, Möller HJ, Sauer H, Schmitt A, Gaebel W. Stigma experiences and perceived stigma in patients with first-episode schizophrenia in the course of 1 year after their first in-patient treatment. Eur Arch Psychiatry Clin Neurosci. 2019 Jun;269(4):459-68.

8      Jormfeldt H, Arvidsson B, Svensson B, Hansson L. Construct validity of a health questionnaire intended to measure the subjective experience of health among patients in mental health services. J Psychiatr Ment Health Nurs. 2008 Apr;15(3):238-45.

9      Bahm A, Forchuk C. Interlocking oppressions: The effect of a comorbid physical disability on perceived stigma and discrimination among mental health consumers in Canada. Health Soc  care community. 2009 Jan;17(1):63-70.

10   Björkman T, Svensson B, Lundberg B. Experiences of stigma among people with severe mental illness. Reliability, acceptability and construct validity of the Swedish versions of two stigma scales measuring devaluation/discrimination and rejection experiences. Nord J Psychiatry. 2007 Jan 1;61(5):332-8.

11   Oleniuk A, Duncan CR, Tempier R. The impact of stigma of mental illness in a Canadian community: a survey of patients experiences. Community Ment Health J. 2013 Feb;49(1):127-32.

12   Pellet J, Golay P, Nguyen A, Suter C, Ismailaj A, Bonsack C, Favrod J. The relationship between self-stigma and depression among people with schizophrenia-spectrum disorders: A longitudinal study. Psychiatry Res. 2019 May 1;275:115-9.

13   Alonso J, Vilagut G, Adroher ND, Chatterji S, He Y, Andrade LH, Bromet E, Bruffaerts R, Fayyad J, Florescu S, De Girolamo G. Disability mediates the impact of common conditions on perceived health. PLoS One. 2013 Jun 6;8(6):e65858.

14   Grant JB, Bruce CP, Batterham PJ. Predictors of personal, perceived and self-stigma towards anxiety and depression. Epidemiol Psychiatr Sci. 2016 Jun;25(3):247-54.

15   Chung L, Pan AW, Hsiung PC. Quality of life for patients with major depression in Taiwan: A model-based study of predictive factors. Psychiatry Res. 2009 Jul 30;168(2):153-62.

16   Kao YC, Lien YJ, Chang HA, Wang SC, Tzeng NS, Loh CH. Evidence for the indirect effects of perceived public stigma on psychosocial outcomes: The mediating role of self-stigma. Psychiatry Res. 2016 Jun 30;240:187-95.

17   Ow CY, Lee BO. Relationships between perceived stigma, coping orientations, self-esteem, and quality of life in patients with schizophrenia. Asia Pacific Journal of Public Health. 2015 Mar;27(2):NP1932-41.

18   Pyne JM, Kuc EJ, Schroeder PJ, Fortney JC, Edlund M, Sullivan G. Relationship between perceived stigma and depression severity. J Nerv Ment. 2004 Apr 1;192(4):278-83.

19   Rayan A, Aldaieflih MT. Public stigma toward mental illness and its correlates among patients diagnosed with schizophrenia. Contemp. Nurse. 2019 Nov 2;55(6):522-32.

20   Shimotsu S, Horikawa N. Self-stigma in depressive patients: Association of cognitive schemata, depression, and self-esteem. Asian J Psychiatr. 2016 Dec 1;24:125-9.

21   Vauth R, Kleim B, Wirtz M, Corrigan PW. Self-efficacy and empowerment as outcomes of self-stigmatizing and coping in schizophrenia. Psychiatry Res. 2007 Feb 28;150(1):71-80.

22   MacDougall AG, Vandermeer MR, Norman RM. Negative future self as a mediator in the relationship between insight and depression in psychotic disorders. Schizophr Res. 2015 Jun 1;165(1):66-9.

23   Mueser KT, DeTore NR, Kredlow MA, Bourgeois ML, Penn DL, Hintz K. Clinical and demographic correlates of stigma in first‐episode psychosis: the impact of duration of untreated psychosis. Acta Psychiatr Scand. 2020 Feb;141(2):157-66.

24   Denenny D, Thompson E, Pitts SC, Dixon LB, Schiffman J. Subthreshold psychotic symptom distress, self-stigma, and peer social support among college students with mental health concerns. Psychiatr Rehabil J. 2015 Jun;38(2):164.

25   Griffiths S, Mond JM, Murray SB, Touyz S. The prevalence and adverse associations of stigmatization in people with eating disorders. Int J Eat Disord. 2015 Sep;48(6):767-74.

26   Oexle N, Ajdacic-Gross V, Kilian R, Müller M, Rodgers S, Xu Z, Rössler W, Rüsch N. Mental illness stigma, secrecy and suicidal ideation. Epidemiol Psychiatr Sci. 2017 Feb;26(1):53-60.

27   Xu Z, Mayer B, Müller M, Heekeren K, Theodoridou A, Dvorsky D, Metzler S, Oexle N, Walitza S, Rössler W, Rüsch N. Stigma and suicidal ideation among young people at risk of psychosis after one year. Psychiatry Res. 2016 Sep 30;243:219-24.

28   Pyle M, Morrison AP. Internalised stereotypes across ultra-high risk of psychosis and psychosis populations. Psychosis. 2017 Apr 3;9(2):110-8.

29   Aydemir O, Akkaya C. Association of social anxiety with stigmatisation and low self-esteem in remitted bipolar patients. Acta Neuropsychiatr. 2011 Oct;23(5):224-8.

30   Vázquez GH, Kapczinski F, Magalhaes PV, Córdoba R, Jaramillo CL, Rosa AR, De Carmona MS, Tohen M, on Bipolar TI. Stigma and functioning in patients with bipolar disorder. J Affect Disord. 2011 Apr 1;130(1-2):323-7.

31   Lundberg B, Hansson L, Wentz E, Björkman T. Sociodemographic and clinical factors related to devaluation/discrimination and rejection experiences among users of mental health services. Social Psychiatry Psychiatr Epidemiol. 2007 Apr;42(4):295-300.

32   Belvederi Murri M, Amore M, Calcagno P, Respino M, Marozzi V, Masotti M, Bugliani M, Innamorati M, Pompili M, Galderisi S, Maj M. The “insight paradox” in schizophrenia: magnitude, moderators and mediators of the association between insight and depression. Schizophr Bull. 2016 Sep 1;42(5):1225-33.

33   Staring AB, Van der Gaag M, Van den Berge M, Duivenvoorden HJ, Mulder CL. Stigma moderates the associations of insight with depressed mood, low self-esteem, and low quality of life in patients with schizophrenia spectrum disorders. Schizophr Res. 2009 Dec 1;115(2-3):363-9.

34   Grover S, Avasthi A, Singh A, Dan A, Neogi R, Kaur D, Lakdawala B, Rozatkar AR, Nebhinani N, Patra S, Sivashankar P. Stigma experienced by patients with severe mental disorders: A nationwide multicentric study from India. Psychiatry Res. 2017 Nov 1;257:550-8.

35   Yanos PT, Roe D, Markus K, Lysaker PH. Pathways between internalized stigma and outcomes related to recovery in schizophrenia spectrum disorders. Psychiatr Serv. 2008 Dec;59(12):1437-42.

36   Lanfredi M, Zoppei S, Ferrari C, Bonetto C, Van Bortel T, Thornicroft G, Knifton L, Quinn N, Rossi G, Lasalvia A. Self-stigma as a mediator between social capital and empowerment among people with major depressive disorder in Europe: The ASPEN study. Eur Psychiatry. 2015 Jan;30(1):58-64.

37   Kira IA, Lewandowski L, Ashby JS, Templin T, Ramaswamy V, Mohanesh J. The traumatogenic dynamics of internalized stigma of mental illness among Arab American, Muslim, and refugee clients.J Am Psychiatr Nurses Assoc. 2014 Jul;20(4):250-66.

38   Lee AM, Simeon D, Cohen LJ, Samuel J, Steele A, Galynker II. Predictors of patient and caregiver distress in an adult sample with bipolar disorder seeking family treatment. J Nerv Ment. 2011 Jan 1;199(1):18-24.

39   Lorona RT, Fergus TA, Valentiner DP, Miller LM, McGrath PB. Self-Stigma and Etiological Attributions About Symptoms Among Individuals Diagnosed With an Anxiety Disorder: Relations With Symptom Severity and Symptom Improvement Following CBT.J Soc Clin Psychol. 2018 Sep;37(7):536-57.

40   Ociskova M, Prasko J, Kamaradova D. Relationship between personality and self-stigma in mixed neurotic spectrum and depressive disorders–cross sectional study. Act Nerv Super Rediviva. 2015 Mar 28;57(1-2):22-9.

41   Pyle M, Brabban A, Drage L, Spencer H, Turkington D, Morrison A. Associations between internalised stereotypes of psychosis and emotional dysfunction in people with psychosis not taking antipsychotic medication. Psychosis. 2015 Jul 3;7(3):217-27.

42   Sickel AE, Seacat JD, Nabors NA. Mental health stigma: Impact on mental health treatment attitudes and physical health. J Health Psychol. 2019 Apr;24(5):586-99.

43   Kira IA, Ramaswamy V, Lewandowski L, Mohanesh J, Abdul-Khalek H. Psychometric assessment of the Arabic version of the Internalized Stigma of Mental Illness (ISMI) measure in a refugee population. Transcult Psychiatry. 2015 Oct;52(5):636-58.

44   Assefa D, Shibre T, Asher L, Fekadu A. Internalized stigma among patients with schizophrenia in Ethiopia: a cross-sectional facility-based study. BMC psychiatry. 2012 Dec;12(1):1-0.

45   Byrow Y, Pajak R, McMahon T, Rajouria A, Nickerson A. Barriers to mental health help-seeking amongst refugee men.Int. J. Environ. Res. Public Health. 2019 Aug;16(15):2634.

46   James TT, Kutty VR. Assessment of internalized stigma among patients with mental disorders in Thiruvananthapuram district, Kerala, India.Asia Pacific Journal of Public Health. 2015 May;27(4):439-49.

47   Grover S, Hazari N, Aneja J, Chakrabarti S, Sharma S, Avasthi A. Recovery and its correlates among patients with bipolar disorder: A study from a tertiary care centre in North India.Int. J. Soc. Psychiatry. 2016 Dec;62(8):726-36.

48   Grover S, Sahoo S, Chakrabarti S, Avasthi A. Association of internalized stigma and insight in patients with schizophrenia.Int J Cult Ment Health. 2018 Jul 3;11(3):338-50.

49   Masuch TV, Bea M, Alm B, Deibler P, Sobanski E. Internalized stigma, anticipated discrimination and perceived public stigma in adults with ADHD.Atten Defic Hyperact Disord. 2019 Jun;11(2):211-20.

50   Kim WJ, Song YJ, Ryu HS, Ryu V, Kim JM, Ha RY, Lee SJ, Namkoong K, Ha K, Cho HS. Internalized stigma and its psychosocial correlates in Korean patients with serious mental illness. Psychiatry Res. 2015 Feb 28;225(3):433-9.

51   Lv Y, Wolf A, Wang X. Experienced stigma and self-stigma in Chinese patients with schizophrenia. Gen Hosp Psychiatry. 2013 Jan 1;35(1):83-8.

52   Pyle M, Stewart SL, French P, Byrne R, Patterson P, Gumley A, Birchwood M, Morrison AP. Internalized stigma, emotional dysfunction and unusual experiences in young people at risk of psychosis. Early Interv Psychiatry. 2015 Apr;9(2):133-40.

53   Touriño R, Acosta FJ, Giráldez A, Álvarez J, González JM, Abelleira C, Benítez N, Baena E, Fernández JA, Rodriguez CJ. Suicidal risk, hopelessness and depression in patients with schizophrenia and internalized stigma.Actas Esp. Psiquiatr. 2018 Mar.

54   Vrbova K, Prasko J, Ociskova M, Holubova M, Kantor K, Kolek A, Grambal A, Slepecky M. Suicidality, self-stigma, social anxiety and personality traits in stabilized schizophrenia patients–a cross-sectional study.Neuropsychiatr Dis Treat. 2018;14:1415.

55   Cai C, Yu L. Quality of life in patients with schizophrenia in China: relationships among demographic characteristics, psychosocial variables, and symptom severity.J Psychosoc Nurs Ment Health Serv. 2017 Aug 1;55(8):48-54.

56   Chan KK, Lam CB. The impact of familial expressed emotion on clinical and personal recovery among patients with psychiatric disorders: The mediating roles of self-stigma content and process.Am J Orthopsychiatry. 2018;88(6):626.

57   Drapalski AL, Lucksted A, Perrin PB, Aakre JM, Brown CH, DeForge BR, Boyd JE. A model of internalized stigma and its effects on people with mental illness. Psychiatr Serv. 2013 Mar;64(3):264-9.

58   Howland M, Levin J, Blixen C, Tatsuoka C, Sajatovic M. Mixed-methods analysis of internalized stigma correlates in poorly adherent individuals with bipolar disorder. Compr Psychiatry. 2016 Oct 1;70:174-80.

59   Karidi MV, Stefanis CN, Theleritis C, Tzedaki M, Rabavilas AD, Stefanis NC. Perceived social stigma, self-concept, and self-stigmatization of patient with schizophrenia. Compr Psychiatry. 2010 Jan 1;51(1):19-30.

60   Kvrgic S, Cavelti M, Beck EM, Rüsch N, Vauth R. Therapeutic alliance in schizophrenia: the role of recovery orientation, self-stigma, and insight. Psychiatry Res. 2013 Aug 30;209(1):15-20.

61   Mosanya TJ, Adelufosi AO, Adebowale OT, Ogunwale A, Adebayo OK. Self-stigma, quality of life and schizophrenia: An outpatient clinic survey in Nigeria. Int J Soc Psychiatry. 2014 Jun;60(4):377-86.

62   Firmin RL, Lysaker PH, Luther L, Yanos PT, Leonhardt B, Breier A, Vohs JL. Internalized stigma in adults with early phase versus prolonged psychosis. Early Interv Psychiatry. 2019 Aug;13(4):745-51.

63   Markowitz FE, Angell B, Greenberg JS. Stigma, reflected appraisals, and recovery outcomes in mental illness. Soc Psychol Q. 2011 Jun;74(2):144-65.

64   Nabors LM, Yanos PT, Roe D, Hasson-Ohayon I, Leonhardt BL, Buck KD, Lysaker PH. Stereotype endorsement, metacognitive capacity, and self-esteem as predictors of stigma resistance in persons with schizophrenia. Compr Psychiatry. 2014 May 1;55(4):792-8.

65   Chan KK, Mak WW. The mediating role of self-stigma and unmet needs on the recovery of people with schizophrenia living in the community. Qual Life Res. 2014 Nov;23(9):2559-68.

66   Chan RC, Mak WW. Common sense model of mental illness: understanding the impact of cognitive and emotional representations of mental illness on recovery through the mediation of self-stigma. Psychiatry Res. 2016 Dec 30;246:16-24.

67   Cinculova A, Prasko J, Kamaradova D, Ociskova M, Latalova K, Vrbova K, Kubinek R, Mainerova B, Grambal A, Tichackova A. Adherence, self-stigma and discontinuation of pharmacotherapy in patients with anxiety disorders–cross-sectional study. Neuroendocrinol Lett. 2017 Jan 1;38(6):429-36.

68   Holubova M, Prasko J, Ociskova M, Kantor K, Vanek J, Slepecky M, Vrbova K. Quality of life, self-stigma, and coping strategies in patients with neurotic spectrum disorders: a cross-sectional study. Psychol Res Behav. 2019;12:81.

69   Holubova M, Prasko J, Hruby R, Latalova K, Kamaradova D, Marackova M, Slepecky M, Gubova T. Coping strategies and self-stigma in patients with schizophrenia-spectrum disorders. Patient Prefer Adher. 2016;10:1151.

70   Holubova M, Prasko J, Ociskova M, Marackova M, Grambal A, Slepecky M. Self-stigma and quality of life in patients with depressive disorder: a cross-sectional study. Neuropsychiatr Dis Treat. 2016;12:2677.

71   Kamaradova D, Latalova K, Prasko J, Kubinek R, Vrbova K, Mainerova B, Cinculova A, Ociskova M, Holubova M, Smoldasova J, Tichackova A. Connection between self-stigma, adherence to treatment, and discontinuation of medication. Patient Preference Adherence. 2016;10:1289.

72   Holubova M, Prasko J, Latalova K, Ociskova M, Grambal A, Kamaradova D, Vrbova K, Hruby R. Are self-stigma, quality of life, and clinical data interrelated in schizophrenia spectrum patients? A cross-sectional outpatient study. Patient Prefer Adher. 2016;10:265.

73   Vrbova K, Prasko J, Holubova M, Kamaradova D, Ociskova M, Marackova M, Latalova K, Grambal A, Slepecky M, Zatkova M. Self-stigma and schizophrenia: a cross-sectional study. Neuropsychiatr Dis Treat. 2016;12:3011.

74   Lysaker PH, Tunze C, Yanos PT, Roe D, Ringer J, Rand K. Relationships between stereotyped beliefs about mental illness, discrimination experiences, and distressed mood over 1 year among persons with schizophrenia enrolled in rehabilitation. Soc Psychiatry Psychiatr Epidemiol 2012 Jun;47(6):849-55.

75   Pearl RL, Forgeard MJ, Rifkin L, Beard C, Björgvinsson T. Internalized stigma of mental illness: Changes and associations with treatment outcomes. Stigma Health. 2017 Feb;2(1):2.

76   Lien YJ, Chang HA, Kao YC, Tzeng NS, Lu CW, Loh CH. Insight, self-stigma and psychosocial outcomes in Schizophrenia: a structural equation modelling approach. Epidemiol Psychiatr Sci. 2018 Apr;27(2):176-85.

77   Rossi A, Galderisi S, Rocca P, Bertolino A, Rucci P, Gibertoni D, Stratta P, Bucci P, Mucci A, Aguglia E, Amodeo G. Personal resources and depression in schizophrenia: the role of self-esteem, resilience and internalized stigma. Psychiatry Res. 2017 Oct 1;256:359-64.

78   Schrank B, Amering M, Hay AG, Weber M, Sibitz I. Insight, positive and negative symptoms, hope, depression and self-stigma: a comprehensive model of mutual influences in schizophrenia spectrum disorders. Epidemiol Psychiatr Sci. 2014 Sep;23(3):271-9.

79   Griffiths S, Mitchison D, Murray SB, Mond JM, Bastian BB. How might eating disorders stigmatization worsen eating disorders symptom severity? Evaluation of a stigma internalization model. Int J Eat Disord. 2018 Aug;51(8):1010-4.

80   Zhang TM, Wong IY, Yu YH, Ni SG, He XS, Bacon-Shone J, Gong K, Huang CH, Hu Y, Tang MM, Cao W. An integrative model of internalized stigma and recovery-related outcomes among people diagnosed with schizophrenia in rural China. Social Psychiatry Psychiatr Epidemiol. 2019 Aug;54(8):911-8.

81   Marcussen K, Gallagher M, Ritter C. Mental illness as a stigmatized identity. Society Mental Health. 2019 Jul;9(2):211-27.

82   Farrelly S, Jeffery D, Ruesch N, Williams P, Thornicroft G, Clement S. The link between mental health-related discrimination and suicidality: service user perspectives. Psychol Med. 2015 Jul;45(10):2013-22.

83   Boyd JE, Bassett ED, Hoff R. Internalized stigma of mental illness and depressive and psychotic symptoms in homeless veterans over 6 months. Psychiatry Res. 2016 Jun 30;240:253-9.

84   Ritsher JB, Phelan JC. Internalized stigma predicts erosion of morale among psychiatric outpatients. Psychiatry Res. 2004 Dec 30;129(3):257-65.

85   Xu Z, Lay B, Oexle N, Drack T, Bleiker M, Lengler S, Blank C, Müller M, Mayer B, Rössler W, Rüsch N. Involuntary psychiatric hospitalisation, stigma stress and recovery: a 2-year study. Epidemiol Psychiatr Sci. 2019 Aug;28(4):458-65.

86   Pyle M, Morrison AP. “It’s just a very taboo and secretive kind of thing”: making sense of living with stigma and discrimination from accounts of people with psychosis. Psychosis. 2014 Jul 3;6(3):195-205.

87   Ben-Zeev D, Frounfelker R, Morris SB, Corrigan PW. Predictors of self-stigma in schizophrenia: new insights using mobile technologies. J Dual Diag. 2012 Nov 1;8(4):305-14.

88   Oexle N, Rüsch N, Viering S, Wyss C, Seifritz E, Xu Z, Kawohl W. Self-stigma and suicidality: a longitudinal study.Eur Arch Psychiatry Clin Neurosci. 2017 Jun;267(4):359-61.

89   Markowitz FE. The effects of stigma on the psychological well-being and life satisfaction of persons with mental illness.J Health Soc Behav. 1998 Dec 1:335-47.

90   Lysaker P, Yanos P, Outcalt J, Roe D. Association of stigma, self-esteem, and symptoms with concurrent and prospective assessment of social anxiety in schizophrenia.Clin Schizophr Relat Psychoses. 2010 Apr 1;4(1):41-8.

91   Cavelti M, Rüsch N, Vauth R. Is living with psychosis demoralizing?: Insight, self-stigma, and clinical outcome among people with schizophrenia across 1 year. J Nerv Ment. 2014 Jul 1;202(7):521-9.

92   Lysaker PH, Davis LW, Warman DM, Strasburger A, Beattie N. Stigma, social function and symptoms in schizophrenia and schizoaffective disorder: Associations across 6 months. Psychiatry Res. 2007 Jan 15;149(1-3):89-95.

93   Rüsch N, Corrigan PW, Wassel A, Michaels P, Larson JE, Olschewski M, Wilkniss S, Batia K. Self-stigma, group identification, perceived legitimacy of discrimination and mental health service use. Br J Psych. 2009 Dec;195(6):551-2.

94   Nilsson KK, Kugathasan P, Straarup KN. Characteristics, correlates and outcomes of perceived stigmatization in bipolar disorder patients. J Affect Disord. 2016 Apr 1;194:196-201.

95   Ilic M, Reinecke J, Bohner G, Röttgers HO, Beblo T, Driessen M, Frommberger U, Corrigan PW. Belittled, avoided, ignored, denied: Assessing forms and consequences of stigma experiences of people with mental illness. Basic Appl Soc Psych. 2013 Jan 1;35(1):31-40.

96   Rüsch N, Heekeren K, Theodoridou A, Müller M, Corrigan PW, Mayer B, Metzler S, Dvorsky D, Walitza S, Rössler W. Stigma as a stressor and transition to schizophrenia after one year among young people at risk of psychosis. Schizophr Res. 2015 Aug 1;166(1-3):43-8.

97   Hoge CW, Grossman SH, Auchterlonie JL, Riviere LA, Milliken CS, Wilk JE. PTSD treatment for soldiers after combat deployment: Low utilization of mental health care and reasons for dropout. Psychiatr Serv. 2014 Aug;65(8):997-1004.

98   Adeponle AB, Baduku AS, Adelekan ML, Suleiman GT, Adeyemi SO. Prospective study of psychiatric follow-up default and medication compliance after discharge at a psychiatric hospital in Nigeria. Community Ment Health J. 2009 Feb;45(1):19-25.

99   Conner KO, Copeland VC, Grote NK, Koeske G, Rosen D, Reynolds III CF, Brown C. Mental health treatment seeking among older adults with depression: the impact of stigma and race. Am J Geriatr Psych. 2010 Jun 1;18(6):531-43

100     Kondrátová L, König D, Mladá K, Winkler P. Correlates of negative attitudes towards   medication in people with schizophrenia. Psychiatr Quart. 2019 Mar;90(1):159-69.

101     Sedlácková Z, Kamarádová D, Prasko J, Látalová K, Ocisková M, Ocisková M, Vrbová K. Treatment adherence and self-stigma in patients with depressive disorder in remission–a cross-sectional study. Neuro Endocrinol Lett. 2015 Jan 1;36(2):171-7.

102     Vrbová K, Kamarádová D, Látalová K, Ocisková M, Praško J, Mainerová B, Cinculová A, Kubínek R, Ticháčková A. Self-stigma and adherence to medication in patients with psychotic disorders–cross-sectional study. Neuro Endocrinol Lett. 2014 Jan 1;35(7):645-52.

103     Yılmaz E, Okanlı A. The effect of internalized stigma on the adherence to treatment in patients with schizophrenia. Arch Psychiatr Nurs. 2015 Oct 1;29(5):297-301.

104     Tsang HW, Fung KM, Corrigan PW. Psychosocial and socio-demographic correlates of medication compliance among people with schizophrenia.J Behav Ther Exp Psychiatry. 2009 Mar 1;40(1):3-14.

105     Chen FP, Wu HC, Huang CJ. Influences of attribution and stigma on working relationships with providers practicing western psychiatry in the Taiwanese context. Psychiatr Quart. 2014 Dec;85(4):439-51.

106     Lim L, Goh J, Chan YH. Internalized stigma, disclosure and self-esteem among psychiatric patients in a general hospital outpatient clinic. Australas Psychiatry. 2019 Dec;27(6):584-8

107      Brohan E, Elgie R, Sartorius N, Thornicroft G, GAMIAN-Europe Study Group. Self-stigma, empowerment and perceived discrimination among people with schizophrenia in 14 European countries: The GAMIAN-Europe study. Schizophr Res. 2010 Sep 1;122(1-3):232-8.

108     Brohan E, Gauci D, Sartorius N, Thornicroft G, GAMIAN–Europe Study Group. Self-stigma, empowerment and perceived discrimination among people with bipolar disorder or depression in 13 European countries: The GAMIAN–Europe study. J Affect Disord. 2011 Mar 1;129(1-3):56-63.

109     Li J, Guo YB, Huang YG, Liu JW, Chen W, Zhang XY, Evans-Lacko S, Thornicroft G. Stigma and discrimination experienced by people with schizophrenia living in the community in Guangzhou, China. Psychiatr Res. 2017 Sep 1;255:225-31.

110     Chronister J, Chou CC, Liao HY. The role of stigma coping and social support in mediating the effect of societal stigma on internalized stigma, mental health recovery, and quality of life among people with serious mental illness. J Community Psychol. 2013 Jul;41(5):582-600.

111     Arnaez JM, Krendl AC, McCormick BP, Chen Z, Chomistek AK. The association of depression stigma with barriers to seeking mental health care: A cross-sectional analysis. J Ment Health. 2020 Mar 3;29(2):182-90.

112     Hackler AH. Seeking professional help for an eating disorder: The role of stigma, anticipated outcomes, and attitudes. Iowa State University; 2007.

113     Rusch LC, Kanter JW, Manos RC, Weeks CE. Depression stigma in a predominantly low income African American sample with elevated depressive symptoms. J Nerv Ment. 2008 Dec 1;196(12):919-22.

114     Raguram R, Raghu TM, Vounatsou P, Weiss MG. Schizophrenia and the cultural epidemiology of stigma in Bangalore, India. J Nerv Ment. 2004 Nov 1;192(11):734-44.

115     Espinosa R, Valiente C, Rigabert A, Song H. Recovery style and stigma in psychosis: the healing power of integrating. Cogn Neuropsychiatry. 2016 Mar 3;21(2):146-55.

116     Vass V, Morrison AP, Law H, Dudley J, Taylor P, Bennett KM, Bentall RP. How stigma impacts on people with psychosis: The mediating effect of self-esteem and hopelessness on subjective recovery and psychotic experiences. Psychiatry Res. 2015 Dec 15;230(2):487-95.

117     Chen H, Coakley EH, Cheal K, Maxwell J, Costantino G, Krahn DD, Malgady RG, Durai UN, Quijano LM, Zaman S, Miller CJ. Satisfaction with mental health services in older primary care patients.Am J Geriatr Psychiatry. 2006 Apr 1;14(4):371-9.

118     Feldhaus T, Falke S, von Gruchalla L, Maisch B, Uhlmann C, Bock E, Lencer R. The impact of self-stigmatization on medication attitude in schizophrenia patients. Psychiatry Res. 2018 Mar 1;261:391-9.

119     Norman RM, Windell D, Lynch J, Manchanda R. Parsing the relationship of stigma and insight to psychological well-being in psychotic disorders. Schizophr Res. 2011 Dec 1;133(1-3):3-7.

120     Pérez-Garín D, Molero F, Bos AE. Internalized mental illness stigma and subjective well-being: The mediating role of psychological well-being. Psychiatry Res. 2015 Aug 30;228(3):325-31.

121     Pérez-Garín D, Molero F, Bos AE. The effect of personal and group discrimination on the subjective well-being of people with mental illness: The role of internalized stigma and collective action intention. Psychol Health Med. 2017 Apr 21;22(4):406-14.

122     Fung KM, Tsang HW, Corrigan PW, Lam CS, Cheng WM. Measuring self-stigma of mental illness in China and its implications for recovery. Int J Soc Psychiatry. 2007 Sep;53(5):408-18.

123     Howland M, Levin J, Blixen C, Tatsuoka C, Sajatovic M. Mixed-methods analysis of internalized stigma correlates in poorly adherent individuals with bipolar disorder. Comp Psychiatry. 2016 Oct 1;70:174-80.

124     Kleim B, Vauth R, Adam G, Stieglitz RD, Hayward P, Corrigan P. Perceived stigma predicts low self-efficacy and poor coping in schizophrenia. J Ment Health. 2008 Jan 1;17(5):482-91.

125     Sibitz I, Amering M, Unger A, Seyringer ME, Bachmann A, Schrank B, Benesch T, Schulze B, Woppmann A. The impact of the social network, stigma and empowerment on the quality of life in patients with schizophrenia. Eur Psychiatry. 2011 Jan;26(1):28-33.

126     Fung KM, Tsang HW, Chan F. Self-stigma, stages of change and psychosocial treatment adherence among Chinese people with schizophrenia: a path analysis. Soc Psychiatry Psychiatr Epidemiol. 2010 May;45(5):561-8.

127     Jahn DR, Leith J, Muralidharan A, Brown CH, Drapalski AL, Hack S, Lucksted A. The influence of experiences of stigma on recovery: Mediating roles of internalized stigma, self-esteem, and self-efficacy. Psychiatr Rehabil J. 2020 Jun;43(2):97.

128     Mashiach-Eizenberg M, Hasson-Ohayon I, Yanos PT, Lysaker PH, Roe D. Internalized stigma and quality of life among persons with severe mental illness: The mediating roles of self-esteem and hope. Psychiatry Res. 2013 Jun 30;208(1):15-20.

129     Morgades-Bamba CI, Fuster-Ruizdeapodaca MJ, Molero F. The impact of internalized stigma on the well-being of people with Schizophrenia. Psychiatry Res. 2019 Jan 1;271:621-7.

130     Lien YJ, Chang HA, Kao YC, Tzeng NS, Lu CW, Loh CH. The impact of cognitive insight, self-stigma, and medication compliance on the quality of life in patients with schizophrenia.Eur Arch Psychiatry Clin Neurosci. 2018 Feb;268(1):27-38.

131     Oliveira SE, Carvalho H, Esteves F. Internalized stigma and quality of life domains among people with mental illness: the mediating role of self-esteem. J Ment Health. 2016 Jan 2;25(1):55-61.

132     Vass V, Sitko K, West S, Bentall RP. How stigma gets under the skin: the role of stigma, self-stigma and self-esteem in subjective recovery from psychosis. Psychosis. 2017 Jul 3;9(3):235-44.

133     Chan KK, Fung WT. The impact of experienced discrimination and self-stigma on sleep and health-related quality of life among individuals with mental disorders in Hong Kong. Qual Life Res. 2019 Aug;28(8):2171-82.

134     Sirey JA, Bruce ML, Alexopoulos GS, Perlick DA, Friedman SJ, Meyers BS. Stigma as a barrier to recovery: Perceived stigma and patient-rated severity of illness as predictors of antidepressant drug adherence. Psychiatr Serv. 2001 Dec;52(12):1615-20.

135     Sirey JA, Bruce ML, Alexopoulos GS, Perlick DA, Raue P, Friedman SJ, Meyers BS. Perceived stigma as a predictor of treatment discontinuation in young and older outpatients with depression.Am. J. Psychiatry. 2001 Mar 1;158(3):479-81.

136     Sher I, McGinn L, Sirey JA, Meyers B. Effects of caregivers' perceived stigma and causal beliefs on patients' adherence to antidepressant treatment. Psychiatr Serv. 2005 May;56(5):564-9.

137     Chen H, Fang X, Liu C, Hu W, Lan J, Deng L. Associations among the number of mental health problems, stigma, and seeking help from psychological services: A path analysis model among Chinese adolescents.Child. Youth Serv. Rev. 2014 Sep 1;44:356-62.

138     Chien WT, Yeung FK, Chan AH. Perceived stigma of patients with severe mental illness in Hong Kong: Relationships with patients’ psychosocial conditions and attitudes of family caregivers and health professionals.Adm Policy Ment Health. 2014 Mar;41(2):237-51.

139     Komiti A, Judd F, Jackson H. The influence of stigma and attitudes on seeking help from a GP for mental health problems. Soc Psychiatry Psychiatr Epidemiol. 2006 Sep;41(9):738-45.

140     Esquinas-Requena JL, Lozoya-Moreno S, García-Nogueras I, Atienzar-Núñez P, Sánchez-Jurado PM, Abizanda P. La anemia aumenta el riesgo de mortalidad debido a fragilidad y discapacidad en mayores: Estudio FRADEA. Aten Primaria. 2020 Aug 1;52(7):452-61.

141     Sharp ML, Fear NT, Rona RJ, Wessely S, Greenberg N, Jones N, Goodwin L. Stigma as a barrier to seeking health care among military personnel with mental health problems. Epidemiol. 2015 Jan 1;37(1):144-62.

142     Tsai J, Whealin JM, Pietrzak RH. Asian American and Pacific Islander military veterans in the United States: Health service use and perceived barriers to mental health services. Am J Public Health. 2014 Sep;104(S4):S538-47.

143     Muñoz M, Sanz M, Pérez-Santos E, de los Ángeles Quiroga M. Proposal of a socio–cognitive–behavioral structural equation model of internalized stigma in people with severe and persistent mental illness. Psychiatr Res. 2011 Apr 30;186(2-3):402-8.

144     Brown C, Conner KO, Copeland VC, Grote N, Beach S, Battista D, Reynolds III CF. Depression stigma, race, and treatment seeking behavior and attitudes. J. Community Psychol. 2010 Apr;38(3):350-68.

145     Corrigan PW, Bink AB, Schmidt A, Jones N, Rüsch N. What is the impact of self-stigma? Loss of self-respect and the “why try” effect. J Ment Health. 2016 Jan 2;25(1):10-5.

146     Elliott M, Doane MJ. Stigma management of mental illness: Effects of concealment, discrimination, and identification on well-being. Self Identity. 2015 Nov 2;14(6):654-74.

147     Magallares A, Perez-Garin D, Molero F. Social Stigma and well-being in a sample of schizophrenia patients.Clin Schizophr Relat Psychoses. 2016;10(1):51-7.

148     MacInnes DL, Lewis M. The evaluation of a short group programme to reduce self‐stigma in people with serious and enduring mental health problems.J Psychiatr Ment Health Nurs. 2008 Jan;15(1):59-65.

149     Cavelti M, Kvrgic S, Beck EM, Rüsch N, Vauth R. Self-stigma and its relationship with insight, demoralization, and clinical outcome among people with schizophrenia spectrum disorders. Compr Psychiatry. 2012 Jul 1;53(5):468-79.

150     Pardo Cely EE, Fierro M, Ibánez Pinilla M. Prevalence and associated factors of non-adherence to treatment in bipolar disorder. Rev Colomb Psiquiatr. 2011 Jan;40(1):85-98.

151     Fernando SM, Deane FP, McLeod HJ. The delaying effect of stigma on mental health help‐seeking in Sri Lanka. Asia Pac Psychiatry. 2017;9(1).

152     Depla MF, de Graaf R, van Weeghel J, Heeren TJ. The role of stigma in the quality of life of older adults with severe mental illness.Int. J. Geriatr. Psychiatry. 2005 Feb;20(2):146-53.

153     Fowler CA, Rempfer MV, Murphy ME, Barnes AL, Hoover ED. Exploring the paradoxical effects of insight and stigma in psychological recovery.N. Am. J. Psychol. 2015 Mar 1;17(1):151-74.

154     Bos AE, Kanner D, Muris P, Janssen B, Mayer B. Mental illness stigma and disclosure: Consequences of coming out of the closet.Issues Ment. Health Nurs. 2009 Jan 1;30(8):509-13.

155     King M, Dinos S, Shaw J, Watson R, Stevens S, Passetti F, Weich S, Serfaty M. The Stigma Scale: development of a standardised measure of the stigma of mental illness. The BJ Psych. 2007 Mar;190(3):248-54.

156     Lundberg B, Hansson L, Wentz E, Björkman T. Are stigma experiences among persons with mental illness, related to perceptions of self‐esteem, empowerment and sense of coherence?.J Psychiatr Ment Health Nurs. 2009 Aug;16(6):516-22.

157     Hansson L, Björkman T. Empowerment in people with a mental illness: reliability and validity of the Swedish version of an empowerment scale.Scand J Caring Sci. 2005 Mar;19(1):32-8.

158     Xu Z, Müller M, Heekeren K, Theodoridou A, Dvorsky D, Metzler S, Brabban A, Corrigan PW, Walitza S, Rössler W, Rüsch N. Self-labelling and stigma as predictors of attitudes towards help-seeking among people at risk of psychosis: 1-year follow-up. Eur Arch Psychiatry Clin Neurosci. Neuroscience. 2016 Feb;266(1):79-82.

159     Schomerus G, Stolzenburg S, Freitag S, Speerforck S, Janowitz D, Evans-Lacko S, Muehlan H, Schmidt S. Stigma as a barrier to recognizing personal mental illness and seeking help: a prospective study among untreated persons with mental illness. Eur Arch Psychiatry Clin Neurosci. 2019 Jun;269(4):469-79.

160     Yen CF, Chen CC, Lee Y, Tang TC, Ko CH, Yen JY. Association between quality of life and self‐stigma, insight, and adverse effects of medication in patients with depressive disorders. Depress Anxiety. 2009 Nov;26(11):1033-9.

161     Oexle N, Müller M, Kawohl W, Xu Z, Viering S, Wyss C, Vetter S, Rüsch N. Self-stigma as a barrier to recovery: a longitudinal study. Eur Arch Psychiatry Clin Neurosci. 2018 Mar;268(2):209-12.

162     Rüsch N, Müller M, Heekeren K, Theodoridou A, Metzler S, Dvorsky D, Corrigan PW, Walitza S, Rössler W. Longitudinal course of self-labeling, stigma stress and well-being among young people at risk of psychosis. Schizophr Res 2014 Sep 1;158(1-3):82-4.

163     Link BG, Struening EL, Neese-Todd S, Asmussen S, Phelan JC. Stigma as a barrier to recovery: The consequences of stigma for the self-esteem of people with mental illnesses. Psychiatr Serv. 2001 Dec;52(12):1621-6.

164     Xu Z, Müller M, Lay B, Oexle N, Drack T, Bleiker M, Lengler S, Blank C, Vetter S, Rössler W, Rüsch N. Involuntary hospitalization, stigma stress and suicidality: a longitudinal study. Soc Psychiatry Psychiatr Epidemiol. 2018 Mar;53(3):309-12.

165     Livingston J. Self‐stigma and quality of life among people with mental illness who receive compulsory community treatment services. J Comm Psychol. 2012 Aug;40(6):699-714.

166     Rüsch N, Malzer A, Oexle N, Waldmann T, Staiger T, Bahemann A, Wigand ME, Becker T, Corrigan PW. Disclosure and quality of life among unemployed individuals with mental health problems: a longitudinal study. J Nerv Ment. 2019 Mar 1;207(3):137-9.

167     Markowitz FE. Modeling processes in recovery from mental illness: Relationships between symptoms, life satisfaction, and self-concept.J Health Soc Behav. 2001 Mar 1:64-79.

168     Szcześniak D, Kobyłko A, Wojciechowska I, Kłapciński M, Rymaszewska J. Internalized stigma and its correlates among patients with severe mental illness.Neuropsychiatr. Dis. Treat. 2018;14:2599.

169     Dickerson FB, Sommerville J, Origoni AE, Ringel NB, Parente F. Experiences of stigma among outpatients with schizophrenia. Schizophr Bull. 2002 Jan 1;28(1):143-55.

170     Alonso J, Buron A, Rojas-Farreras S, De Graaf R, Haro JM, De Girolamo G, Bruffaerts R, Kovess V, Matschinger H, Vilagut G, ESEMeD/MHEDEA 2000 Investigators. Perceived stigma among individuals with common mental disorders.J Affect Disord. 2009 Nov 1;118(1-3):180-6.

171     Manos RC, Rusch LC, Kanter JW, Clifford LM. Depression self-stigma as a mediator of the relationship between depression severity and avoidance.J Soc Clin Psychol. 2009 Nov 1;28(9):1128.

172     Kalisova L, Michalec J, Hadjipapanicolaou D, Raboch J. Factors influencing the level of self-stigmatisation in people with mental illness.Int J Soc Psychiatry. 2018 Jun;64(4):374-80.

173     Gupta MN, Mohanty S. Stigma and expressed emotion in spouses of schizophrenic patients. Indian J Community Psychol. 2016 Mar 1;12(1):98-107.

174     Habtamu K, Alem A, Medhin G, Fekadu A, Hanlon C. Functional impairment among people with severe and enduring mental disorder in rural Ethiopia: a cross-sectional study.Soc Psychiatry Psychiatr Epidemio. 2018 Aug;53(8):803-14.

175     Çapar M, Kavak F. Effect of internalized stigma on functional recovery in patients with schizophrenia. Perspect Psychiatr Care. 2019 Jan;55(1):103-11.

176     Picco L, Lau YW, Pang S, Abdin E, Vaingankar JA, Chong SA, Subramaniam M. Mediating effects of self-stigma on the relationship between perceived stigma and psychosocial outcomes among psychiatric outpatients: findings from a cross-sectional survey in Singapore. BMJ open. 2017 Aug 1;7(8):e018228.

177     Moriarty A, Jolley S, Callanan MM, Garety P. Understanding reduced activity in psychosis: the roles of stigma and illness appraisals.Soc Psychiatry Psychiatr Epidemiol. 2012 Oct;47(10):1685-93.

178     Alexová A, Kågström A, Winkler P, Kondrátová L, Janoušková M. Correlates of internalized stigma levels in people with psychosis in the Czech Republic.Int J Soc Psychiatry. 2019 Aug;65(5):347-53.

179     Sarısoy G, Kaçar ÖF, Pazvantoğlu O, Korkmaz IZ, Öztürk A, Akkaya D, Yılmaz S, Böke Ö, Sahin AR. Internalized stigma and intimate relations in bipolar and schizophrenic patients: a comparative study. Compr Psychiatry. 2013 Aug 1;54(6):665-72.

180     Lacey M, Paolini S, Hanlon MC, Melville J, Galletly C, Campbell LE. Parents with serious mental illness: Differences in internalised and externalised mental illness stigma and gender stigma between mothers and fathers. Psychiatr Res. 2015 Feb 28;225(3):723-33.

181     Cullen BA, Mojtabai R, Bordbar E, Everett A, Nugent KL, Eaton WW. Social network, recovery attitudes and internal stigma among those with serious mental illness. Int J Soc Psychiatry. 2017 Aug;63(5):448-58.

182     Prince JD, Oyo A, Mora O, Wyka K, Schonebaum AD. Loneliness among persons with severe mental illness. J Nerv Men. 2018 Feb 1;206(2):136-41.

183     Yildirim T, Kavak Budak F. The relationship between internalized stigma and loneliness in patients with schizophrenia. Perspect Psychiatr Care. 2020 Jan;56(1):168-74.

184     Adewuya AO, Owoeye AO, Erinfolami AO, Ola BA. Correlates of self-stigma among outpatients with mental illness in Lagos, Nigeria. Int J Soc Psychiatry. 2011 Jul;57(4):418-27.

185     Cerit C, Filizer A, Tural Ü, Tufan AE. Stigma: a core factor on predicting functionality in bipolar disorder. Compr Psychiatry. 2012 Jul 1;53(5):484-9.

186     Elkington KS, McKinnon K, Mann CG, Collins PY, Leu CS, Wainberg ML. Perceived mental illness stigma and HIV risk behaviors among adult psychiatric outpatients in Rio de Janeiro, Brazil.Community Ment Health J. 2010 Feb;46(1):56-64.

187     Hayward P, Wong G, Bright JA, Lam D. Stigma and self-esteem in manic depression: an exploratory study. J Affect Disord. 2002 May 1;69(1-3):61-7.

188     Pal A, Sharan P, Chadda RK. Internalized stigma and its impact in Indian outpatients with bipolar disorder. Psychiatry Res. 2017 Dec 1;258:158-65.

189     Gonzales L, Yanos PT, Stefancic A, Alexander MJ, Harney-Delehanty B. The role of neighborhood factors and community stigma in predicting community participation among persons with psychiatric disabilities. Psychiatr Serv. 2018 Jan 1;69(1):76-83.

190     Yanos PT, West ML, Gonzales L, Smith SM, Roe D, Lysaker PH. Change in internalized stigma and social functioning among persons diagnosed with severe mental illness. Psychiatry Res. 2012 Dec 30;200(2-3):1032-4.

191     Chien WT, Lam CK, Ng BF. Predictors of levels of functioning among C hinese people with severe mental illness: a 12‐month prospective cohort study.J Clin Nurs. 2015 Jul;24(13-14):1860-73.

192     Perlick DA, Rosenheck RA, Clarkin JF, Sirey JA, Salahi J, Struening EL, Link BG. Stigma as a barrier to recovery: adverse effects of perceived stigma on social adaptation of persons diagnosed with bipolar affective disorder. Psychiatr Serv. 2001 Dec;52(12):1627-32.

193     Berry C, Greenwood K. Direct and indirect associations between dysfunctional attitudes, self-stigma, hopefulness and social inclusion in young people experiencing psychosis. Schizophr Res. 2018 Mar 1;193:197-203.

194     Hipes C, Lucas J, Phelan JC, White RC. The stigma of mental illness in the labor market. Soc Sci Res. 2016 Mar 1;56:16-25.

195     Baert S, De Visschere S, Schoors K, Vandenberghe D, Omey E. First depressed, then discriminated against?. Soc Sci Med. 2016 Dec 1;170:247-54.

196     Kleintjes S, Lund C, Swartz L. Barriers to the participation of people with psychosocial disability in mental health policy development in South Africa: a qualitative study of perspectives of policy makers, professionals, religious leaders and academics.BMC Int Health Hum Rights. 2013 Dec;13(1):1-0.

197     Buizza C, Schulze B, Bertocchi E, Rossi G, Ghilardi A, Pioli R. The stigma of schizophrenia from patients' and relatives' view: A pilot study in an Italian rehabilitation residential care unit. Clin Pract Epidemiology Ment Health. 2007 Dec;3(1):1-8.

198     Kapungwe A, Cooper S, Mwanza J, Mwape L, Sikwese A, Kakuma R, Lund C, Flisher AJ. Mental illness-stigma and discrimination in Zambia. Afr J Psychiatry. 2010;13(3).

199     Ackerson BJ. Coping with the dual demands of severe mental illness and parenting: the parents' perspective. Fam Soc. 2003 Jan;84(1):109-18

200     Hall T, Kakuma R, Palmer L, Minas H, Martins J, Kermode M. Social inclusion and exclusion of people with mental illness in Timor-Leste: a qualitative investigation with multiple stakeholders. BMC Public Health. 2019 Dec;19(1):1-3.

201     Reupert A, Maybery D. Fathers’ experience of parenting with a mental illness. Fam Soc. 2009 Jan;90(1):61-8.

202     Krupchanka D, Chrtková D, Vítková M, Munzel D, Čihařová M, Růžičková T, Winkler P, Janoušková M, Albanese E, Sartorius N. Experience of stigma and discrimination in families of persons with schizophrenia in the Czech Republic. Soc Sci Med. 2018 Sep 1;212:129-35.

203     Burke E, Wood L, Zabel E, Clark A, Morrison AP. Experiences of stigma in psychosis: A qualitative analysis of service users’ perspectives. Psychosis. 2016 Apr 2;8(2):130–42.

204     Schulze B, Angermeyer MC. Subjective experiences of stigma. A focus group study of schizophrenic patients, their relatives and mental health professionals. Social science & medicine. 2003 Jan 1;56(2):299-312.

205     Lempp H, Abayneh S, Gurung D, Kola L, Abdulmalik J, Evans-Lacko S, Semrau M, Alem A, Thornicroft G, Hanlon C. Service user and caregiver involvement in mental health system strengthening in low-and middle-income countries: a cross-country qualitative study. Epidemiol Psychiatr Sci. 2018 Feb;27(1):29-39.

206     Abayneh S, Lempp H, Alem A, Alemayehu D, Eshetu T, Lund C, Semrau M, Thornicroft G, Hanlon C. Service user involvement in mental health system strengthening in a rural African setting: qualitative study. BMC Psych. 2017 Dec;17(1):1-4.

207     Gwaikolo WS, Kohrt BA, Cooper JL. Health system preparedness for integration of mental health services in rural Liberia. BMC Health Serv Res. 2017 Dec;17(1):1-0.

208     Hamilton S, Pinfold V, Cotney J, Couperthwaite L, Matthews J, Barret K, Warren S, Corker E, Rose D, Thornicroft G, Henderson C. Qualitative analysis of mental health service users’ reported experiences of discrimination. Acta Psychiatr Scand. 2016 Aug;134:14-22.

209     Tanaka C, Tuliao MT, Tanaka E, Yamashita T, Matsuo H. A qualitative study on the stigma experienced by people with mental health problems and epilepsy in the Philippines. BMC Psych. 2018 Dec;18(1):1-3.

210     Egbe CO, Brooke-Sumner C, Kathree T, Selohilwe O, Thornicroft G, Petersen I. Psychiatric stigma and discrimination in South Africa: perspectives from key stakeholders. BMC Psych. 2014 Dec;14(1):1-4.

211     Lyons C, Hopley P, Horrocks J. A decade of stigma and discrimination in mental health: plus ça change, plus c'est la même chose (the more things change, the more they stay the same).J Psychiatr Ment Health Nurs. 2009 Aug;16(6):501-7.

212     McColl L. ‘They just don’t care’: The experiences of mental health consumers in a Queensland bush community.AeJAMH. 2007 Jan 17;6(2):138–46.

213     González-Torres MA, Oraa R, Arístegui M, Fernández-Rivas A, Guimon J. Stigma and discrimination towards people with schizophrenia and their family members. Soc Psychiatry Psychiatr Epidemiol. 2007 Jan 11;42(1):14–23.

214     Ferrari M, Flora N, Anderson KK, Tuck A, Archie S, Kidd S, et al. The African, Caribbean and European (ACE) Pathways to Care study: a qualitative exploration of similarities and differences between African-origin, Caribbean-origin and European-origin groups in pathways to care for psychosis. BMJ Open. 2015 Jan 14;5(1):e006562–e006562.

215     Lakeman R, McGowan P, MacGabhann L, Parkinson M, Redmond M, Sibitz I, et al. A qualitative study exploring experiences of discrimination associated with mental-health problems in Ireland. Epidemiol Psychiatr Sci. 2012 Sep 9;21(3):271–9.

216     Byatt N, Biebel K, Friedman L, Debordes-Jackson G, Ziedonis D, Pbert L. Patient’s views on depression care in obstetric settings: how do they compare to the views of perinatal health care professionals? Gen Hosp Psychiatry. 2013 Nov;35(6):598–604.

217     Bye A, Shawe J, Bick D, Easter A, Kash-Macdonald M, Micali N. Barriers to identifying eating disorders in pregnancy and in the postnatal period: a qualitative approach. BMC Pregnancy and Childbirth. 2018 Dec 15;18(1):114.

218     Krupchanka D, Chrtková D, Vítková M, Munzel D, Čihařová M, Růžičková T, et al. Experience of stigma and discrimination in families of persons with schizophrenia in the Czech Republic. Soc Sci Med. 2018 Sep;212:129–35.

219     Hamilton S, Pinfold V, Cotney J, Couperthwaite L, Matthews J, Barret K, et al. Qualitative analysis of mental health service users’ reported experiences of discrimination. Acta Psychiatr Scand. 2016 Aug 17;134(S446):14–22.

220     Cárcamo Guzmán K, Cofré Lira I, Flores Oyarzo G, Lagos Arriagada D, Oñate Vidal N, Grandón Fernández P. Atención en salud mental de las personas con diagnóstico psiquiátrico grave y su recuperación. Psicoperspectivas. 2019 Jul 13;18(2).

221     Farrelly S, Jeffery D, Rüsch N, Williams P, Thornicroft G, Clement S. The link between mental health-related discrimination and suicidality: service user perspectives. Psychol Med. 2015 Jul 13;45(10):2013–22.

222     Howe L, Tickle A, Brown I. ‘Schizophrenia is a dirty word’: service users' experiences of receiving a diagnosis of schizophrenia. Psychiatr Bull. 2014 Aug;38(4):154-8.

223     Chung KF, Tse S, Lee CT, Chan WM. Changes in stigma experience among mental health service users over time: a qualitative study with focus groups. Community Ment Health J. 2019 Nov;55(8):1389-94.

224     Yuksel C, Bingol FA, Oflaz F. ‘Stigma: the cul‐de‐sac of the double bind’the perspective of T urkiye; a phenomenological study. J Psychiatr Ment Health Nurs. 2014 Oct;21(8):667-78

225     Rezaie L, Shafaroodi N, Philips D. The barriers to participation in leisure time physical activities among Iranian women with severe mental illness: A qualitative study. Ment Health Phys Act. 2017 Oct 1;13:171-7.

226     Windell D, Norman RM. A qualitative analysis of influences on recovery following a first episode of psychosis. Int J Soc Psychiatry. 2013 Aug;59(5):493-500.

227     Rose D, Willis R, Brohan E, Sartorius N, Villares C, Wahlbeck K, Thornicroft GI. Reported stigma and discrimination by people with a diagnosis of schizophrenia.Epidemiol Psychiatr Sci. 2011 Jun;20(2):193-204.

228     Interian A, Martinez IE, Guarnaccia PJ, Vega WA, Escobar JI. A Qualitative Analysis of the Perception of Stigma Among Latinos Receiving Antidepressants. Psychiatr Serv. 2007 Dec;58(12):1591–4.

229     Dinos S, Stevens S, Serfaty M, Weich S, King M. Stigma: the feelings and experiences of 46 people with mental illness. BJPsych. 2004 Feb 2;184(2):176–81.

230     Srimongkon P, Aslani P, Chen TF. Consumer-related factors influencing antidepressant adherence in unipolar depression: a qualitative study. Patient Prefer Adherence. 2018;12:1863–73.

231     Kranke D, Floersch J, Townsend L, Munson M. Stigma experience among adolescents taking psychiatric medication. Child Youth Serv Rev. 2010 Apr;32(4):496–505.

232     Conrad MM, Pacquiao DF. Manifestation, Attribution, and Coping With Depression Among Asian Indians From the Perspectives of Health Care Practitioners. J Transcult Nurs. 2005 Jan 23;16(1):32–40

233     Kranke DA, Floersch J, Kranke BO, Munson MR. A qualitative investigation of self-stigma among adolescents taking psychiatric medication. Psychiatric Services. 2011 Aug;62(8):893-9.

234     Teferra S, Hanlon C, Beyero T, Jacobsson L, Shibre T. Perspectives on reasons for non-adherence to medication in persons with schizophrenia in Ethiopia: a qualitative study of patients, caregivers and health workers. BMC Psych. 2013 Dec;13(1):1-9.

235     Samuel IA. Utilization of mental health services among African-American male adolescents released from juvenile detention: Examining reasons for within-group disparities in help-seeking behaviors.Child Adolesc Social Work J. 2015 Feb;32(1):33-43.

236     Olawande TI, Ajayi MP, Amoo EO, Iruonagbe CT. Variations in family attitudes towards coping with people living with mental illness.Open Access Maced J Med Sci. 2019 Jun 6;7(12):2010.

237     Lin CL, Kopelowicz A, Chan CH, Hsiung PC. A qualitative inquiry into the Taiwanese mentally ill persons' difficulties living in the community.Arch Psychiatr Nurs. 2008 Oct 1;22(5):266-76.

238     Lloyd C, Sullivan D, Williams PL. Perceptions of social stigma and its effect on interpersonal relationships of young males who experience a psychotic disorder.Aust Occup Ther J. 2005 Sep;52(3):243-50.

239     Kaewprom C, Curtis J, Deane FP. Factors involved in recovery from schizophrenia: a qualitative study of Thai mental health nurses. Nurs Health Sci. 2011 Sep;13(3):323-7.

240     Crowe A, Averett P, Glass JS. Mental illness stigma, psychological resilience, and help seeking: What are the relationships?. Men Health Prev. 2016 Jun 1;4(2):63-8.

241     Polacsek M, Boardman GH, McCann TV. Help‐seeking experiences of older adults with a diagnosis of moderate depression. Int J Ment Health Nurs. 2019 Feb;28(1):278-87.

242     Lin SY. Beliefs about causes, symptoms, and stigma associated with severe mental illness among ‘highly acculturated’Chinese-American patients. Int J Soc Psychiatry. 2013 Dec;59(8):745-51

243     Hill SK, Cantrell P, Edwards J, Dalton W. Factors influencing mental health screening and treatment among women in a rural south central Appalachian primary care clinic. J Rural Health. 2016 Jan;32(1):82-91.

244     Jansen JE, Wøldike PM, Haahr UH, Simonsen E. Service user perspectives on the experience of illness and pathway to care in first-episode psychosis: a qualitative study within the TOP project. Psychiatr Quart. 2015 Mar;86(1):83-94.

245     Fernandez y Garcia E, Duberstein P, Paterniti DA, Cipri CS, Kravitz RL, Epstein RM. Feeling labeled, judged, lectured, and rejected by family and friends over depression: Cautionary results for primary care clinicians from a multi-centered, qualitative study. BMC Fam Pract. 2012 Dec;13(1):1-9.

246     Blignault I, Ponzio V, Rong Y, Eisenbruch M. A qualitative study of barriers to mental health services utilisation among migrants from mainland China in South-East Sydney. Int J Soc Psychiatry. 2008 Mar;54(2):180-90

247     Holley LC, Oh H, Thomas DS. Mental illness discrimination and support experienced by people who are of color and/or LGB: Considering intersecting identities. Am J Orthopsychiatry. 2019;89(1):16.

248     Pettersen G, Rosenvinge JH, Ytterhus B. The “double life” of bulimia: Patients’ experiences in daily life interactions. Eating Disord. 2008 May 6;16(3):204-11.

249     Koschorke M, Padmavati R, Kumar S, Cohen A, Weiss HA, Chatterjee S, Pereira J, Naik S, John S, Dabholkar H, Balaji M. Experiences of stigma and discrimination of people with schizophrenia in India. Soc Sci Med. 2014 Dec 1;123:149-59.

250     Nxumalo Ngubane S, McAndrew S, Collier E. The experiences and meanings of recovery for Swazi women living with “schizophrenia”.J Psychiatr Ment Health Nurs. 2019 Jun;26(5-6):153-62.

251     Chan SY, Ho GW, Bressington D. Experiences of self‐stigmatization and parenting in Chinese mothers with severe mental illness. Int J Ment Health Nurs. 2019 Apr;28(2):527-37.

252     Karanci NA, Aras A, Kumpasoğlu GB, Can D, Çakır E, Karaaslan C, Semerci M, Tüzün D. Living with schizophrenia: Perspectives of Turkish people with schizophrenia from two Patient Associations on how the illness affects their lives. Int J Soc Psychiatry. 2019 Mar;65(2):98-106.

253     Elliott L, Bennett AS, Szott K, Golub A. Competing constructivisms: the negotiation of PTSD and related stigma among post-9/11 veterans in New York City. Cult Med Psychiatry. 2018 Dec;42(4):778-99.

254     Eapen V, Ghubash R. Help-seeking for mental health problems of children: preferences and attitudes in the United Arab Emirates. Psychol Rep. 2004 Apr;94(2):663-7

255     Crumb L, Mingo TM, Crowe A. “Get over it and move on”: the impact of mental illness stigma in rural, low-income United States populations. Ment Health Prev. 2019 Mar 1;13:143-8.

256     Lueck JA. What’s the risk in seeking help for depression? Assessing the nature and pleasantness of outcome perceptions among individuals with depressive symptomatology. Health Educ Behav. 2019 Jun;46(3):463-70.

257     Marquez JA, Ramírez García JI. Family caregivers’ narratives of mental health treatment usage processes by their Latino adult relatives with serious and persistent mental illness. J Fam Psychol. 2013 Jun;27(3):398.

258     Robinson WD, Springer PR, Bischoff R, Geske J, Backer E, Olson M, Jarzynka K, Swinton J. Rural experiences with mental illness: Through the eyes of patients and their families. Fam Syst Health. 2012 Dec;30(4):308.

259     Marthoenis M, Aichberger MC, Schouler-Ocak M. Patterns and determinants of treatment seeking among previously untreated psychotic patients in Aceh Province, Indonesia: a qualitative study. Scientifica. 2016 Jan 1;2016.

260     Olasoji M, Maude P, McCauley K. A journey of discovery: experiences of carers of people with mental illness seeking diagnosis and treatment for their relative. Issues Ment Health Nurs. 2016 Apr 2;37(4):219-28.

261     Loganathan S, Murthy RS. Living with schizophrenia in India: gender perspectives. Transcult Psychiatry. 2011 Nov;48(5):569-84.

262     Russinova Z, Griffin S, Bloch P, Wewiorski NJ, Rosoklija I. Workplace prejudice and discrimination toward individuals with mental illnesses. J Vocat Rehabil. 2011 Jan 1;35(3):227-41.

263     Elkington KS, Hackler D, McKinnon K, Borges C, Wright ER, Wainberg ML. Perceived mental illness stigma among youth in psychiatric outpatient treatment. J Adolesc Res. 2012 Mar;27(2):290-317.

264     Koskela SA, Pettitt B, Drennan VM. The experiences of people with mental health problems who are victims of crime with the police in England: A qualitative study. Br J Criminol. 2016 Sep 1;56(5):1014-33.

265     Liegghio M, Jaswal P. Police encounters in child and youth mental health: could stigma informed crisis intervention training (CIT) for parents help?. J Soc Work Practic. 2015 Jul 3;29(3):301-19.

266     Elkington KS, Hackler D, Walsh TA, Latack JA, McKinnon K, Borges C, Wright ER, Wainberg ML. Perceived mental illness stigma, intimate relationships, and sexual risk behavior in youth with mental illness. J Adolesc Res. 2013 May;28(3):378-404.

267     Fennell D, Liberato AS. Learning to live with OCD: Labeling, the self, and stigma. Deviant Behav. 2007 May 11;28(4):305-31.

268     Loganathan S, Murthy SR. Experiences of stigma and discrimination endured by people suffering from schizophrenia. Indian J Psychiatry. 2008 Jan;50(1):39.

269     Moses T. Being treated differently: Stigma experiences with family, peers, and school staff among adolescents with mental health disorders. Soc Sci Med. 2010 Apr 1;70(7):985-93.

270     Mathias K, Kermode M, Sebastian MS, Koschorke M, Goicolea I. Under the banyan tree-exclusion and inclusion of people with mental disorders in rural North India. BMC Public Health. 2015 Dec;15(1):1-1.

271     Pinfold V, Byrne P, Toulmin H. Challenging stigma and discrimination in communities: a focus group study identifying UK mental health service users’ main campaign priorities. Int J Soc Psychiatry. 2005 Jun;51(2):128-38.

272     Wong YL, Kong D, Tu L, Frasso R. “My bitterness is deeper than the ocean”: understanding internalized stigma from the perspectives of persons with schizophrenia and their family caregivers. Int J Ment Health Syst. 2018 Dec;12(1):1-5.

273     Heydari A, Saadatian V, Soodmand P. Black shadow of stigma: lived experiences of patients with psychiatric disorders on the consequences of stigma. Iran J Psychiatry Behav Sci. 2017 Sep 30;11(3).

274     Ladd W. " Born Out of Fear": A Grounded Theory Study of the Stigma of Bipolar Disorder for New Mothers. Qualitative Report. 2018 Sep 1;23(9).

275     Paul S. Responses to stigma-related stressors: a qualitative inquiry into the lives of people living with schizophrenia in India. Int J Cult Ment Health. 2016 Jul 2;9(3):261-77.

276     Mora-Ríos J, Bautista N. Estigma estructural, género e interseccionalidad. Implicaciones en la atención a la salud mental. Salud mental. 2014;37(4):303-12.

277     Lund C, Kleintjes S, Cooper S, Petersen I, Bhana A, Flisher AJ, MHaPP Research Programme Consortium. Challenges facing South Africa's mental health care system: stakeholders' perceptions of causes and potential solutions. Int J Cult Ment Health. 2011 Jun 1;4(1):23-38

278     Rezayat F, Mohammadi E, Fallahi‐khoshknab M, Sharifi V. Experience and the meaning of stigma in patients with schizophrenia spectrum disorders and their families: A qualitative study. Jpn J Nurs Sci. 2019 Jan;16(1):62-70.

279     Brophy LM, Roper CE, Hamilton BE, Tellez JJ, McSherry BM. Consumers and their supporters’ perspectives on poor practice and the use of seclusion and restraint in mental health settings: results from Australian focus groups. Int J Ment Health Syst. 2016 Dec;10(1):1-0.

280     Dikeç G, Uzunoğlu G, Gümüş F. Stigmatization experiences of Turkish parents of patients hospitalized in child and adolescent psychiatric clinics. Perspect Psychiatr Care. 2019 Jan 25;55(2):336-43.

281     Gladman B, Waghorn G. Personal experiences of people with serious mental illness when seeking, obtaining and maintaining competitive employment in Queensland, Australia. Work. 2016 Jan 1;53(4):835-43.

282     Nithsdale V, Davies J, Croucher P. Psychosis and the experience of employment. J Occup Rehabil. 2008 Jun;18(2):175-82.

283     Hamilton S, Lewis-Holmes E, Pinfold V, Henderson C, Rose D, Thornicroft G. Discrimination against people with a mental health diagnosis: qualitative analysis of reported experiences. J Ment Health. 2014 Apr 1;23(2):88-93.

284     Michalak EE, Yatham LN, Maxwell V, Hale S, Lam RW. The impact of bipolar disorder upon work functioning: a qualitative analysis. Bipolar Disorders. 2007 Feb;9(1‐2):126-43

285     Ebuenyi ID, Guxens M, Ombati E, Bunders-Aelen JF, Regeer BJ. Employability of persons with mental disability: Understanding lived experiences in Kenya. Front Psychiatry. 2019 Jul 30;10:539.

286      Forchuk C, Nelson G, Hall GB. “It's important to be proud of the place you live in”: Housing problems and preferences of psychiatric survivors. Perspect Psychiatr Care. 2006 Feb;42(1):42-52.

287      Muroff JR, Hoerauf SL, Kim SY. Is psychiatric research stigmatized? An experimental survey of the public.Schizophr. Bull. 2006.**1**(1):129-136.

288      Stone EM, McGinty EE. Public willingness to pay to improve services for individuals with serious mental illness. Psychiatr Serv. 2018 Aug 1;**69**(8):938-41.

289       Mossakowski KN, Kaplan LM, Hill TD. Americans’ attitudes toward mental illness and involuntary psychiatric medication. Soc MentHealth. 2011 Nov;**1**(3):200-16.

290       Noe SR. Discrimination against individuals with mental illness. J Rehabil. 1997;**63**(1):20.

291       O'Reilly CL, Bell JS, Kelly PJ, Chen TF. Exploring the relationship between mental health stigma, knowledge and provision of pharmacy services for consumers with schizophrenia. Res Social Adm Pharmacy. 2015 May 1;**11**(3):e101-9

292       Magliano L, Read J, Sagliocchi A, Patalano M, Oliviero N. Effect of diagnostic labeling and causal explanations on medical students' views about treatments for psychosis and the need to share information with service users. Psychiatry Res. 2013 Dec 15;**210**(2):402-7.

293       Magliano L, Punzo R, Strino A, Acone R, Affuso G, Read J. General practitioners’ beliefs about people with schizophrenia and whether they should be subject to discriminatory treatment when in medical hospital: The mediating role of dangerousness perception. Am J Orthopsychiatry. 2017;**87**(5):559.

294       Magliano L, Citarelli G, Read J. The beliefs of non‐psychiatric doctors about the causes, treatments, and prognosis of schizophrenia. Psychol Psychoter. 2020 Dec;**93**(4):674-89.

295      Magliano L, Strino A, Punzo R, Acone R, Affuso G, Read J. Effects of the diagnostic label ‘schizophrenia’, actively used or passively accepted, on general practitioners’ views of this disorder. Int J Soc Psychiatry. 2017 May;**63**(3):224-34.

296       Mackay N, Barrowclough C. Accident and emergency staff's perceptions of deliberate self‐harm: Attributions, emotions and willingness to help. BJPsych. 2005 Jun;**44**(2):255-67.

297       Maidment R, Livingston G, Katona C, McParland M, Noble L. Change in attitudes to psychiatry and intention to pursue psychiatry as a career in newly qualified doctors: a follow-up of two cohorts of medical students. Medical teacher. 2004 Sep 1;**26**(6):565-9.

298       Mötteli S, Horisberger R, Lamster F, Vetter S, Seifritz E, Jäger M. More optimistic recovery attitudes are associated with less stigmatization of people with mental illness among healthcare professionals working on acute and semi-acute psychiatric wards. Psychiatric Quart. 2019 Sep;**90**(3):481-9.

299       Sabin JA, Stuber J, Rocha A, Greenwald A. Providers’ implicit and explicit stereotypes about mental illnesses and clinical competencies in recovery. Soc Work Ment Health. 2015 Sep 3;**13**(5):495-513.

300       Manuel JI, Appelbaum PS, Le Melle SM, Mancini AD, Huz S, Stellato CB, Finnerty MT. Use of intervention strategies by assertive community treatment teams to promote patients’ engagement. Psychiatr Serv. 2013 Jun;**64**(6):579-85.

301       MacNeela P, Scott PA, Treacy M, Hyde A, O'Mahony R. A risk to himself: Attitudes toward psychiatric patients and choice of psychosocial strategies among nurses in medical–surgical units. Res Nurs Health. 2012 Apr;**35(**2):200-13.

302       Minas H, Zamzam R, Midin M, Cohen A. Attitudes of Malaysian general hospital staff towards patients with mental illness and diabetes. BMC Pub Health. 2011 Dec;**11**(1):1-0.

303       Rickles NM, Dube GL, McCarter A, Olshan JS. Relationship between attitudes toward mental illness and provision of pharmacy services.J Am Pharm Assoc. 2010 Nov 1;**50**(6):704-13.

304       Reed F, Fitzgerald L. The mixed attitudes of nurse's to caring for people with mental illness in a rural general hospital. Int J Ment Health Nurs. 2005 Dec;**14**(4):249-57.

305       Ordan R, Shor R, Liebergall‐Wischnitzer M, Noble L, Noble A. Nurses’ professional stigma and attitudes towards postpartum women with severe mental illness. J Clin Nurs. 2018 Apr;**27**(7-8):1543-51.
